# Multi-omics examination of Q fever fatigue syndrome identifies similarities with chronic fatigue syndrome

**DOI:** 10.1186/s12967-020-02585-5

**Published:** 2020-11-26

**Authors:** Ruud P. H. Raijmakers, Megan E. Roerink, Anne F. M. Jansen, Stephan P. Keijmel, Ranko Gacesa, Yang Li, Leo A. B. Joosten, Jos W. M. van der Meer, Mihai G. Netea, Chantal P. Bleeker-Rovers, Cheng-Jian Xu

**Affiliations:** 1 Division of Infectious Diseases 463, Department of Internal Medicine, Radboud Expertise Center for Q Fever, Radboud University Medical Center, P.O. Box 9101, 6500 HB Nijmegen, The Netherlands; 2 Department of Internal Medicine, Radboud University Medical Center, Nijmegen, The Netherlands; 3 Department of Genetics, University Medical Center Groningen, Groningen, The Netherlands; 4 Centre for Individualised Infection Medicine, CiiM, A Joint Venture between the Hannover Medical School and the Helmholtz Centre for Infection Research, Hannover, Germany; 5 TWINCORE, Centre for Experimental and Clinical Infection Research, A Joint Venture between the Hannover Medical School and the Helmholtz Centre for Infection Research, Hannover, Germany; 6 Radboud Center for Infectious Diseases, Radboud University Medical Center, Nijmegen, The Netherlands

**Keywords:** QFS, CFS, Fatigue, Q fever, Omics, Inflammation, Metabolome, Microbiome

## Abstract

**Background:**

Q fever fatigue syndrome (QFS) is characterised by a state of prolonged fatigue that is seen in 20% of acute Q fever infections and has major health-related consequences. The molecular mechanisms underlying QFS are largely unclear. In order to better understand its pathogenesis, we applied a multi-omics approach to study the patterns of the gut microbiome, blood metabolome, and inflammatory proteome of QFS patients, and compared these with those of chronic fatigue syndrome (CFS) patients and healthy controls (HC).

**Methods:**

The study population consisted of 31 QFS patients, 50 CFS patients, and 72 HC. All subjects were matched for age, gender, and general geographical region (South-East part of the Netherlands). The gut microbiome composition was assessed by Metagenomic sequencing using the Illumina HiSeq platform. A total of 92 circulating inflammatory markers were measured using Proximity Extension Essay and 1607 metabolic features were assessed with a high-throughput non-targeted metabolomics approach.

**Results:**

Inflammatory markers, including 4E-BP1 (*P* = 9.60^–16^ and 1.41^–7^) and MMP-1 (*P* = 7.09^–9^ and 3.51^–9^), are significantly more expressed in both QFS and CFS patients compared to HC. Blood metabolite profiles show significant differences when comparing QFS (319 metabolites) and CFS (441 metabolites) patients to HC, and are significantly enriched in pathways like sphingolipid (*P* = 0.0256 and 0.0033) metabolism. When comparing QFS to CFS patients, almost no significant differences in metabolome were found. Comparison of microbiome taxonomy of QFS and CFS patients with that of HC, shows both in- and decreases in abundancies in *Bacteroidetes* (with emphasis on *Bacteroides* and *Alistiples* spp.), and *Firmicutes* and *Actinobacteria* (with emphasis on *Ruminococcus* and *Bifidobacterium* spp.). When we compare QFS patients to CFS patients, there is a striking resemblance and hardly any significant differences in microbiome taxonomy are found.

**Conclusions:**

We show that QFS and CFS patients are similar across three different omics layers and 4E-BP1 and MMP-1 have the potential to distinguish QFS and CFS patients from HC.

## Background

Q fever fatigue syndrome (QFS) is characterised as a state of prolonged fatigue following acute Q fever infection [1]. The fatigue lasts for at least 6 months and is usually associated with musculoskeletal complaints, neurocognitive problems, sleeping problems, headache, respiratory tract problems and mood disorders [1]. QFS was first described by Shannon et al. in 1993 and occurs worldwide [2, 3]. In many ways, complaints of QFS are similar to those of chronic fatigue syndrome (CFS) [4, 5], and the pathogenesis of these syndromes remains largely unclear. An important distinction between QFS and CFS is the fact that QFS has a known aetiology, being precipitated by an acute Q fever infection, and is therefore considered to be a postinfectious fatigue syndrome.

Symptomatic infection with *Coxiella burnetii* is called acute Q fever and constitutes around 40% of human primary *Coxiella* infections: the other 60% remain asymptomatic [6–8]. Acute Q fever often is a self-limiting disease that usually presents as a flu-like illness that may be accompanied by pneumonia or hepatitis [7]. Of all those who become infected with *C. burnetii*, both symptomatic and asymptomatic, around 1–5% develop a persistent infection with the bacterium, called chronic Q fever or persistent focalised infection [9]. Of all those who develop acute Q fever, i.e., symptomatic, infection, around 20% will develop QFS [1].

Like CFS [10], some studies of QFS suggest that there is a low-grade inflammatory component. First reports supporting this notion came from Penttilla et al. in 1998, who showed that peripheral blood mononuclear cells (PBMCs) of QFS patients produce more IL-6 when stimulated with Q fever antigen than controls [11]. During the Dutch Q fever outbreak (2007–2010), our group demonstrated that QFS patients exhibit signs of altered immunity through the monocyte-derived cytokines Tumor Necrosis Factor (TNF)α, interleukin (IL)-1β, and especially IL-6, together with the interferon (IFN)γ-axis [12–14]. In addition, we found that monocytes of both QFS and CFS patients show decreased expression of mitochondrial derived peptide (MDP)-coding genes *MT-RNR1* and *MT-RNR2*, resulting in a decreased production of humanin (*MT-RNR2*) [15].

Investigations of the metabolome [16–20] and (gut) microbiome [21–25] in CFS, both aspects that are inadvertently linked with inflammation [16, 26], showed interesting, albeit inconsistent, results [23, 25]. Up until this moment, no such studies were conducted in QFS.

To better understand the various molecular aspects of QFS aetiology and its place in the chronic fatigue syndrome spectrum, we applied a multi-omics approach and investigated the inflammatory proteome, metabolome, and composition of gut microbiome of QFS patients, CFS patients, and healthy controls (HC), matched for age, gender, use of medication, and general geographic region.

## Methods

### Study population

The study population consisted of QFS patients (n = 31), CFS patients (n = 50), and HC (n = 72). All subjects were matched for age (± 10 years), gender (non-pregnant females), and general geographical region (south-eastern part of the Netherlands). QFS patients were actively recruited for this study while CFS patients and HC participated in previous studies. As all CFS patients included in this study are non-pregnant females, so were recruited QFS patients and selected HC. All subjects were between 18 and 59 years of age and did not use medication, other than paracetamol or oral contraceptives, and were not vaccinated, in the last 6 months. Both QFS and CFS patients were diagnosed according to similar guidelines as described previously [27, 28] with the only difference on whether complaints were precipitated by an acute Q fever infection or not.

All QFS patients were diagnosed at the Radboud Expert Center for Q fever, Nijmegen, the Netherlands, after a uniform work-up according to the Dutch guideline on QFS diagnosis [29]. All QFS patients met the following diagnostic criteria: (i) fatigue that lasted ≥ 6 months; (ii) sudden onset of severe fatigue [defined as a score ≥ 35 on the subscale fatigue severity of the Checklist Individual Strength (CIS) questionnaire], or significant increase in fatigue, both related to a symptomatic acute Q fever infection; (iii) chronic Q fever and other somatic or psychiatric causes of fatigue were excluded; and (iv) fatigue resulted in significant functional impairment [defined as a total score ≥ 450 on the Sickness Impact Profile-8 (SIP-8) questionnaire]. All QFS patients were fatigued for less than 10 years.

All CFS patients were diagnosed at the Department of Internal Medicine and Expert Center for Chronic Fatigue (ECCF) of the Radboud University Medical Center, Nijmegen, the Netherlands, after a uniform work-up according to the Centers for Disease Control (CDC) criteria for CFS. All CFS patients participated in a randomized trial on cytokine inhibition in CFS and samples were collected at baseline, prior to the intervention [30]. All CFS patients had a score ≥ 35 on the subscale fatigue severity of the CIS questionnaire and a score ≥ 450 on the SIP-8 questionnaire. All CFS patients were fatigued for less than 10 years.

All HC reported having no complaints of fatigue and participated in the Human Functional Genomics Project (www.humanfunctionalgenomics.org) [31]. Samples were collected at the Radboud University Medical Center, Nijmegen, the Netherlands. All HC were selected based on age, gender and general geographical region that matched with both QFS and CFS patient groups. For proteomics and metagenomics, a larger HC group (n = 50) was used than for metabolomics data (n = 22). For evaluating the correlation patterns between metabolome and inflammatory proteome, an independent population-based cohort from the same general geographical region (n = 318) was used for comparison.

### Sample handling and omics measurements

Faecal and plasma samples were collected in 2016 and 2017 as previously described [30, 32]. Venous blood was collected in EDTA tubes, and kept on ice until centrifugation, which was performed within 2–3 h. Next, samples were centrifuged at 2960×*g* for 10 min at 4 °C. Plasma aliquots were then frozen at − 80 °C for a maximal duration of 2 years. Metabolome and inflammatory proteome analyses for all patients and controls were run at the same time. Protein levels were measured in 131 participants and determined using Olink inflammation panel, including 92 inflammation-related protein biomarkers (https://www.olink.com/). During the quality control step, inflammatory markers with > 20% of measurements below the detection limit were excluded for further analysis, leaving 65 proteins in total. Serum metabolite levels were measured in General Metabolomics platforms (https://generalmetabolics.com) for all 131 individuals. Metabolome was measured and annotated by General Metabolomics (Boston, MA) using flow injection-time-of-flight mass (flow-injection TOF-M) spectrometry. Faecal samples were collected within 24 h before processing and cooled at 4 °C before processing. Samples were aliquoted and then frozen at − 80 °C for a maximal duration of 2 years. The gut metagenomic sequencing was performed at Novogene, China, using the Illumina HiSeq platform. The metagenome profiling was measured according to a previously described protocol [33]. KneadData tools (v0.5.1) [34] were used to remove the adapters, trim the sequencing reads to PHRED quality 30, and remove reads aligned to human genome (GRCh37/hg19).

Taxonomy of metagenomes was profiled using MetaPhlAn2 (v2.7.2) [35], and the microbial biochemical pathways were profiled using HUMAnN2 pipeline (v0.11.1) [36] integrated with DIAMOND alignment tool (v0.8.22) [37], ChocoPhlAn database (v0.1.1) and Uniref90 database (v0.1.1) [38]. After filtering for quality, 131 measurements of gut metagenomes were used in the subsequent analyses. HC DNA was stored longer, i.e. approximately 2 years, than QFS and CFS DNA. All samples were processed according to the same protocols and sequencing was performed at the same time based on a randomised experimental design.

### PBMC stimulation and cytokine assay

PBMC isolation was performed by dilution of blood in PBS (1:1) and fractions were separated by density centrifugation over Ficoll-Paque (Ficoll-Paque Plus; GE healthcare). Cells were washed three times with cold PBS and resuspended in Roswell Park Memorial Institute (RPMI) 1640 Dutch modification culture medium (Life Technologies/Invitrogen) supplemented with 50 μg/mL gentamicin, 2 mM Glutamax™, and 1 mM pyruvate (Life Technologies). PBMCs were then plated in 96-well round-bottom plates (Corning) at a concentration of 5 × 10^5^/mL in a total volume of 200 µL. Cells were exposed to RPMI, as a negative control, 0.5 mM l-cysteine, and 25 mM l-cysteinylglycine for 24 h at 37 °C with 5% CO_2_. After stimulation, supernatants were collected and MCP-1 and TGF-β were measured using enzyme-linked immune sorbent assay (ELISA) according to the manufacturer’s protocol (R&D Systems).

### Statistical analysis

Patient characteristics data were analysed using Graphpad Prism (Graphpad Software Inc., version 5.03). ANOVA was used to determine differences between groups. For the correlation analyses, Spearman’s Rank-Order correlation coefficients were used followed by hierarchical clustering. R package ‘corrplot’ was used for visualization. Cytokine production data were analysed using the Mann–Whitney U test in GraphPad Prism (Graphpad Software Inc., version 5.03). The differential proteome and metabolome analyses were conducted using robust linear regression [39] with age effect corrected. For prediction model, Least Absolute Shrinkage and Selection Operator (LASSO) model [40] was utilized. Repeated Cross validation (CV) approach was used for building prediction models: 2/3 of samples were randomly selected for training while the rest of samples were used for prediction. The procedure was repeated 1000 times, and the Area under the curve (AUC) was calculated to evaluate the predictive power of the model (Additional file 1: Figure S1). The metabolic pathway enrichment analysis was performed online by using MetaboAnalyst 4.0 [41]. A principal coordinates analysis (PCoA) was performed on gut microbiome taxonomy. All statistical analyses were performed using the computing environment R (version 3.5.3). Statistical significance was obtained if *P* ≤ 0.05. To account for multiple testing, we assessed significance using Benjamini–Hochberg false discovery rate (FDR < 0.05).

### Ethical statement

All participants provided written informed consent and the study, including studies from which CFS patients and HC were protracted [30, 32], was approved by the Medical Ethical Review Committee of the Arnhem-Nijmegen region.

## Results

### Subject characteristics

All QFS and CFS patients were severely fatigued and functionally impaired at the time of sample collection. Mean fatigue severity scores were significantly higher for CFS patients compared to QFS patients (Student’s *t* test, *P* = 0.0034). No significant differences in mean functional impairment scores were observed when comparing QFS patients with CFS patients (Student’s *t* test, *P* = 0.3055) (Table 1).

**Table 1:** Characteristics of QFS patients, CFS patients, and HC

Characteristics	QFS (n = 31)	CFS (n = 50)	HC (n = 72)
Female sex, number (%)	31 (100)	50 (100)	72 (100)
Age (years), mean ± SD	44.2 ± 10.5	31.0 ± 10.1	37.5 ± 13.3
BMI, mean ± SD	25.4 ± 4.6	24.8 ± 4.3	23.2 ± 3.0
Sickness duration, months, mean ± SD	94.2 ± 15.9	46.6 ± 31.2	–
CIS subscale fatigue severity score, mean ± SD	48.3 ± 5.6	51.5 ± 4.1	–
SIP-8 total score, mean ± SD	1518 ± 711.1	1677 ± 650.1	–

Characteristics of QFS patients, CFS patients, and HC. Other than the percentage of participants of female sex, results are depicted as mean ± SD

*QFS* Q fever fatigue syndrome, *HC* healthy controls, *CFS* chronic fatigue syndrome, *CIS* Checklist Individual Strength, *SD* standard deviation, *SIP-8* Sickness Impact Profile-8

### QFS and CFS show expression profiles of inflammatory proteins distinct from HC

**Fig. 1:**
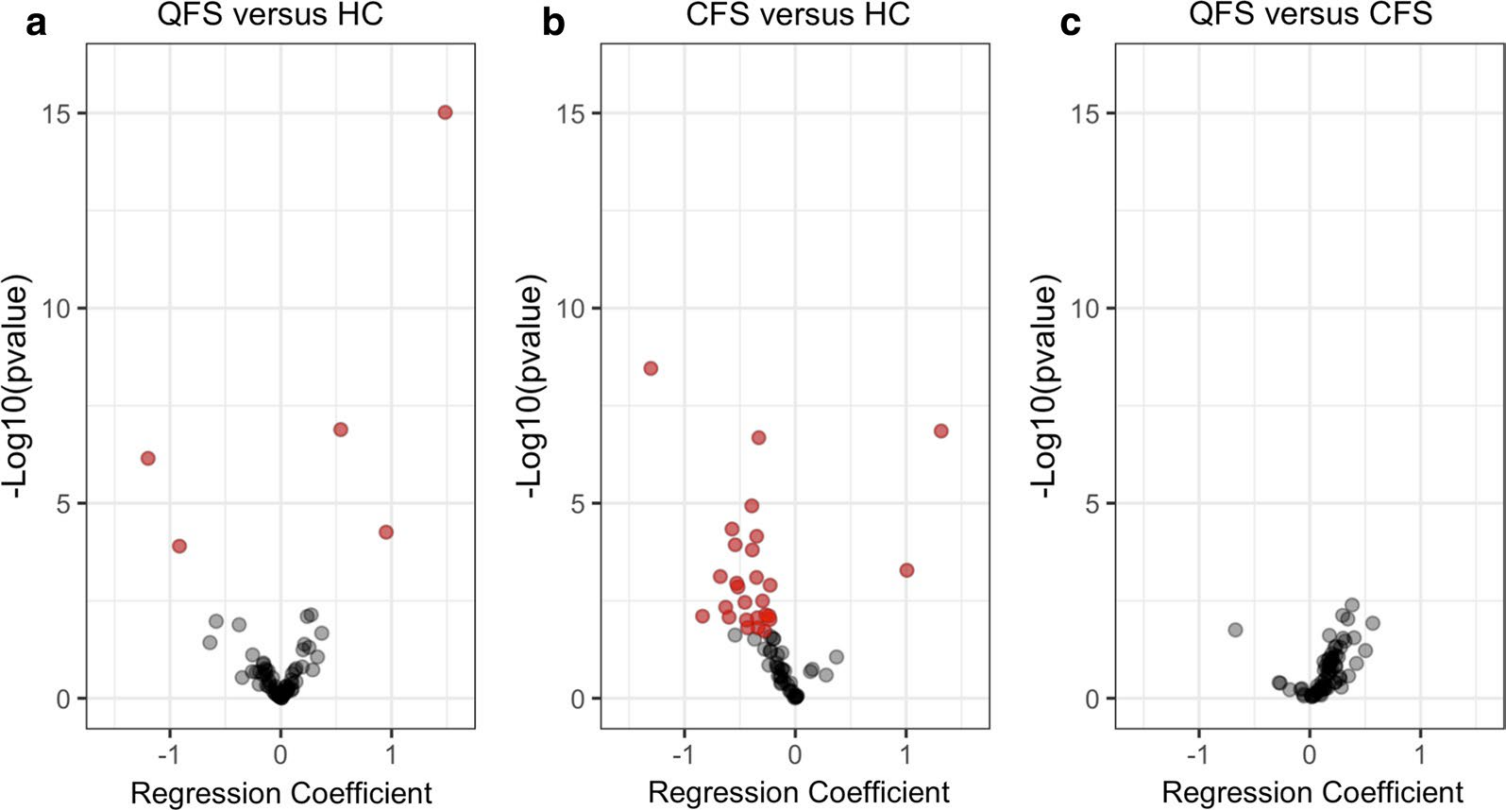
Differential expression of in- and decreased circulating inflammatory proteins when comparing QFS to HC, CFS to HC, and QFS to CFS. Volcanoplots showing differential expression of in- and decreased circulating inflammatory proteins when comparing, **a** QFS (n = 31) to HC (n = 50), **b** CFS (n = 50) to HC (n = 50) and **c** QFS (n = 31) tot CFS (n = 50). A total of 92 circulating inflammatory markers were measured using Proximity Extension Essay. Significantly in- and decreased proteins are shown in red and statistical significance was attained if FDR adjusted *P* ≤ 0.05. Coefficients of in- and decreased circulating inflammatory proteins are shown in Table 2. *QFS* Q fever fatigue syndrome, *HC* healthy controls, *CFS* chronic fatigue syndrome

Differential expression of circulating inflammatory proteins is shown in Table 2. In total, there are 5, 27, and 0 proteins identified to be differentially expressed (FDR < 0.05) when comparing QFS to HC, CFS to HC, and QFS to CFS, respectively (Fig. 1 and Additional file 2: Figure S2).

**Table 2:** Differential expression of in- and decreased circulating inflammatory proteins when comparing QFS to HC, CFS to HC, and QFS to CFS

A				
Protein	Coefficient	Std. error	*P* value	FDR
**QFS versus HC**				
Increased				
4E.BP1	1.4844	0.1848	9.60E−16	6.24E−14
CD40	0.5412	0.1025	1.30E−07	4.22E−06
AXIN1	0.9512	0.2358	5.51E−05	8.95E−04
Decreased				
MMP.1	− 1.1973	0.2414	7.09E−07	1.54E−05
ST1A1	− 0.9150	0.2387	1.26E−04	1.64E−03

B				
Protein	Coefficient	Std. error	*P* value	FDR
**CFS versus HC**				
Increased				
X4E.BP1	1.3171	0.2502	1.41E−07	4.54E−06
AXIN1	1.0072	0.2904	0.0005	0.0038
Decreased				
MMP.1	− 1.3036	0.2207	3.51E−09	2.28E-07
LIF.R	− 0.3294	0.0635	2.10E−07	4.54E-06
MCP.1	− 0.3929	0.0896	1.17E−05	0.0002
CCL25	− 0.5710	0.1401	4.58E−05	0.0006
Flt3L	− 0.3485	0.0877	7.03E−05	0.0008
MCP.4	− 0.5425	0.1408	0.0001	0.0011
SCF	− 0.3889	0.1029	0.0002	0.0013
FGF.19	− 0.6764	0.2009	0.0008	0.0047
CCL11	− 0.3501	0.1044	0.0008	0.0047
CXCL11	− 0.5295	0.1625	0.0011	0.0061
DNER	− 0.2270	0.0704	0.0013	0.0063
CXCL9	− 0.5179	0.1622	0.0014	0.0065
LAP.TGF.beta.1	− 0.2959	0.1003	0.0032	0.0138
TRANCE	− 0.4552	0.1557	0.0035	0.0141
CXCL1	− 0.6282	0.2218	0.0046	0.0177
TWEAK	− 0.2665	0.0992	0.0072	0.0256
CX3CL1	− 0.2371	0.0890	0.0077	0.0256
CXCL5	− 0.8374	0.3152	0.0079	0.0256
ST1A1	− 0.5975	0.2266	0.0084	0.0256
MCP.2	− 0.3425	0.1304	0.0086	0.0256
HGF	− 0.2286	0.0882	0.0096	0.0267
OSM	− 0.4414	0.1710	0.0098	0.0267
IL7	− 0.3355	0.1389	0.0157	0.0392
CXCL6	− 0.4310	0.1784	0.0157	0.0392
CST5	− 0.2759	0.1176	0.0190	0.0456

C				
Protein	Coefficient	Std. error	*P* value	FDR
**QFS versus CFS**				
Increased				
IL8	0.3432	0.1319	0.0093	0.1945
CD244	0.2973	0.1112	0.0075	0.1945
CXCL11	0.5682	0.2261	0.0120	0.1945
CCL11	0.3821	0.1330	0.0041	0.1945
LIF.R	0.1770	0.0787	0.0245	0.2355
CD40	0.3009	0.1378	0.0290	0.2355
CCL25	0.3994	0.1819	0.0281	0.2355
CD8A	0.3177	0.1501	0.0343	0.2477
IL.10RB	0.2368	0.1191	0.0467	0.2683
Decreased				
ST1A1	− 0.6707	0.2828	0.0177	0.2303

In- and decreased inflammatory proteins when comparing (A) QFS (n = 31) to HC (n = 50), (B) CFS (n = 50) to HC (n = 50), and (C) QFS (n = 31) to CFS (n = 50). Results are depicted as a coefficient with Std. Error and FDR. Only proteins that are significantly in- or decreased are depicted and statistical significance was attained if *P* ≤ 0.05

*QFS* Q fever fatigue syndrome, *HC* healthy controls, *CFS* chronic fatigue syndrome, *Std. error* standard error, *FDR* false discovery rate

### Inflammatory proteomics-based models can discriminate QFS, CFS and HC

Additional file 1: Figure S1 depicts the varying prediction performance of the model with different partition of data by cross validation. The median of training and prediction performance in QFS versus HC and CFS versus HC is close to 1, while the median of training and prediction performance in QFS versus CFS is lower. Based on the large difference of protein expression levels, it is relatively easier to discriminate QFS and CFS from HC. The following variables proved most important in prediction when comparing QFS with HC; 4E-BP1, CD40, AXIN1, CCL11, CD244, IL-8, OPG, CCL4, TRAIL, and CD8A, CFS with HC; 4E-BP1, CDCP1, AXIN1, MMP-10, CSF-1, TNFB, NT-3, FGF-23, IL-12B, and IL-8, and QFS and CFS with HC; 4E-BP1, AXIN1, CD40, CDCP1, CSF-1, IL-8, FGF-23, CCL4, ADA, and MMP-10 (Additional file 3: Figure S3).

### Differential association patterns between inflammatory protein and metabolites in disease and health

**Fig. 2:**
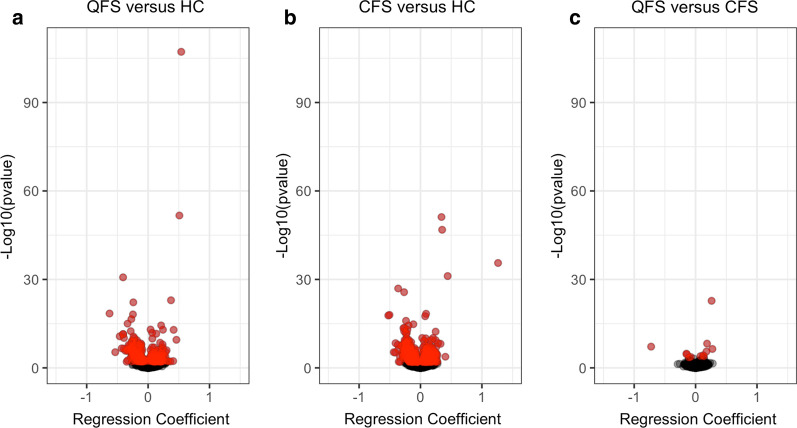
Significantly in- and decreased circulating metabolites when comparing QFS to HC, CFS to HC, and QFS to CFS. Volcanoplots showing in- and decreased circulating metabolites when comparing, **a** QFS (n = 31) to HC (n = 22), **b** CFS (n = 50) to HC (n = 22) and **c** QFS (n = 31) tot CFS (n = 50). A total of 1607 metabolic features were assessed with a high-throughput non-targeted metabolomics approach. Significantly in- and decreased metabolites are shown in red and statistical significance was attained if FDR adjusted *P* ≤ 0.05. Coefficients of in- and decreased circulating inflammatory proteins are shown in Additional file 4: Table S1. *QFS* Q fever fatigue syndrome, *HC* healthy controls, *CFS* chronic fatigue syndrome

There are 319, 441, and 12 significantly in- and decreased metabolites when comparing QFS patients to HC, CFS patients to HC, and QFS patients to CFS patients, respectively (FDR < 0.05, Fig. 2, Additional file 2: Figure S2, and Additional file 4: Table S1). When comparing QFS to HC, the identified metabolites are enriched in primary bile acid biosynthesis (*P* = 0.0116), sphingolipid metabolism (*P* = 0.0256), nitrogen metabolism (*P* = 0.0394), and d-glutamine and d-glutamate metabolism (*P* = 0.0394) pathways. When comparing CFS to HC, the sphingolipid metabolism (*P* = 0.0033) pathway is enriched. When comparing QFS patients to CFS patients, the nitrogen (*P* = 0.0154), d-glutamine and d-glutamate metabolism (*P* = 0.0154), arginine (*P* = 0.0357), butanoate (*P* = 0.387), and histidine metabolism (*P* = 0.0407) are enriched.

**Fig. 3:**
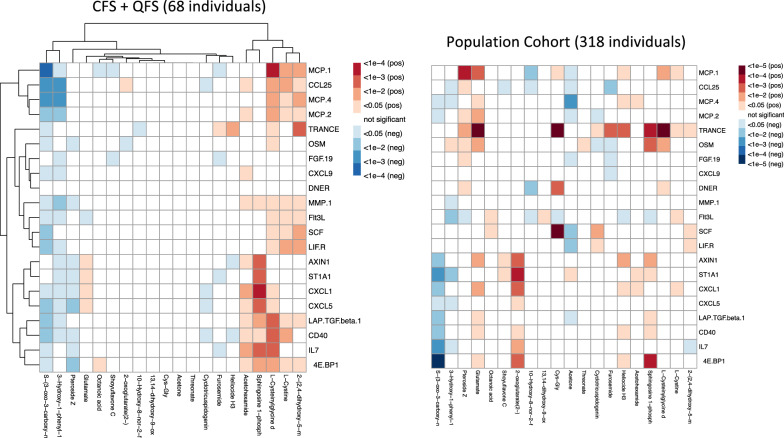
Global correlation pattern of chronically fatigued patients compared to HC. Global correlation pattern by means of average clustering, using the most significantly different proteins and metabolites (FDR adjusted *P* ≤ 0.05) in both QFS and CFS, used as a reference for a large HC group of 318 individuals (www.humanfunctionalgenomics.org). The global correlation pattern exposes acetohexamide, sphingosine 1-phosphate, l-cysteinylglycine, l-cysteine, and 2-(2,4-dihydroxy-5-m as metabolites of particular interest as they positively correlate with inflammatory proteins. *HC* healthy controls, *QFS* Q fever fatigue syndrome, *CFS* chronic fatigue syndrome

**Fig. 4:**
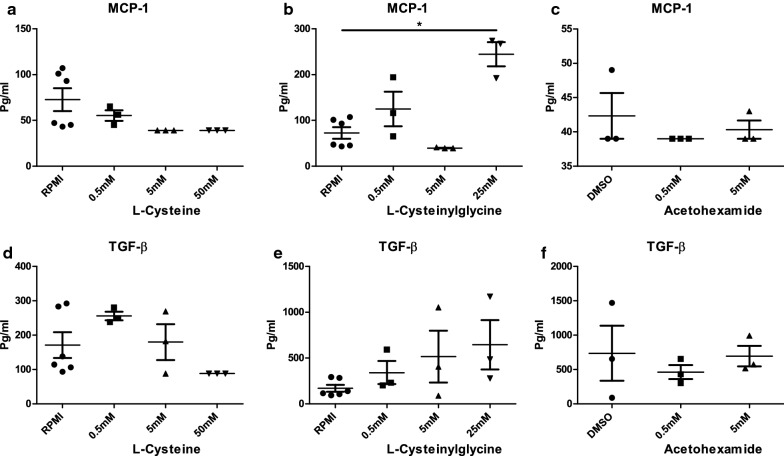
Production of MCP-1 and TGF-β after 24 h incubation of peripheral blood mononuclear cells (PBMCs) of HC with l-cysteine, l-cysteinylglycine, and Acetohexamide. PBMCs were stimulated with various concentrations of l-cysteine (**a**, **d**), l-cysteinylglycine (**b**, **e**), and acetohexamide (**c**, **f**), together with a negative control, i.e. RPMI and DMSO, for 24 h, after which concentrations of MCP-1 (**a**–**c**) and TGF-β (**d**–**f**) were measured in the supernatants with ELISA. **b** Shows that stimulation with 25 mM l-cysteinylglycine resulted in a significantly higher MCP-1 production compared to RPMI as a negative control (*P* = 0.0238). No significant differences were when stimulating with l-cysteinylglycine, l-cysteine, or acetohexamide (**a**, **c**–**f**). Data were analysed with the Mann–Whitney test and are depicted as mean ± SEM. Statistical significance was attained if *P* ≤ 0.05. *HC* healthy controls, *MCP* Monocyte Chemoattractant Protein, *TGF* tumor growth factor, *RPMI* Roswell Park Memorial Institute culture medium, *DMSO* dimethylsulfoxide, *ELISA* enzyme-linked immuno assay, *SEM* standard error of mean. *P ≤ 0.05

Next, we investigated in which way the inflammatory proteins are associated with the metabolites in patients and healthy individuals, respectively. We illustrate the correlation between the differentially expressed proteins (FDR < 0.05) and the top 20 differentially expressed metabolites (with similar number of proteins) in QFS + CFS patients versus HC. This clustering pattern was then used as a reference for the same type of data from a population-based cohort of 318 individuals (www.humanfunctionalgenomics.org) (Fig. 3). As shown in Fig. 3, metabolites acetohexamide, sphingosine l-phosphate, l-cysteinylglycine, l-cysteine, and 2-(2,4-dihydroxy-5-m are of particular interest as they positively correlate with inflammatory proteins. Validation experiments with PBMCs of HC showed that stimulation with 25 mM l-cysteinylglycine resulted in a significantly higher MCP-1 production compared to RPMI as a negative control (Mann–Whitney U test, *P* = 0.0238). No significant differences were observed for TGF-β, or MCP-1 when stimulating with lower concentrations, i.e., 0.5 mM and 5 mM, of l-cysteinylglycine, l-cysteine, or acetohexamide (Fig. 4).

### QFS and CFS show a microbiome composition distinct from HC

**Fig. 5:**
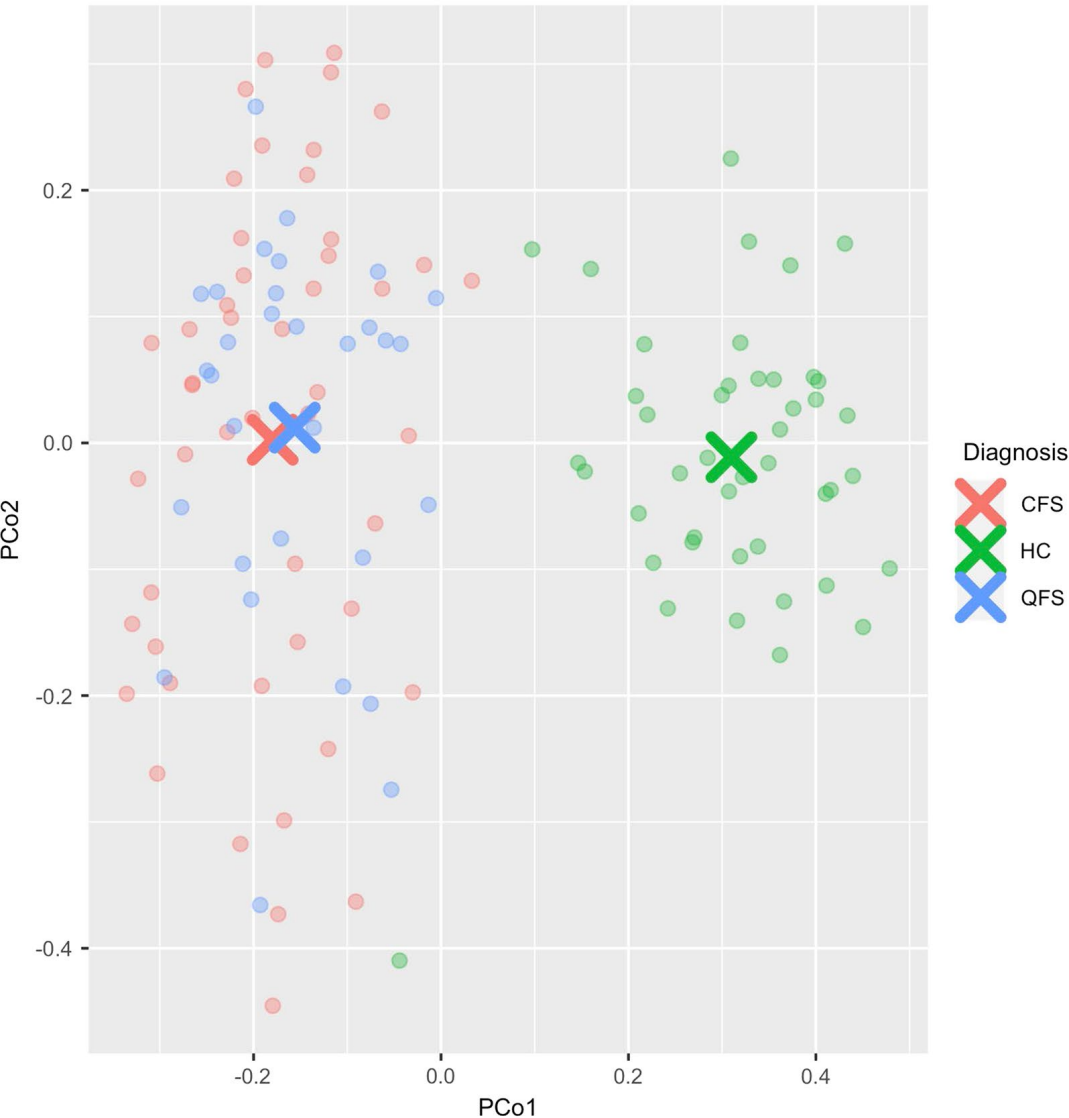
Principal coordinates analysis (PCoA) of gut microbiome taxonomy of CFS, QFS, and HC. A PCoA analysis of gut microbiome taxonomy, assessed by Metagenomic sequencing using the Illumina HiSeq platform, of QFS (n = 31), CFS (n = 50), and HC (n = 50), shows a clear distinction of both QFS and CFS patients from HC. *PCoA* Principal coordinates analysis, *CFS* chronic fatigue syndrome, *QFS* Q fever fatigue syndrome, *HC* healthy controls

A PCoA on gut microbiome taxonomy of QFS, CFS, and HC was performed, showing a clear-cut difference between QFS and CFS, and HC (Fig. 5). There are 36, 44, and 2 features showing significant differences in gut microbiome taxonomy when comparing QFS to HC, CFS to HC, and QFS to CFS, respectively (Additional file 2: Figure S2, Additional file 5: Figure S4, and Additional file 6: Table S2). When comparing QFS patients to HC there is an increase in abundance of *Bacteroidetes* with *Bacteroides* and *Alistiples* spp., and a decrease in abundance of *Firmicutes* and *Actinobacteria* with *Ruminococcus* and *Bifidobacterium* spp., respectively. When comparing CFS patients to HC, we find an increase in abundance of *Firmicutes* and *Actinobacteria* with *Ruminococcus* and *Bifidobacterium* spp., respectively, and a decrease in abundance of Bacteroidetes with *Alistiples* and *Bacteroides* spp. When comparing QFS patients to CFS patients, we find a slight increase in abundance of *Firmicutes* with *Eubacterium* and *Faecalibacterium* spp. in the former. Additional file 7: Table S3 depicts significantly in- and decreased gut microbiome functional pathways when comparing QFS to HC, CFS to HC, and QFS to CFS.

Finally, we investigated in which way the gut microbiome is associated with metabolites in fatigued patients, i.e., QFS and CFS, as HC hardly show any overlap. Only two significant correlations were found; Bifidobacterium_adolescentis and *N*-docosahexaenoyl GABA, and Subdoligranulum_unclassified and Arbekacin (Additional file 8: Figure S5).

## Discussion

This study showed that inflammatory and metabolomic profiles, together with gut microbiome taxonomy, of QFS and CFS patients are quite similar, and both groups clearly differ from HC (with CFS patients showing a larger difference than QFS patients). These findings are important, as they indicate that QFS and CFS patients show a common denominator in the long term, i.e., alterations in inflammatory and metabolomic profiles, together with gut microbiome taxonomy, regardless of the precipitating event that started the complaints.

Although important characteristics such as blood inflammatory profile, gut microbiome, and blood metabolome are very similar in QFS and CFS, subtle differences are still observed. It was previously shown that QFS patients tend to exhibit more of an inflammatory profile than CFS patients [5, 12]. A similar trend is observed in our study. One could speculate that the microbial origin of QFS plays a role in this subtle persistent inflammation. Together with previous findings on differences in fatigue-perpetuating factors and response to cognitive behavioural therapy (CBT) [42–44], one could advocate that QFS should be seen as a separate, more inflammatory, fatigue syndrome entity that requires a different diagnostic [27, 28] and therapeutic [44, 45] approach. These findings argue for a ‘splitting’ rather than a ‘lumping’ approach to chronic fatigue [46].

Inflammatory markers 4E-BP1, AXIN1, and MMP-1 showed the potential to differentiate both QFS and CFS patients from HC and might therefore be associated with fatigue in general as this is the common denominator between these groups. We further elaborated on these findings by using a machine-learning approach showing that both 4E-BP1 and AXIN1 are good candidate biomarkers for predicting/diagnosing chronic fatigue. The eukaryotic translation initiation factor 4E binding protein 1 (4E-BP1) represses mRNA translation downstream of the mammalian target of rapamycin (mTOR). The latter is known to phosphorylate and inactivate 4E-BP1 [47]. Several upstream stimuli, e.g., growth factors and cytokines, can regulate downstream processes, e.g., cell growth, cell proliferation, and cell plasticity, through mTOR [47]. Dennis et al. [48] showed that the 4E-BP1 phosphorylation was inhibited when intracellular adenosine triphosphate (ATP) levels were lowered. Interestingly, chronic fatigue has previously been associated with a decrease in cell metabolism [15, 18, 49, 50], and PBMCs of CFS patients showed a decrease in mitochondrial function compared to PBMCs of HC when stressed [51–53]. Axis inhibition protein (AXIN1), negatively regulates the Wnt signalling pathway by downregulation of β-catenin [54], but has also been identified as a scaffold protein that activates TGF-β signalling [55]. Especially the latter finding is of interest as elevated levels of TGF-β have frequently been associated with CFS [10]. However, it should be noted that results on TGF-β levels must be interpreted with great caution as measuring TGF-β in plasma has some noteworthy, pre-analytic, pitfalls [56]. Matrix metalloproteinase 1 (MMP-1) is a collagen cleaving protease that has been associated with inflammation in infections such as HIV [57, 58], but has also shown to have a negative association with the risk of being a CFS patient [59]. Exactly how, and how strong, 4E-BP1, AXIN1, and MMP-1 relate to chronic fatigue warrants further investigation in independent cohorts.

Comparing CFS patients to HC, studies on metabolomic profiles consistently found differences between these groups [17–20]. Armstrong et al. [20] found that CFS patients show lower levels of glutamine and ornithine compared to HC. Germain et al. [19] found pathway abnormalities in taurine, glycerophospholipid, primary bile acid, glyoxylate, dicarboxylate, and fatty acid metabolism. Naviaux et al. [18] suggested that CFS patients exhibit a hypometabolic state and found pathway abnormalities in sphingolipid, phospholipid, purine, cholesterol, microbiome, pyrroline-5-carboxylate, riboflavin, branch chain amino acid, peroxisomal, and mitochondrial metabolism. Our study shows enrichment similarities in sphingolipid and primary bile acid biosynthesis pathways. As the sphingolipid pathway is altered in both QFS and CFS, these pathway alterations might be specific for chronic fatigue in general, whereas the primary bile acid biosynthesis pathway might be more specific for QFS.

Additionally, several of these metabolites, e.g., l-cysteine and l-cysteinylglycine, appear to positively correlate with various inflammatory proteins, e.g., MCP-1, but also 4E-BP1 and MMP-1. PBMCs stimulated with l-cysteinylglycine produced significantly more MCP-1 compared to PBMCs that are stimulated with the negative control RPMI. A similar trend was observed for l-cysteine. This shows us that some of these metabolites might have the potential to initiate a more (anti-)inflammatory environment. One could speculate that such a mechanism contributes to changes in inflammation in QFS and CFS patients, and that the observed inflammation is secondary to metabolic alterations. Further investigation and validation of these results is warranted, with additional cytokines and chemokines, e.g., 4E-BP1, AXIN1, and MMP-1, in which the metabolite sphingosine 1‐phosphate is of particular interest as it is part of the sphingolipid pathway (enriched in both QFS and CFS compared to HC). Furthermore, as our group recently showed that monocytes of QFS and CFS patients exhibit a decreased expression of MDP-coding genes *MT-RNR1* and *MT-RNR2* compared to HC [15], it would also be interesting to investigate the role of these MDP-coding peptides in these metabolic and inflammatory alterations.

Previous studies on gut microbiome composition compared CFS patients to HC and found differences between these groups. Unfortunately, many of the differences are inconsistent. Giloteaux et al. [22] showed that the gut microbiome of CFS patients has less bacterial diversity with the balance shifting towards more pro-inflammatory species. Sheedy et al. [60] showed that CFS patients have more aerobic microbial flora, with more Gram-positives, and an abundance of *E. faecalis* and *S. sanguinis* compared to HC. Armstrong et al. [61] found an increase in *Clostridium* spp. and a decrease in total bacteria, total anaerobic bacteria, and *Bacteroides spp*. In CFS patients compared to HC. Fremont et al. [62] found that both Belgian and Norwegian CFS patients had an increase in *Lactinofacter* compared to HC. Shukla et al. [63] found a decreased mean relative abundance of *Actinobacteria* in CFS patients compared to HC. Our study found a similar decrease in *Bacteroides* spp. when comparing CFS patients to HC. Interestingly, this genus appears to be increased when comparing QFS patients to HC. Furthermore, we conflictingly find an increase in *Actinobacteria* when comparing CFS patients, but a decrease when comparing QFS patients, to HC. Our most important observation, however, is that the taxonomy of QFS and CFS patients is quite similar, while both groups appear to differ quite profoundly from HC (with CFS patients showing a larger difference than QFS patients). This is similar to our findings in inflammatory and metabolomic profiles and functionally reflected by highly significant upregulation of pathways, like urate biosynthesis/inosine 5′-phosphate degradation and CMP-3-deoxy-d-manno-octulosonate biosynthesis, when comparing QFS and CFS patients to HC. When one compares QFS patients to CFS patients, less significant upregulation of pathways, like l-lysine biosynthesis III and VI, is found. Exactly how gut microbiome dysbiosis plays part in the pathophysiology of chronic fatigue remains unclear but likely involve the microbiome-brain-axis, and/or subsequent systemic low-grade inflammation. A recent systematic review confirmed that even though independent studies do report differences, these differences are inconsistent [23]. Such inconsistencies are likely to occur if control groups are not representative and/or in- and exclusion criteria for patients are not strictly adhered to. Further investigation of the gut microbiome, using strict in- and exclusion criteria together with adequate and representative control groups [64], in patients with chronic fatigue is definitely of interest.

Although our study lacks a replication cohort, the observed differential patterns among QFS, CFS and HC are consistent across three omics layers. A batch effect across different (control) groups is unlikely, but should be kept in mind when interpreting these data. Because systematic assessment of multi-omics data is still limited, our detailed datasets are an important reference for improving our understanding of the molecular processes leading to a state of chronic fatigue.

## Conclusion

In conclusion, this study shows that QFS and CFS patients are similar based on their inflammatory and metabolomic profiles, together with gut microbiome taxonomy, while both QFS and CFS patients differ from HC (with CFS patients showing a larger difference than QFS patients). These data suggest that QFS and CFS are similar across three omics layers, indicating cross validation. Furthermore, correlation between metabolomic and proteomic data was validated with laboratory experiments, and a prediction analysis was performed on proteomic data, exposing 4E-BP1 and MMP-1 as potential biomarkers for chronic fatigue. However, while similarities between QFS and CFS are seen and could be associated with chronic fatigue in general, subtle differences, e.g., in inflammatory profiles, should be considered when further investigating its pathogenic mechanisms.

## Supplementary information

**Additional file 1: Figure S1.** Boxplots showing AUC of training and prediction performances when comparing QFS to HC, CFS to HC and QFS to CFS. Boxplots showing AUC of training and prediction performances when comparing (A) QFS to HC, (B) CFS to HC, and (C) QFS to CFS. Repeated Cross validation (CV) approach was used for building prediction models. The procedure was repeated 1000 times, and the AUC was calculated to evaluate the predictive power of the model. The median of training and prediction performance in QFS versus HC (A) and CFS versus HC (B) is close to 1, while the median of training and prediction performance in QFS versus CFS is lower (C). *QFS* Q fever fatigue syndrome, *HC* healthy controls, *CFS* chronic fatigue syndrome, *AUC* area under the curve, *CV* cross validation.

**Additional file 2: Figure S2.** Overlap of in- and decreased circulating inflammatory proteins, circulating metabolites, and taxonomic differences in gut microbiome composition when comparing QFS to HC, CFS to HC, and QFS to CFS. Venn diagrams showing overlap in in- and decreased circulating inflammatory proteins (A), circulating metabolites (B), and taxonomic differences in gut microbiome composition (C) when comparing QFS to HC, CFS to HC, and QFS to CFS. Venn diagrams were made at https://jvenn.toulouse.inra.fr/app/example.html [65]. *QFS* Q fever fatigue syndrome, *CFS* chronic fatigue syndrome.

**Additional file 3: Figure S3.** Frequency of selection of 65 Proteins by Least Absolute Shrinkage and Selection Operator (LASSO) with 1000-time repeated cross-validation when comparing QFS to HC, CFS to HC, and QFS to CFS. Graphs showing frequency of selection by Least Absolute Shrinkage and Selection Operator (LASSO) of circulating inflammatory markers when comparing (A) QFS to HC; 4E-BP1, CD40, AXIN1, CCL11, CD244, IL-8, OPG, CCL4, TRAIL, and CD8A, (B) CFS to HC; 4E-BP1, CDCP1, AXIN1, MMP-10, CSF-1, TNFB, NT-3, FGF-23, IL-12B, and IL-8, and (C) QFS and CFS to HC; 4E-BP1, AXIN1, CD40, CDCP1, CSF-1, IL-8, FGF-23, CCL4, ADA, and MMP-10. *QFS* Q fever fatigue syndrome, *HC* healthy controls, *CFS* chronic fatigue syndrome.

**Additional file 4: Table S1.** Significantly in- and decreased metabolites when comparing QFS to HC, CFS to HC, and QFS to CFS. In- and decreased metabolites when comparing (A) QFS (n = 31) to HC (n = 50), (B) CFS (n = 50) to HC (n = 50), and (C) QFS (n = 31) to CFS (n = 50). Results are depicted as a coefficient with Std. Error and FDR. Statistical significance was attained if *P* ≤ 0.01. *QFS* Q fever fatigue syndrome, *HC* healthy controls, *CFS* chronic fatigue syndrome, *Std. Error* standard error, *FDR* False Discovery Rate.

**Additional file 5: Figure S4.** Taxonomic differences in gut microbiome composition when comparing QFS to HC, CFS to HC, and QFS to CFS. Volcanoplots showing differences in gut microbiome taxonomy when comparing (A) QFS (n = 31) to HC (n = 50), (B) CFS (n = 50) to HC (n = 50) and (C) QFS (n = 31) tot CFS (n = 50). The gut microbiome composition was assessed by Metagenomic sequencing using the Illumina HiSeq platform Significantly in- and decreased microbes are shown in red and statistical significance was attained if FDR adjusted *P* ≤ 0.05._Log2FoldChange_ of Significantly in- and decreased microbes are shown in Additional file 6: Table S2. *QFS* Q fever fatigue syndrome, *HC* healthy controls, *CFS* chronic fatigue syndrome.

**Additional file 6: Table S2.** Gut microbiome taxonomic differences when comparing QFS to HC, CFS to HC, and QFS to CFS. Gut microbiome taxonomic differences when comparing (A) QFS (n = 31) to HC (n = 50), (B) CFS (n = 50) to HC (n = 50), and (C) QFS (n = 31) to CFS (n = 50). Results are depicted as Log2FoldChange and significance was attained if adjusted *P* ≤ 0.05. *QFS* Q fever fatigue syndrome, *HC* healthy controls, *CFS* chronic fatigue syndrome. Statistical significance was attained if adjusted *P* ≤ 0.05.

**Additional file 7: Table S3.** Gut microbiome functional differences when comparing QFS to HC, CFS to HC, and QFS to CFS. Gut microbiome functional differences when comparing (A) QFS (n = 31) to HC (n = 50), (B) CFS (n = 50) to HC (n = 50), and (C) QFS (n = 31) to CFS (n = 50). Results are depicted as Log2FoldChange and significance was attained if adjusted *P* ≤ 0.05. *QFS* Q fever fatigue syndrome, *HC* healthy controls, *CFS* chronic fatigue syndrome.

**Additional file 8: Figure S5.** Global correlation pattern of chronically fatigued patients. Global correlation pattern by means of average clustering showing significantly different correlations between gut microbiome and metabolites (FDR adjusted *P* ≤ 0.05) in chronically fatigued patients, i.e., QFS and CFS. The global correlation pattern exposes a correlation between Bifidobacterium_adolescentis and *N*-docosahexaenoyl GABA, and Subdoligranulum_unclassified and Arbekacin. *QFS* Q fever fatigue syndrome, *CFS* chronic fatigue syndrome.

## Data Availability

The datasets used and/or analysed during the current study are available from the corresponding author on reasonable request.

## Abbreviations

QFS: Q fever fatigue syndrome
*C. burnetii*: *Coxiella burnetii*
CFS: Chronic fatigue syndrome
PBMCs: Peripheral blood mononuclear cells
TNF: Tumor necrosis factor
IL: Interleukin
IFN: Interferon
MDP: Mitochondrial-derived peptide
HC: Healthy controls
CIS: Checklist Individual Strength (questionnaire)
SIP-8: Sickness Impact Profile-8 (questionnaire)
ECCF: Expert Center for Chronic Fatigue
CDC: Centers for Disease Control
TOF-M: Time-of-flight mass
RPMI: Roswell Park Memorial Institute
ELISA: Enzyme-linked immune sorbent assay
LASSO: Least Absolute Shrinkage and Selection Operator
CV: Cross validation
AUC: Area under the curve
PCoA: Principal coordinates analysis
FDR: False discovery rate
CBT: Cognitive behavioural therapy
4E-BP: 4E binding protein
mTOR: Mammalian target of rapamycin
AXIN: Axis inhibition protein
MMP: Matrix metalloproteinase
SD: Standard deviation
Std. error: Standard error
MCP: Monocyte Chemoattractant Protein
TGF: Tumor growth factor
DMSO: Dimethylsulfoxide
SEM: Standard error of mean
