# Supplementary figures and images for: Multi-omics examination of Q fever fatigue syndrome identifies similarities with chronic fatigue syndrome
Source: J Transl Med. 2020 Nov 26;18:448. doi: 10.1186/s12967-020-02585-5 (PMC7690002; doi:10.1186/s12967-020-02585-5)

A

QFS versus HC

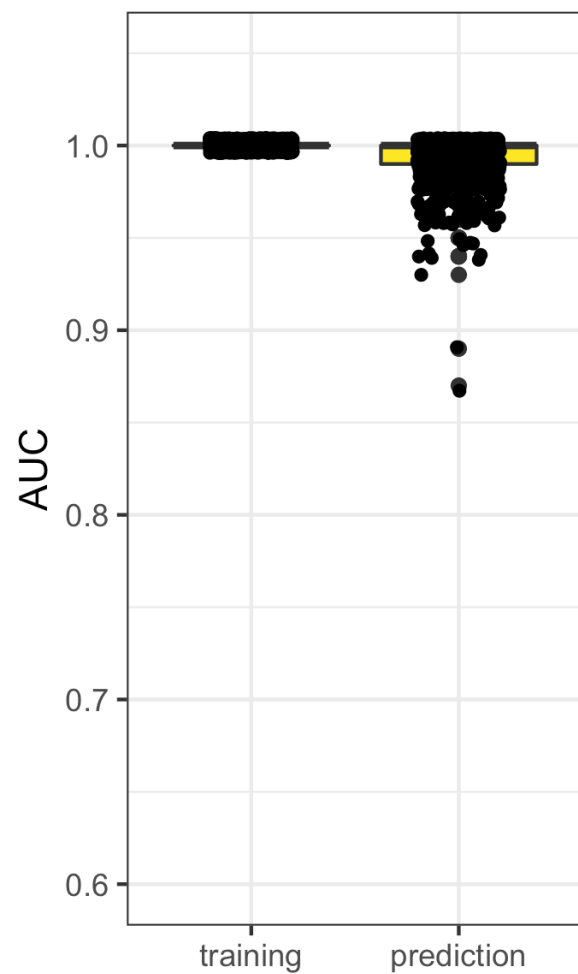

B

CFS versus HC

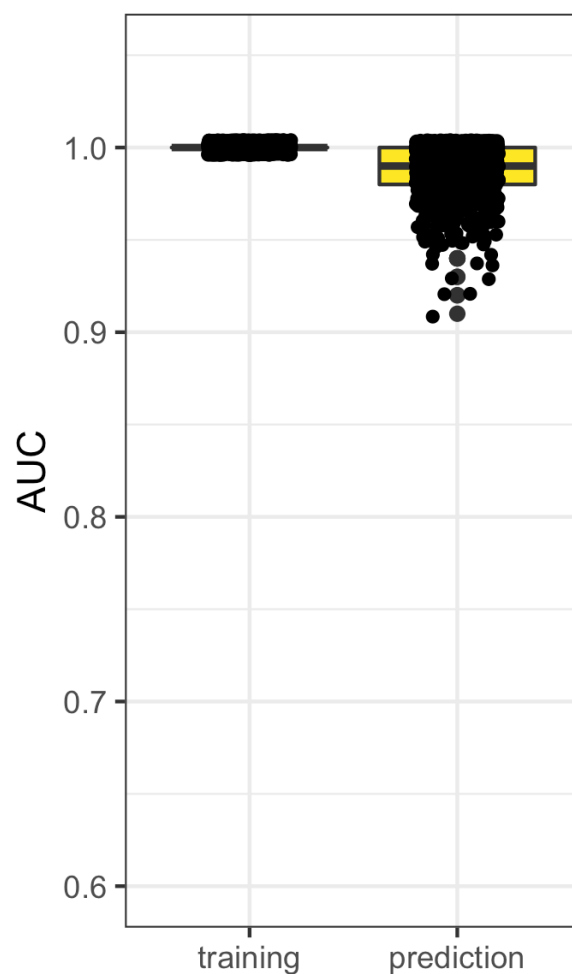

C

QFS versus CFS

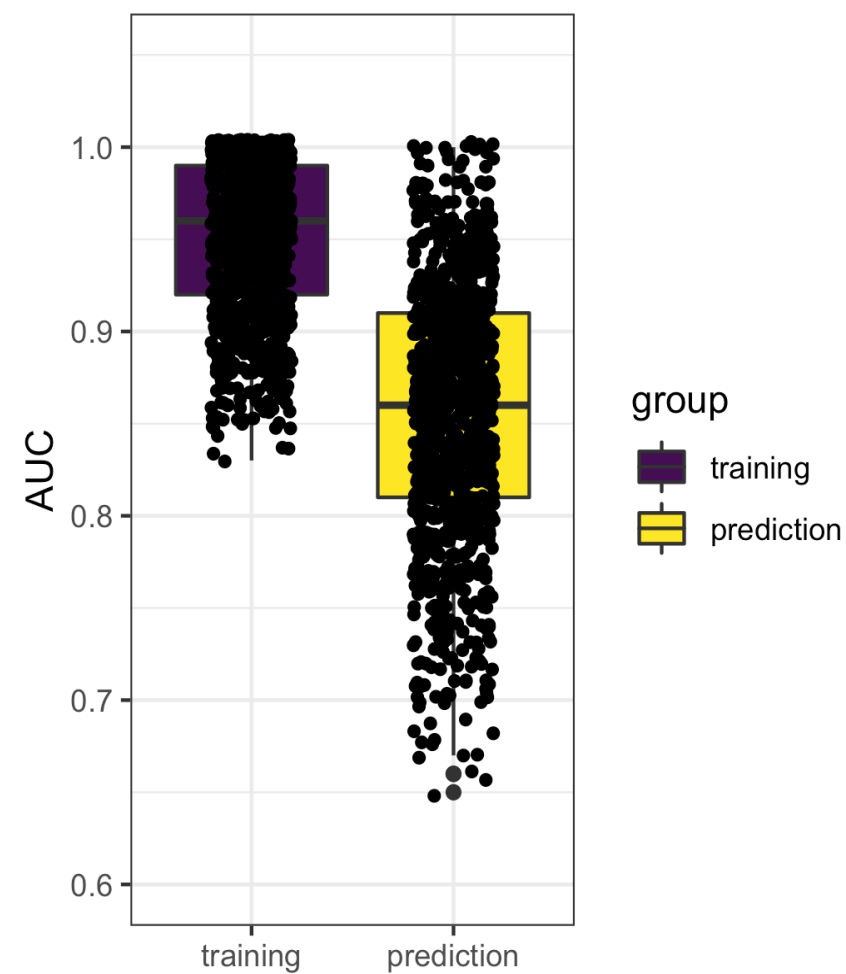

Supplement: Supplementary file 1 — Additional file 1: Figure S1. Boxplots showing AUC of training and prediction performances when comparing QFS to HC, CFS to HC and QFS to CFS. Boxplots showing AUC of training and prediction performances when comparing (A) QFS to HC, (B) CFS to HC, and (C) QFS to CFS. Repeated Cross validation (CV) approach was used for building prediction models. The procedure was repeated 1000 times, and the AUC was calculated to evaluate the predictive power of the model. The median of training and prediction performance in QFS versus HC (A) and CFS versus HC (B) is close to 1, while the median of training and prediction performance in QFS versus CFS is lower (C). QFS Q fever fatigue syndrome, HC healthy controls, CFS chronic fatigue syndrome, AUC area under the curve, CV cross validation. [file 12967_2020_2585_MOESM1_ESM.pdf]

Positive

Negative

A

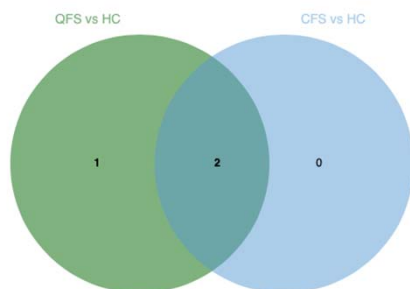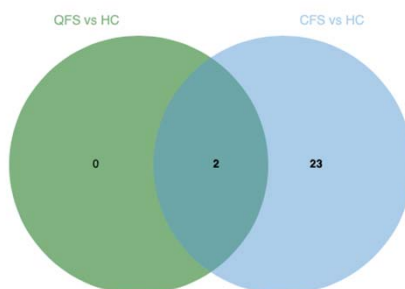

B

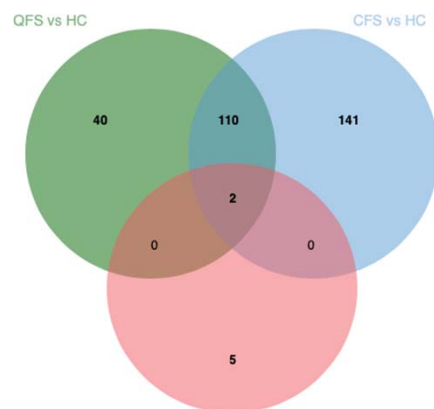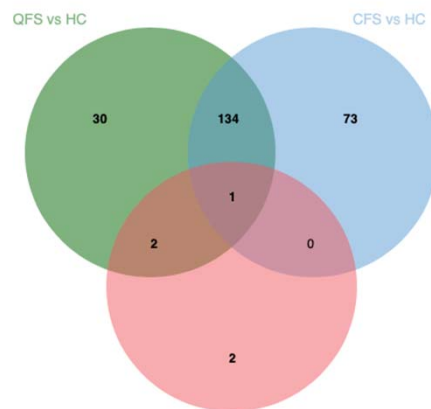

C

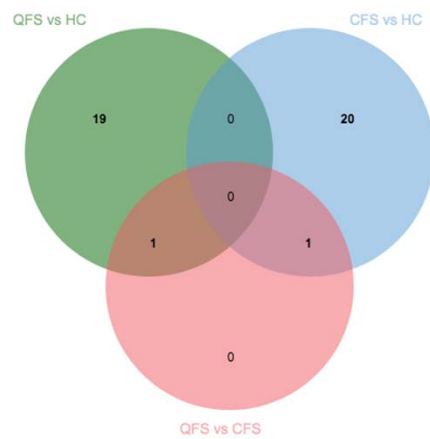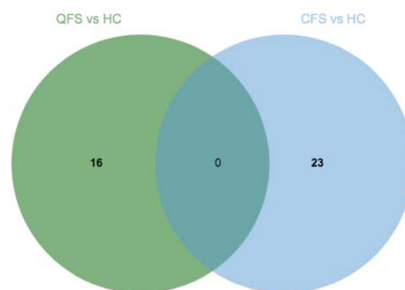

Supplement: Supplementary file 2 — Additional file 2: Figure S2. Overlap of in- and decreased circulating inflammatory proteins, circulating metabolites, and taxonomic differences in gut microbiome composition when comparing QFS to HC, CFS to HC, and QFS to CFS. Venn diagrams showing overlap in in- and decreased circulating inflammatory proteins (A), circulating metabolites (B), and taxonomic differences in gut microbiome composition (C) when comparing QFS to HC, CFS to HC, and QFS to CFS. Venn diagrams were made at https://jvenn.toulouse.inra.fr/app/example.html [65]. QFS Q fever fatigue syndrome, CFS chronic fatigue syndrome. [file 12967_2020_2585_MOESM2_ESM.pdf]

**A**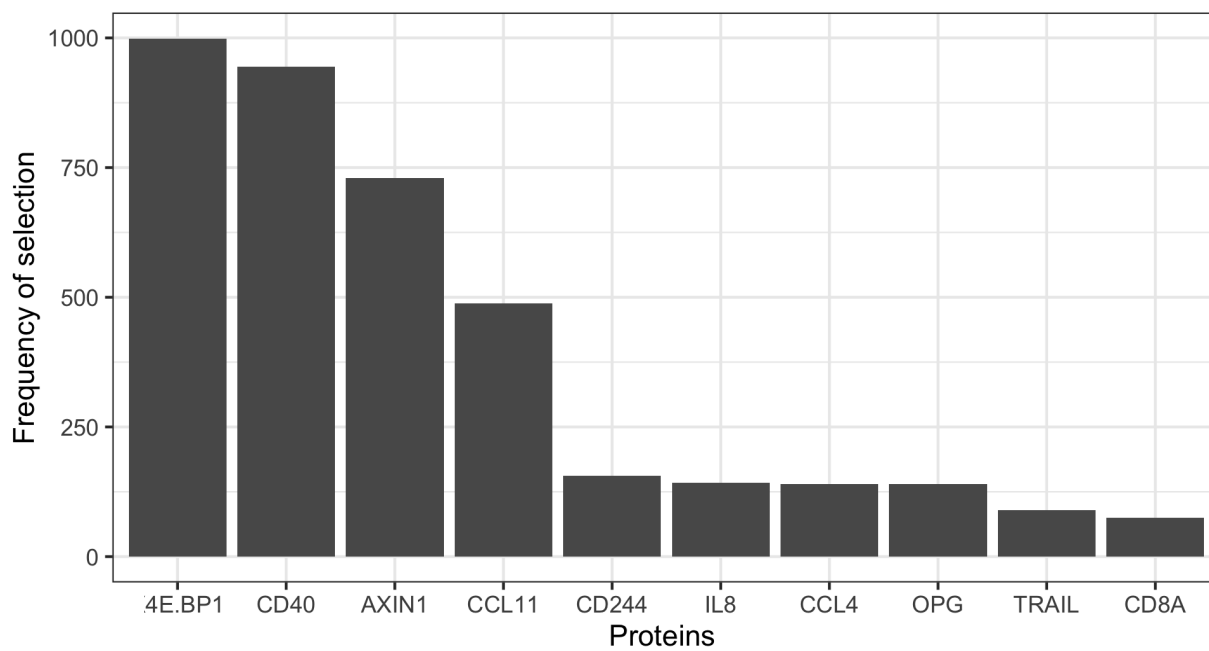**B**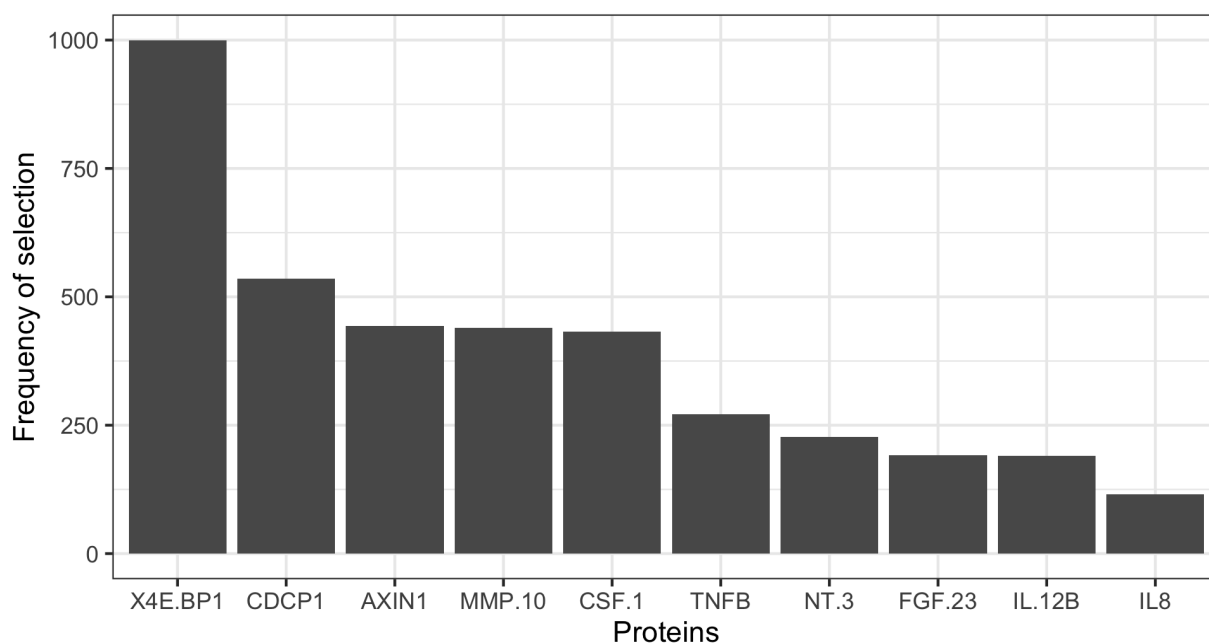**C**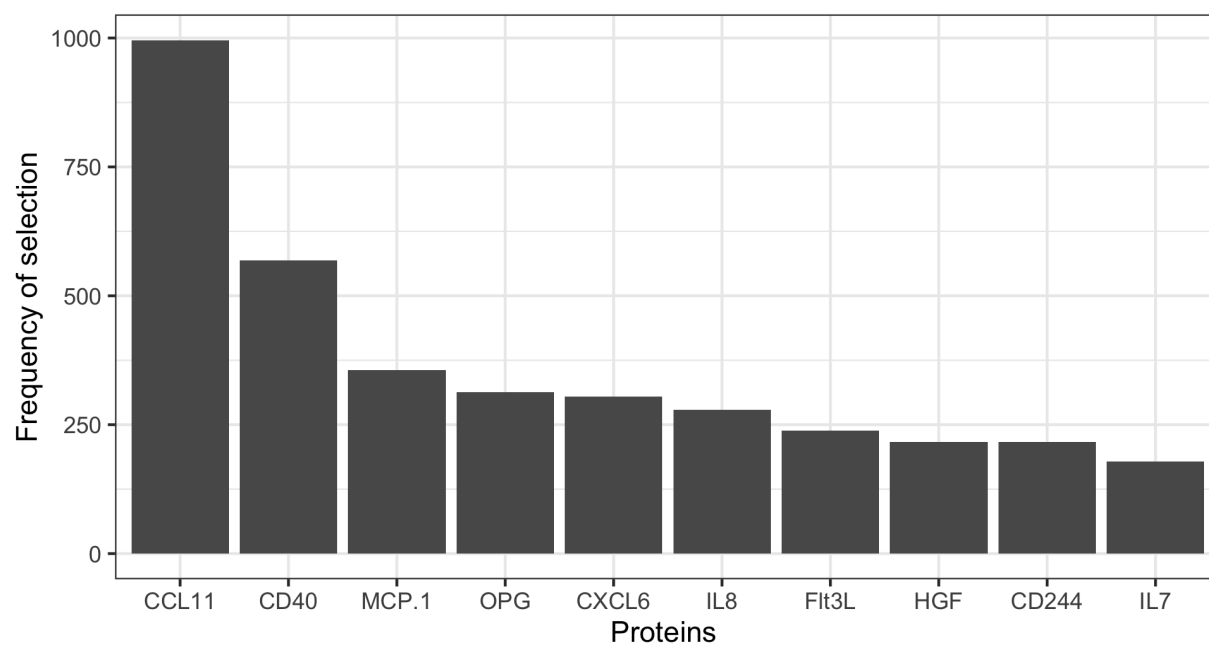

Supplement: Supplementary file 3 — Additional file 3: Figure S3. Frequency of selection of 65 Proteins by Least Absolute Shrinkage and Selection Operator (LASSO) with 1000-time repeated cross-validation when comparing QFS to HC, CFS to HC, and QFS to CFS. Graphs showing frequency of selection by Least Absolute Shrinkage and Selection Operator (LASSO) of circulating inflammatory markers when comparing (A) QFS to HC; 4E-BP1, CD40, AXIN1, CCL11, CD244, IL-8, OPG, CCL4, TRAIL, and CD8A, (B) CFS to HC; 4E-BP1, CDCP1, AXIN1, MMP-10, CSF-1, TNFB, NT-3, FGF-23, IL-12B, and IL-8, and (C) QFS and CFS to HC; 4E-BP1, AXIN1, CD40, CDCP1, CSF-1, IL-8, FGF-23, CCL4, ADA, and MMP-10. QFS Q fever fatigue syndrome, HC healthy controls, CFS chronic fatigue syndrome. [file 12967_2020_2585_MOESM3_ESM.pdf]
