# Supplementary material for: Multi-omics examination of Q fever fatigue syndrome identifies similarities with chronic fatigue syndrome
Source: J Transl Med. 2020 Nov 26;18:448. doi: 10.1186/s12967-020-02585-5 (PMC7690002; doi:10.1186/s12967-020-02585-5)
Supplement: Supplementary file 4 — Additional file 4: Table S1. Significantly in- and decreased metabolites when comparing QFS to HC, CFS to HC, and QFS to CFS. In- and decreased metabolites when comparing (A) QFS (n = 31) to HC (n = 50), (B) CFS (n = 50) to HC (n = 50), and (C) QFS (n = 31) to CFS (n = 50). Results are depicted as a coefficient with Std. Error and FDR. Statistical significance was attained if P ≤ 0.01. QFS Q fever fatigue syndrome, HC healthy controls, CFS chronic fatigue syndrome, Std. Error standard error, FDR False Discovery Rate. [file 12967_2020_2585_MOESM4_ESM.docx]

**Table S1. Significantly in- and decreased metabolites when comparing QFS to HC, CFS to HC, and QFS to CFS.**

**A.**

| QFS versus HC | | | | | |
| --- | --- | --- | --- | --- | --- |
| Ion Index | Compound | Coefficient | Std. Error | *P* Value | FDR |
| 648 | L-Cysteinylglycine disulfide | 0.5395 | 0.0245 | 6.61E-108 | 1.06E-104 |
| 438 | L-Cystine | 0.5092 | 0.0334 | 2.11E-52 | 1.70E-49 |
| 102 | Octanoic acid | 0.3731 | 0.0372 | 1.17E-23 | 4.69E-21 |
| 201 | Cys-Gly | 0.2121 | 0.0270 | 3.89E-15 | 6.25E-13 |
| 99 | 2-oxoglutarate(2-) | 0.0399 | 0.0054 | 1.02E-13 | 1.49E-11 |
| 1 | Acetone | 0.2425 | 0.0327 | 1.20E-13 | 1.60E-11 |
| 54 | Cys | 0.4161 | 0.0562 | 1.30E-13 | 1.60E-11 |
| 1082 | Shoyuflavone C | 0.0651 | 0.0092 | 1.30E-12 | 1.39E-10 |
| 989 | 3-Hydroxy-1-phenyl-1-eicosanone | 0.1334 | 0.0191 | 2.62E-12 | 2.64E-10 |
| 752 | 13,14-dihydroxy-9-oxo-8,17-dioxatetracyclo[8.7.0.0_,_.0__,__]heptadeca-1(10),2(7),3,5,11(16),12,14-heptaen-5-yl acetate | 0.0692 | 0.0108 | 1.20E-10 | 8.39E-09 |
| 362 | S-(3-oxo-3-carboxy-n-propyl)cysteine | 0.4611 | 0.0732 | 3.00E-10 | 2.01E-08 |
| 72 | Itaconate | 0.1956 | 0.0329 | 2.77E-09 | 1.48E-07 |
| 1512 | PC(18:4(6Z,9Z,12Z,15Z)/P-16:0) | 0.2369 | 0.0415 | 1.16E-08 | 5.67E-07 |
| 332 | Cucurbic acid | 0.0637 | 0.0120 | 1.02E-07 | 3.82E-06 |
| 30 | 2-Thiophenecarboxaldehyde | 0.2368 | 0.0446 | 1.13E-07 | 4.05E-06 |
| 112 | Hydroxyglutarate | 0.0972 | 0.0183 | 1.13E-07 | 4.05E-06 |
| 1088 | 7-a,25-Dihydroxycholesterol | 0.2360 | 0.0468 | 4.50E-07 | 1.31E-05 |
| 66 | Dihydrothymine | 0.1796 | 0.0364 | 7.81E-07 | 2.13E-05 |
| 948 | Erucoylacetone | 0.1589 | 0.0322 | 8.34E-07 | 2.23E-05 |
| 480 | 3,4-Dihydroxyphenylglycol O-sulfate | 0.0398 | 0.0081 | 8.70E-07 | 2.29E-05 |
| 958 | C25:1 | 0.3021 | 0.0619 | 1.04E-06 | 2.70E-05 |
| 691 | Glutathione | 0.1335 | 0.0288 | 3.42E-06 | 7.54E-05 |
| 1074 | MG(0:0/22:0/0:0) | 0.2848 | 0.0618 | 4.09E-06 | 8.76E-05 |
| 191 | (±)-3-Hydroxynonanoic acid | 0.0902 | 0.0197 | 4.85E-06 | 0.0001 |
| 108 | Glutamine | 0.2141 | 0.0482 | 8.96E-06 | 0.0002 |
| 807 | Disaccharide | 0.1057 | 0.0241 | 1.13E-05 | 0.0002 |
| 1591 | Soyasaponin I | 0.0568 | 0.0133 | 1.92E-05 | 0.0004 |
| 1107 | Cysteineglutathione disulfide | 0.1275 | 0.0300 | 2.12E-05 | 0.0004 |
| 211 | 5-Hydroxykynurenamine | 0.0664 | 0.0156 | 2.20E-05 | 0.0004 |
| 272 | Dihydro-5-(2-octenyl)-2(3H)-furanone | 0.0533 | 0.0126 | 2.46E-05 | 0.0004 |
| 1066 | MG(0:0/22:1(13Z)/0:0) | 0.2545 | 0.0610 | 3.06E-05 | 0.0005 |
| 1286 | LysoPE(0:0/20:4(5Z,8Z,11Z,14Z)) | 0.1897 | 0.0464 | 4.35E-05 | 0.0007 |
| 378 | 5-Acetylamino-6-formylamino-3-methyluracil | 0.2180 | 0.0535 | 4.68E-05 | 0.0008 |
| 1443 | TG(10:0/i-12:0/13:0) | 0.2558 | 0.0645 | 7.30E-05 | 0.0011 |
| 578 | 1-(4-Hydroxy-3-methoxyphenyl)-3-decanone | 0.1052 | 0.0266 | 7.77E-05 | 0.0012 |
| 1093 | 5-b-Cholestane-3a ,7a ,12a-triol | 0.2221 | 0.0564 | 8.22E-05 | 0.0012 |
| 983 | NTP | 0.1267 | 0.0322 | 8.32E-05 | 0.0012 |
| 170 | Beta-Carboline | 0.1325 | 0.0345 | 0.0001 | 0.0018 |
| 325 | C13:2 | 0.0548 | 0.0144 | 0.0001 | 0.0020 |
| 1520 | beta-Casomorphin (1-6) | 0.1459 | 0.0384 | 0.0001 | 0.0020 |
| 757 | N-(1-Deoxy-1-fructosyl)phenylalanine | 0.1747 | 0.0462 | 0.0002 | 0.0020 |
| 1297 | ATP | 0.1159 | 0.0307 | 0.0002 | 0.0021 |
| 1018 | 2-Hydroxy-22-methyltetracosanoic acid | 0.2826 | 0.0751 | 0.0002 | 0.0022 |
| 1037 | 7-Ketodeoxycholic acid | 0.1635 | 0.0435 | 0.0002 | 0.0022 |
| 67 | Ethyl tiglate | 0.0323 | 0.0086 | 0.0002 | 0.0022 |
| 1140 | Ximenoylacetone | 0.1194 | 0.0319 | 0.0002 | 0.0023 |
| 904 | C24:1 | 0.2309 | 0.0619 | 0.0002 | 0.0024 |
| 852 | MG(0:0/18:3(6Z,9Z,12Z)/0:0) | 0.0934 | 0.0251 | 0.0002 | 0.0024 |
| 1147 | 5a-Cholestane-3a,7a,12a,25-tetrol | 0.1893 | 0.0511 | 0.0002 | 0.0025 |
| 132 | 2,5-Furandicarboxylic acid | 0.0595 | 0.0161 | 0.0002 | 0.0027 |
| 966 | C25:0 | 0.1558 | 0.0423 | 0.0002 | 0.0027 |
| 171 | 2,6-Dimethoxy-4-methylphenol | 0.0625 | 0.0170 | 0.0002 | 0.0028 |
| 279 | Metharbital | 0.1047 | 0.0285 | 0.0002 | 0.0028 |
| 206 | Propofol | 0.0403 | 0.0110 | 0.0003 | 0.0030 |
| 376 | Bis(2-furanylmethyl) disulfide | 0.1695 | 0.0465 | 0.0003 | 0.0030 |
| 1578 | PS(20:2(11Z,14Z)/24:1(15Z)) | 0.0787 | 0.0216 | 0.0003 | 0.0031 |
| 131 | Histidine | 0.1523 | 0.0420 | 0.0003 | 0.0033 |
| 11 | Succinic aldehyde | 0.0944 | 0.0264 | 0.0004 | 0.0039 |
| 1038 | MG(0:0/22:4(7Z,10Z,13Z,16Z)/0:0) | 0.1798 | 0.0510 | 0.0004 | 0.0044 |
| 1040 | Nervonoylacetone | 0.2073 | 0.0594 | 0.0005 | 0.0051 |
| 1011 | C26:1 | 0.1793 | 0.0514 | 0.0005 | 0.0051 |
| 316 | 4-(1,1,3,3-Tetramethylbutyl)-phenol | 0.0868 | 0.0253 | 0.0006 | 0.0059 |
| 981 | DG(18:0e/2:0/0:0) | 0.1889 | 0.0551 | 0.0006 | 0.0059 |
| 563 | Norophthalmic acid | 0.1700 | 0.0501 | 0.0007 | 0.0065 |
| 1504 | PA(16:0/22:5(4Z,7Z,10Z,13Z,16Z)) | 0.1525 | 0.0453 | 0.0008 | 0.0071 |
| 388 | Pemirolast | 0.0931 | 0.0278 | 0.0008 | 0.0074 |
| 1203 | 13''-Carboxy-alpha-tocopherol | 0.2035 | 0.0608 | 0.0008 | 0.0075 |
| 283 | 3,6-Dihydro-4-(4-methyl-3-pentenyl)-1,2-dithiin | 0.1089 | 0.0327 | 0.0009 | 0.0077 |
| 1032 | MG(0:0/22:5(4Z,7Z,10Z,13Z,16Z)/0:0) | 0.1778 | 0.0536 | 0.0009 | 0.0081 |
| 369 | Rishitin | 0.0442 | 0.0134 | 0.0009 | 0.0084 |
| 1003 | 3b,12a-Dihydroxy-5a-cholanoic acid | 0.1921 | 0.0585 | 0.0010 | 0.0090 |
| 860 | C23:0 | 0.1588 | 0.0486 | 0.0011 | 0.0094 |
| 1568 | PC(o-22:0/22:6(4Z,7Z,10Z,13Z,16Z,19Z)) | 0.0955 | 0.0292 | 0.0011 | 0.0094 |
| 1380 | DTDP-alpha-D-glucose(2-) | 0.1343 | 0.0412 | 0.0011 | 0.0096 |
| 1033 | 3a,7a-Dihydroxy-5b-cholestane | 0.2195 | 0.0676 | 0.0012 | 0.0100 |
| 1080 | Gamma-Tocopherol | 0.1884 | 0.0580 | 0.0012 | 0.0100 |
| 1407 | Bilirubin | 0.2141 | 0.0667 | 0.0013 | 0.0109 |
| 1548 | Goyaglycoside h | 0.0892 | 0.0278 | 0.0013 | 0.0109 |
| 1604 | Galabiosylceramide (d18:1/24:1(15Z)) | 0.0910 | 0.0284 | 0.0013 | 0.0109 |
| 42 | Ketovaline | 0.0424 | 0.0134 | 0.0015 | 0.0122 |
| 354 | Glutamylalanine | 0.1297 | 0.0409 | 0.0015 | 0.0123 |
| 100 | 2,3-Dimethylmaleate | 0.0712 | 0.0225 | 0.0016 | 0.0125 |
| 360 | 1-Propenyl 1-(1-propenylthio)propyl disulfide | 0.1213 | 0.0384 | 0.0016 | 0.0125 |
| 255 | Oxoamide | 0.0948 | 0.0302 | 0.0017 | 0.0133 |
| 809 | Austrobailignan 7 | 0.1895 | 0.0610 | 0.0019 | 0.0148 |
| 659 | All-trans-retinoic acid | 0.0522 | 0.0169 | 0.0020 | 0.0152 |
| 889 | C24:3 | 0.1111 | 0.0361 | 0.0021 | 0.0162 |
| 1211 | 6-Deoxohomodolichosterone | 0.1789 | 0.0585 | 0.0022 | 0.0171 |
| 2 | Acetatic acid | 0.1123 | 0.0370 | 0.0024 | 0.0177 |
| 1086 | 11''-Carboxy-alpha-chromanol | 0.1881 | 0.0619 | 0.0024 | 0.0177 |
| 1210 | Dolichosterone | 0.1881 | 0.0619 | 0.0024 | 0.0177 |
| 988 | Cavipetin C | 0.1505 | 0.0503 | 0.0027 | 0.0198 |
| 844 | Tetrahydrocorticosterone | 0.1638 | 0.0548 | 0.0028 | 0.0201 |
| 1392 | TG(10:0/13:0/8:0) | 0.2410 | 0.0807 | 0.0028 | 0.0201 |
| 1579 | PS(DiMe(11,3)/MonoMe(13,5)) | 0.1276 | 0.0430 | 0.0030 | 0.0213 |
| 1247 | Dolicholide | 0.1603 | 0.0543 | 0.0031 | 0.0218 |
| 1027 | 24-Hydroxycholesterol | 0.1688 | 0.0579 | 0.0035 | 0.0240 |
| 285 | cis-4-Decenedioic acid | 0.0835 | 0.0287 | 0.0036 | 0.0244 |
| 1139 | 3a,7a-Dihydroxycoprostanic acid | 0.1461 | 0.0502 | 0.0036 | 0.0244 |
| 1296 | Theasapogenol A | 0.1816 | 0.0625 | 0.0036 | 0.0244 |
| 64 | 5-Hydroxy-2-furoic acid | 0.0474 | 0.0164 | 0.0038 | 0.0249 |
| 969 | {3-[3-(3,4-dihydroxyphenyl)-3-oxopropyl]-6-hydroxy-2-methoxyphenyl}oxidanesulfonic acid | 0.1059 | 0.0366 | 0.0038 | 0.0249 |
| 1489 | PA(14:1(9Z)/22:2(13Z,16Z)) | 0.1948 | 0.0672 | 0.0038 | 0.0249 |
| 839 | Apigenin 7-sulfate | 0.0650 | 0.0225 | 0.0038 | 0.0249 |
| 1376 | DG(15:0/16:1(9Z)/0:0) | 0.1712 | 0.0594 | 0.0040 | 0.0256 |
| 1014 | C26:0 | 0.1308 | 0.0456 | 0.0042 | 0.0268 |
| 896 | 6-O-Acetylaustroinulin | 0.1623 | 0.0568 | 0.0043 | 0.0275 |
| 838 | 5-Heptadecyl-1,3-benzenediol | 0.1570 | 0.0553 | 0.0045 | 0.0286 |
| 1494 | SM(d18:0/16:1(9Z)) | 0.1403 | 0.0496 | 0.0047 | 0.0293 |
| 210 | Hexose | 0.1878 | 0.0665 | 0.0047 | 0.0298 |
| 1358 | TG(8:0/8:0/a-13:0)[rac] | 0.3046 | 0.1084 | 0.0050 | 0.0310 |
| 1155 | 27-Nor-5b-cholestane-3a,7a,12a,24,25-pentol | 0.2105 | 0.0750 | 0.0050 | 0.0311 |
| 278 | 5-Acetylamino-6-amino-3-methyluracil | 0.4092 | 0.1461 | 0.0051 | 0.0314 |
| 999 | 12-Ketodeoxycholic acid | 0.1511 | 0.0539 | 0.0051 | 0.0314 |
| 374 | 5,8-Tetradecadienoic acid | 0.1736 | 0.0620 | 0.0051 | 0.0317 |
| 1449 | Mactraxanthin | 0.0715 | 0.0256 | 0.0053 | 0.0321 |
| 859 | MG(0:0/18:2(9Z,12Z)/0:0) | 0.1248 | 0.0450 | 0.0055 | 0.0330 |
| 150 | Hydroxyadipate | 0.0397 | 0.0143 | 0.0055 | 0.0331 |
| 1163 | DG(8:0/15:0/0:0) | 0.2122 | 0.0766 | 0.0056 | 0.0332 |
| 756 | Acetaminophen glucuronide | 0.0986 | 0.0356 | 0.0056 | 0.0332 |
| 1514 | PC(15:0/18:3(6Z,9Z,12Z)) | 0.2034 | 0.0734 | 0.0056 | 0.0332 |
| 652 | 3-Oxooctadecanoic acid | 0.1997 | 0.0726 | 0.0060 | 0.0352 |
| 1159 | Momordol | 0.2093 | 0.0762 | 0.0060 | 0.0353 |
| 1420 | Glycerol triundecanoate | 0.3225 | 0.1175 | 0.0060 | 0.0353 |
| 957 | MG(0:0/20:3(11Z,14Z,17Z)/0:0) | 0.1254 | 0.0457 | 0.0061 | 0.0354 |
| 694 | ACRL Toxin II | 0.2538 | 0.0927 | 0.0062 | 0.0357 |
| 1536 | PE(22:4(7Z,10Z,13Z,16Z)/P-18:1(11Z)) | 0.2151 | 0.0786 | 0.0062 | 0.0357 |
| 883 | C24:4 | 0.1007 | 0.0368 | 0.0063 | 0.0360 |
| 703 | Sulfadoxine | 0.0081 | 0.0030 | 0.0063 | 0.0360 |
| 1399 | DG(15:0/18:3(6Z,9Z,12Z)/0:0) | 0.1936 | 0.0711 | 0.0065 | 0.0369 |
| 1191 | 27-Norcholestanehexol | 0.1960 | 0.0721 | 0.0066 | 0.0370 |
| 1195 | DG(8:0/16:0/0:0) | 0.1445 | 0.0531 | 0.0065 | 0.0370 |
| 1263 | Pitheduloside I | 0.1586 | 0.0584 | 0.0066 | 0.0370 |
| 1552 | Glucosylceramide (d18:1/25:0) | 0.0826 | 0.0305 | 0.0067 | 0.0378 |
| 866 | MG(0:0/18:1(11Z)/0:0) | 0.3614 | 0.1335 | 0.0068 | 0.0380 |
| 763 | MG(0:0/16:1(9Z)/0:0) | 0.2218 | 0.0821 | 0.0069 | 0.0382 |
| 429 | 3,4,5-Trimethoxycinnamic acid | 0.0800 | 0.0298 | 0.0073 | 0.0405 |
| 1300 | Fasciculol C | 0.1960 | 0.0733 | 0.0075 | 0.0409 |
| 653 | C19:0 | 0.0668 | 0.0250 | 0.0076 | 0.0411 |
| 1453 | Glucosylceramide (d18:1/12:0) | 0.1507 | 0.0565 | 0.0076 | 0.0411 |
| 80 | Asparagine | 0.1320 | 0.0496 | 0.0079 | 0.0418 |
| 934 | 5-(14-Nonadecenyl)-1,3-benzenediol | 0.1005 | 0.0378 | 0.0078 | 0.0418 |
| 1148 | 14,16-Nonacosanedione | 0.0905 | 0.0341 | 0.0080 | 0.0422 |
| 1160 | MG(0:0/24:1(15Z)/0:0) | 0.1713 | 0.0649 | 0.0082 | 0.0432 |
| 347 | 3-Hydroxydodecanoic acid | 0.0799 | 0.0305 | 0.0087 | 0.0449 |
| 1527 | PC(15:0/20:4(5Z,8Z,11Z,14Z)) | 0.2215 | 0.0847 | 0.0089 | 0.0460 |
| 1133 | 13''-Hydroxy-gamma-tocopherol | 0.1228 | 0.0470 | 0.0090 | 0.0464 |
| 1500 | SM C16:1 | 0.0972 | 0.0373 | 0.0092 | 0.0472 |
| 1390 | (3a,5b,7a)-23-Carboxy-7-hydroxy-24-norcholan-3-yl-b-D-Glucopyranosiduronic acid | 0.1345 | 0.0518 | 0.0095 | 0.0483 |
| 1216 | Trihydroxycoprostanoic acid | 0.1956 | 0.0755 | 0.0096 | 0.0488 |
| 1164 | MG(0:0/24:0/0:0) | 0.2132 | 0.0825 | 0.0098 | 0.0496 |
| 52 | Tetrose | 0.1041 | 0.0403 | 0.0099 | 0.0496 |
| 767 | Furosemide | -0.4076 | 0.0349 | 1.94E-31 | 1.04E-28 |
| 1051 | Heliocide H3 | -0.2405 | 0.0244 | 5.99E-23 | 1.93E-20 |
| 110 | Glutamate | -0.6277 | 0.0701 | 3.55E-19 | 9.50E-17 |
| 1006 | Pteroside Z | -0.2464 | 0.0278 | 7.28E-19 | 1.67E-16 |
| 949 | Sphingosine 1-phosphate | -0.2727 | 0.0323 | 2.87E-17 | 5.77E-15 |
| 626 | 2-Hydroxychlorpropamide | -0.3348 | 0.0417 | 9.82E-16 | 1.75E-13 |
| 1292 | Amprenavir | -0.2825 | 0.0389 | 3.68E-13 | 4.22E-11 |
| 91 | Threonate | -0.4062 | 0.0583 | 3.30E-12 | 3.12E-10 |
| 24 | D-Glyceric acid | -0.4087 | 0.0589 | 3.87E-12 | 3.46E-10 |
| 226 | Phosphoserine | -0.4622 | 0.0689 | 1.98E-11 | 1.68E-09 |
| 1350 | Cyclotricuspidogenin C | -0.2373 | 0.0356 | 2.50E-11 | 2.01E-09 |
| 68 | Cyanuric acid | -0.3935 | 0.0604 | 7.22E-11 | 5.52E-09 |
| 894 | 12-Oxo-20-carboxy-leukotriene B4 | -0.2232 | 0.0344 | 8.61E-11 | 6.29E-09 |
| 668 | 9-(4-Hydroxyphenyl)-2-methoxy-1H-phenalen-1-one | -0.1809 | 0.0290 | 4.21E-10 | 2.70E-08 |
| 184 | Dehydroascorbide(1-) | -0.2660 | 0.0432 | 7.60E-10 | 4.69E-08 |
| 73 | 2-[(Methylthio)methyl]-2-butenal | -0.2485 | 0.0406 | 9.29E-10 | 5.53E-08 |
| 1498 | p-Coumaroyl vitisin A | -0.2545 | 0.0420 | 1.39E-09 | 7.96E-08 |
| 1326 | 2-(2,4-dihydroxy-5-methoxyphenyl)-3-(3,7-dimethylocta-2,6-dien-1-yl)-5,7-dihydroxy-6-(3-methylbut-2-en-1-yl)-3,4-dihydro-2H-1-benzopyran-4-one | -0.1919 | 0.0318 | 1.63E-09 | 9.04E-08 |
| 788 | Tenoxicam | -0.1195 | 0.0204 | 4.93E-09 | 2.55E-07 |
| 1035 | Afzelechin 7-apioside | -0.1515 | 0.0264 | 9.45E-09 | 4.75E-07 |
| 721 | 10-Hydroxy-8-nor-2-fenchanone glucoside | -0.1078 | 0.0189 | 1.21E-08 | 5.73E-07 |
| 1303 | LysoPE(0:0/20:0) | -0.3166 | 0.0560 | 1.56E-08 | 7.05E-07 |
| 1346 | Pyrophaeophorbide a | -0.2113 | 0.0374 | 1.58E-08 | 7.05E-07 |
| 1150 | PE(P-16:0e/0:0) | -0.2480 | 0.0444 | 2.33E-08 | 1.01E-06 |
| 428 | 6-(2-amino-2-carboxyethyl)-4-hydroxybenzothiazole | -0.2995 | 0.0541 | 3.03E-08 | 1.28E-06 |
| 1307 | Deuteroporphyrin IX | -0.2638 | 0.0478 | 3.44E-08 | 1.42E-06 |
| 1049 | 1-Isomangostin | -0.1192 | 0.0217 | 4.21E-08 | 1.69E-06 |
| 451 | N-Undecanoylglycine | -0.1206 | 0.0221 | 4.76E-08 | 1.86E-06 |
| 1253 | LysoPC(15:0) | -0.2807 | 0.0527 | 9.98E-08 | 3.82E-06 |
| 744 | Acetohexamide | -0.2783 | 0.0526 | 1.21E-07 | 4.22E-06 |
| 802 | 15-Deacetylneosolaniol | -0.1698 | 0.0321 | 1.27E-07 | 4.35E-06 |
| 1007 | (R)-2'',4'',7-Trihydroxy-3'',8-diprenylisoflavan | -0.2145 | 0.0408 | 1.43E-07 | 4.79E-06 |
| 1313 | Taurocholic acid | -0.4303 | 0.0832 | 2.34E-07 | 7.67E-06 |
| 1215 | 1-(11Z-eicosenoyl)-glycero-3-phosphate | -0.2488 | 0.0483 | 2.60E-07 | 8.34E-06 |
| 803 | Perindoprilat | -0.3937 | 0.0766 | 2.77E-07 | 8.72E-06 |
| 978 | Mesoridazine | -0.2291 | 0.0451 | 3.74E-07 | 1.15E-05 |
| 351 | 5-Sulfosalicylic acid | -0.0969 | 0.0191 | 4.13E-07 | 1.25E-05 |
| 513 | Ipomeatetrahydrofuran | -0.3419 | 0.0677 | 4.49E-07 | 1.31E-05 |
| 1344 | 2-(6-{2,4-dihydroxy-3-[(1E)-3-methylbut-1-en-1-yl]benzoyl}-5-(2,4-dihydroxyphenyl)-3-(hydroxymethyl)cyclohex-2-en-1-yl)benzene-1,3-diol | -0.2647 | 0.0526 | 4.85E-07 | 1.39E-05 |
| 1217 | Cholylglycine | -0.3365 | 0.0679 | 7.23E-07 | 2.04E-05 |
| 917 | Amylose | -0.2815 | 0.0570 | 7.77E-07 | 2.13E-05 |
| 160 | Methylguanine | -0.2428 | 0.0501 | 1.26E-06 | 3.21E-05 |
| 1276 | LysoPC(16:0) | -0.2245 | 0.0465 | 1.35E-06 | 3.40E-05 |
| 672 | Emedastine | -0.3712 | 0.0771 | 1.48E-06 | 3.67E-05 |
| 437 | Marcanine A | -0.1216 | 0.0253 | 1.59E-06 | 3.87E-05 |
| 1314 | Eriojaposide B | -0.2856 | 0.0598 | 1.81E-06 | 4.35E-05 |
| 534 | Perlolyrine | -0.3329 | 0.0700 | 1.98E-06 | 4.64E-05 |
| 1317 | LysoPC(18:3(6Z,9Z,12Z)) | -0.1888 | 0.0397 | 1.99E-06 | 4.64E-05 |
| 421 | Valerenic acid | -0.1010 | 0.0214 | 2.32E-06 | 5.33E-05 |
| 627 | 3-Hydroxy-9-(4-hydroxyphenyl)-1H,3H-naphtho[1,8-cd]pyran-1-one | -0.2346 | 0.0499 | 2.59E-06 | 5.83E-05 |
| 1383 | PA(8:0/18:0) | -0.2601 | 0.0554 | 2.61E-06 | 5.83E-05 |
| 1515 | PS(15:0/18:3(6Z,9Z,12Z)) | -0.2337 | 0.0506 | 3.91E-06 | 8.49E-05 |
| 1267 | Macrocarpal I | -0.5350 | 0.1168 | 4.61E-06 | 9.75E-05 |
| 69 | Oxoproline | -0.2329 | 0.0510 | 4.91E-06 | 0.0001 |
| 556 | Decarbamoylneosaxitoxin | -0.1373 | 0.0301 | 5.25E-06 | 0.0001 |
| 1050 | Pteroside A | -0.1595 | 0.0351 | 5.63E-06 | 0.0001 |
| 1273 | LysoPC(16:1(9Z)) | -0.2070 | 0.0468 | 9.64E-06 | 0.0002 |
| 377 | 3-Nitrotyrosine | -0.1444 | 0.0329 | 1.17E-05 | 0.0002 |
| 1255 | 11-beta-Hydroxyandrosterone-3-glucuronide | -0.1974 | 0.0451 | 1.21E-05 | 0.0002 |
| 176 | Glyphosate | -0.2172 | 0.0506 | 1.77E-05 | 0.0003 |
| 1405 | Biliverdin | -0.2421 | 0.0570 | 2.19E-05 | 0.0004 |
| 1456 | Chalcomoracin | -0.2010 | 0.0474 | 2.23E-05 | 0.0004 |
| 499 | L-Agaridoxin | -0.1995 | 0.0475 | 2.65E-05 | 0.0005 |
| 1349 | PA(8:0/16:0) | -0.2307 | 0.0560 | 3.76E-05 | 0.0006 |
| 827 | Sorbitan laurate | -0.2536 | 0.0616 | 3.81E-05 | 0.0006 |
| 1063 | Trandolapril-d5 Diketopiperazine | -0.1410 | 0.0343 | 3.88E-05 | 0.0006 |
| 1315 | Probucol | -0.1692 | 0.0421 | 5.83E-05 | 0.0009 |
| 21 | Aminobutanoic acid (ABA) | -0.1225 | 0.0306 | 6.25E-05 | 0.0010 |
| 540 | Neuraminic acid | -0.1094 | 0.0276 | 7.23E-05 | 0.0011 |
| 303 | L-beta-aspartyl-L-alanine | -0.2651 | 0.0670 | 7.52E-05 | 0.0012 |
| 1345 | Austalide D | -0.1717 | 0.0433 | 7.45E-05 | 0.0012 |
| 826 | Cortexolone | -0.1524 | 0.0388 | 8.49E-05 | 0.0013 |
| 533 | N-Acetylcystathionine | -0.1131 | 0.0289 | 9.10E-05 | 0.0013 |
| 943 | Carinol | -0.2053 | 0.0532 | 0.0001 | 0.0016 |
| 81 | D-2-Hydroxyisocaproate | -0.1289 | 0.0335 | 0.0001 | 0.0018 |
| 682 | Arachidonic acid | -0.2403 | 0.0626 | 0.0001 | 0.0018 |
| 731 | Ubiquinone-2 | -0.1438 | 0.0375 | 0.0001 | 0.0018 |
| 828 | Methyl-[10]-shogaol | -0.1980 | 0.0518 | 0.0001 | 0.0018 |
| 1005 | Gibberellin A39 | -0.2140 | 0.0563 | 0.0001 | 0.0020 |
| 1145 | simvastatin hydroxy acid | -0.1261 | 0.0332 | 0.0001 | 0.0020 |
| 862 | Pioglitazone | -0.1779 | 0.0469 | 0.0002 | 0.0020 |
| 942 | Demethylcalabaxanthone | -0.1266 | 0.0337 | 0.0002 | 0.0022 |
| 977 | Edultin | -0.1428 | 0.0384 | 0.0002 | 0.0024 |
| 602 | Avocadyne | -0.2185 | 0.0589 | 0.0002 | 0.0025 |
| 1092 | CPA(18:0/0:0) | -0.1553 | 0.0419 | 0.0002 | 0.0025 |
| 1289 | Crocin 4 | -0.1079 | 0.0294 | 0.0002 | 0.0028 |
| 1100 | Taraxinic acid glucosyl ester | -0.1565 | 0.0430 | 0.0003 | 0.0031 |
| 245 | Bromobenzene-2,3-dihydrodiol | -0.2827 | 0.0783 | 0.0003 | 0.0034 |
| 1196 | 1-(5Z,8Z,11Z,14Z-eicosatetraenoyl)-sn-glycero-3-phosphate | -0.1808 | 0.0502 | 0.0003 | 0.0035 |
| 689 | 5-Androstene-3b,16b,17a-triol | -0.1637 | 0.0462 | 0.0004 | 0.0043 |
| 890 | Isopentenyladenine-9-N-glucoside | -0.1761 | 0.0497 | 0.0004 | 0.0043 |
| 633 | Sulfacytine | -0.1270 | 0.0359 | 0.0004 | 0.0044 |
| 182 | Glycylproline | -0.1613 | 0.0457 | 0.0004 | 0.0044 |
| 671 | 2-Hydroxyestradiol-3-methyl ether | -0.1433 | 0.0411 | 0.0005 | 0.0051 |
| 269 | 1-(Methylthio)propyl propyl disulfide | -0.1413 | 0.0407 | 0.0005 | 0.0053 |
| 1393 | 1-Palmitoylglycerophosphoinositol | -0.1326 | 0.0382 | 0.0005 | 0.0053 |
| 1144 | LPA(0:0/18:1(9Z)) | -0.1717 | 0.0495 | 0.0005 | 0.0053 |
| 307 | Lipoamide | -0.1244 | 0.0359 | 0.0005 | 0.0054 |
| 598 | 2-Phenylethyl beta-D-glucopyranoside | -0.1402 | 0.0407 | 0.0006 | 0.0056 |
| 761 | 7-hydroxygranisetron | -0.1841 | 0.0535 | 0.0006 | 0.0058 |
| 1236 | Austalide B | -0.1048 | 0.0305 | 0.0006 | 0.0058 |
| 1020 | Hemiariensin | -0.1728 | 0.0504 | 0.0006 | 0.0059 |
| 1154 | LPA(0:0/18:0) | -0.2173 | 0.0633 | 0.0006 | 0.0059 |
| 1013 | Isopetasoside | -0.1474 | 0.0431 | 0.0006 | 0.0061 |
| 473 | 5-Hydroxyindoleacetylglycine | -0.1566 | 0.0460 | 0.0007 | 0.0063 |
| 1226 | 20-COOH-leukotriene E4 | -0.1223 | 0.0359 | 0.0007 | 0.0063 |
| 887 | Secoisolariciresinol | -0.1611 | 0.0475 | 0.0007 | 0.0066 |
| 427 | D-Erythro-imidazole-glycerol-phosphate | -0.1635 | 0.0487 | 0.0008 | 0.0073 |
| 575 | Pentoxifylline | -0.1024 | 0.0305 | 0.0008 | 0.0073 |
| 348 | Captopril | -0.1786 | 0.0534 | 0.0008 | 0.0075 |
| 194 | Calystegin B2 | -0.0833 | 0.0249 | 0.0008 | 0.0076 |
| 107 | 2-Methylglutaric acid | -0.0596 | 0.0181 | 0.0010 | 0.0087 |
| 1597 | TG(20:0/22:2(13Z,16Z)/o-18:0) | -0.1583 | 0.0487 | 0.0012 | 0.0100 |
| 651 | Artemether | -0.1649 | 0.0509 | 0.0012 | 0.0100 |
| 576 | Alpha-CEHC | -0.0686 | 0.0212 | 0.0012 | 0.0102 |
| 601 | Retinal | -0.1946 | 0.0603 | 0.0012 | 0.0104 |
| 1218 | Dolichyl b-D-glucosyl phosphate | -0.1318 | 0.0409 | 0.0013 | 0.0106 |
| 661 | Allylestrenol | -0.1505 | 0.0469 | 0.0013 | 0.0109 |
| 477 | C16:4 | -0.1613 | 0.0504 | 0.0014 | 0.0112 |
| 1529 | PS(15:0/20:3(5Z,8Z,11Z)) | -0.1605 | 0.0508 | 0.0016 | 0.0125 |
| 893 | Gibberellin A55 | -0.1506 | 0.0477 | 0.0016 | 0.0125 |
| 830 | 5''-Hydroxypiroxicam | -0.1651 | 0.0524 | 0.0016 | 0.0129 |
| 1531 | Cyanidin 3-(6-caffeoylglucoside) 5-glucoside | -0.1145 | 0.0372 | 0.0021 | 0.0159 |
| 538 | 7-Methoxy-2-methylisoflavone | -0.1168 | 0.0382 | 0.0022 | 0.0171 |
| 1149 | Lagerstroemine | -0.0811 | 0.0266 | 0.0023 | 0.0171 |
| 167 | 2(3H)-Benzothiazolethione | -0.0643 | 0.0212 | 0.0024 | 0.0177 |
| 1042 | 3-O-Methylniveusin A | -0.1820 | 0.0600 | 0.0024 | 0.0177 |
| 1017 | Methyl 3,4-dihydroxy-5-prenylbenzoate 3-glucoside | -0.1664 | 0.0550 | 0.0025 | 0.0182 |
| 1064 | LysoPC(10:0) | -0.1382 | 0.0457 | 0.0025 | 0.0182 |
| 701 | N-Hexadecanoylpyrrolidine | -0.2363 | 0.0785 | 0.0026 | 0.0189 |
| 593 | (-)-Quebrachamine | -0.3319 | 0.1105 | 0.0027 | 0.0192 |
| 674 | Cardanolmonoene | -0.1528 | 0.0511 | 0.0028 | 0.0199 |
| 774 | Glutaminyltryptophan | -0.1120 | 0.0376 | 0.0029 | 0.0206 |
| 1124 | Coumestrin | -0.1088 | 0.0367 | 0.0031 | 0.0215 |
| 1606 | 3-Oxotetradecanoyl-CoA | -0.1194 | 0.0403 | 0.0031 | 0.0215 |
| 1517 | PE-NMe(18:4(6Z,9Z,12Z,15Z)/18:4(6Z,9Z,12Z,15Z)) | -0.1274 | 0.0434 | 0.0033 | 0.0232 |
| 515 | 1,1''-(Tetrahydro-6a-hydroxy-2,3a,5-trimethylfuro[2,3-d]-1,3-dioxole-2,5-diyl)bis-ethanone | -0.0389 | 0.0133 | 0.0034 | 0.0232 |
| 910 | 19-Hydroxy-PGE2 | -0.1214 | 0.0414 | 0.0034 | 0.0232 |
| 1177 | Celastrol | -0.0926 | 0.0316 | 0.0034 | 0.0232 |
| 241 | Kynurenic acid | -0.1291 | 0.0443 | 0.0036 | 0.0241 |
| 854 | 5-[(6-carboxy-3,4,5-trihydroxyoxan-2-yl)oxy]-1H-indole-3-carboxylic acid | -0.1174 | 0.0405 | 0.0038 | 0.0249 |
| 1002 | Calabaxanthone | -0.1391 | 0.0480 | 0.0038 | 0.0249 |
| 1525 | Trabectedin | -0.1205 | 0.0416 | 0.0038 | 0.0249 |
| 1563 | Pectenotoxin 2 | -0.1234 | 0.0428 | 0.0040 | 0.0256 |
| 1222 | Silymonin | -0.0472 | 0.0165 | 0.0044 | 0.0278 |
| 542 | 3-Deoxy-D-glycero-D-galacto-2-nonulosonic acid | -0.1292 | 0.0457 | 0.0046 | 0.0293 |
| 1265 | Edulisin I | -0.1195 | 0.0427 | 0.0052 | 0.0317 |
| 1019 | Niaziminin A | -0.1378 | 0.0494 | 0.0053 | 0.0321 |
| 997 | (2S,4S,6S)-2-[2-(4-Hydroxy-3-meyhoxyphenyl)ethyl]tetrahydro-6-(4,5-dihydroxy-3-methoxyphenyl)-2H-pyran-4-ol | -0.1206 | 0.0433 | 0.0053 | 0.0324 |
| 753 | Humilixanthin | -0.1493 | 0.0537 | 0.0054 | 0.0328 |
| 1290 | (3S,7E,9R)-4,7-Megastigmadiene-3,9-diol 9-[apiosyl-(1->6)-glucoside] | -0.0894 | 0.0322 | 0.0054 | 0.0328 |
| 1052 | LPA(0:0/16:0) | -0.2095 | 0.0764 | 0.0061 | 0.0354 |
| 1001 | SN-38 | -0.1101 | 0.0407 | 0.0068 | 0.0381 |
| 742 | Zeranol | -0.1195 | 0.0444 | 0.0071 | 0.0395 |
| 791 | Omega-Carboxy-trinor-leukotriene B4 | -0.1593 | 0.0596 | 0.0075 | 0.0409 |
| 586 | Feruloylcholine | -0.1533 | 0.0574 | 0.0076 | 0.0411 |
| 816 | 2-(4-Allyl-2-methoxyphenoxy)-1-(4-hydroxy-3-methoxyphenyl)-1-propanol | -0.1746 | 0.0654 | 0.0076 | 0.0411 |
| 1323 | Myricanol 5-glucoside | -0.1110 | 0.0415 | 0.0075 | 0.0411 |
| 1477 | Hv-NCC-1 | -0.1025 | 0.0385 | 0.0078 | 0.0417 |
| 457 | Carbidopa | -0.0527 | 0.0198 | 0.0078 | 0.0417 |
| 518 | 10-Hydroxymyristic acid methyl ester | -0.0837 | 0.0315 | 0.0079 | 0.0420 |
| 754 | (+)-Galeon | -0.1165 | 0.0440 | 0.0081 | 0.0428 |
| 1299 | Acetyl-T2 Toxin | -0.3499 | 0.1324 | 0.0082 | 0.0431 |
| 673 | Eicosapentaenoic acid | -0.2604 | 0.0987 | 0.0083 | 0.0435 |
| 1414 | Agavoside A | -0.0884 | 0.0336 | 0.0085 | 0.0440 |
| 1371 | PA(8:0/17:0) | -0.0904 | 0.0345 | 0.0088 | 0.0455 |
| 1329 | LysoPC(18:0) | -0.1222 | 0.0471 | 0.0095 | 0.0483 |

**B.**

| CFS versus HC | | | | | |
| --- | --- | --- | --- | --- | --- |
| Ion Index | Compound | Coefficient | Std. Error | *P* value | FDR |
| 438 | L-Cystine | 0.3425 | 0.0226 | 6.99E-52 | 1.12E-48 |
| 648 | L-Cysteinylglycine disulfide | 0.3531 | 0.0244 | 1.47E-47 | 1.18E-44 |
| 362 | S-(3-oxo-3-carboxy-n-propyl)cysteine | 1.2638 | 0.1005 | 2.72E-36 | 1.46E-33 |
| 102 | Octanoic acid | 0.4429 | 0.0377 | 7.17E-32 | 2.88E-29 |
| 752 | 13,14-dihydroxy-9-oxo-8,17-dioxatetracyclo[8.7.0.0_,_.0__,__]heptadeca-1(10),2(7),3,5,11(16),12,14-heptaen-5-yl acetate | 0.0907 | 0.0102 | 4.33E-19 | 9.93E-17 |
| 1082 | Shoyuflavone C | 0.0762 | 0.0087 | 2.96E-18 | 4.75E-16 |
| 1 | Acetone | 0.2470 | 0.0342 | 5.13E-13 | 4.58E-11 |
| 99 | 2-oxoglutarate(2-) | 0.0275 | 0.0042 | 7.37E-11 | 5.39E-09 |
| 201 | Cys-Gly | 0.2045 | 0.0314 | 7.08E-11 | 5.39E-09 |
| 332 | Cucurbic acid | 0.0859 | 0.0133 | 9.51E-11 | 6.64E-09 |
| 578 | 1-(4-Hydroxy-3-methoxyphenyl)-3-decanone | 0.1811 | 0.0289 | 3.52E-10 | 2.10E-08 |
| 1512 | PC(18:4(6Z,9Z,12Z,15Z)/P-16:0) | 0.2475 | 0.0396 | 4.04E-10 | 2.32E-08 |
| 480 | 3,4-Dihydroxyphenylglycol O-sulfate | 0.0572 | 0.0093 | 6.66E-10 | 3.57E-08 |
| 989 | 3-Hydroxy-1-phenyl-1-eicosanone | 0.1306 | 0.0214 | 1.02E-09 | 5.28E-08 |
| 30 | 2-Thiophenecarboxaldehyde | 0.2875 | 0.0489 | 4.20E-09 | 1.77E-07 |
| 54 | Cys | 0.3277 | 0.0565 | 6.65E-09 | 2.74E-07 |
| 1552 | Glucosylceramide (d18:1/25:0) | 0.1431 | 0.0257 | 2.60E-08 | 9.50E-07 |
| 325 | C13:2 | 0.0626 | 0.0113 | 2.75E-08 | 9.82E-07 |
| 108 | Glutamine | 0.2583 | 0.0481 | 7.71E-08 | 2.48E-06 |
| 72 | Itaconate | 0.1782 | 0.0333 | 8.43E-08 | 2.66E-06 |
| 1578 | PS(20:2(11Z,14Z)/24:1(15Z)) | 0.1090 | 0.0204 | 9.61E-08 | 2.97E-06 |
| 67 | Ethyl tiglate | 0.0422 | 0.0080 | 1.19E-07 | 3.62E-06 |
| 360 | 1-Propenyl 1-(1-propenylthio)propyl disulfide | 0.1516 | 0.0291 | 1.87E-07 | 5.47E-06 |
| 66 | Dihydrothymine | 0.2006 | 0.0390 | 2.68E-07 | 7.57E-06 |
| 653 | C19:0 | 0.1048 | 0.0204 | 2.84E-07 | 7.73E-06 |
| 659 | All-trans-retinoic acid | 0.0814 | 0.0159 | 2.84E-07 | 7.73E-06 |
| 191 | (±)-3-Hydroxynonanoic acid | 0.0877 | 0.0180 | 1.06E-06 | 2.39E-05 |
| 112 | Hydroxyglutarate | 0.0890 | 0.0183 | 1.12E-06 | 2.50E-05 |
| 1520 | beta-Casomorphin (1-6) | 0.2023 | 0.0424 | 1.79E-06 | 3.78E-05 |
| 1076 | 3,4-dihydroxy-5-{[(2E)-3-[4-hydroxy-3-(sulfooxy)phenyl]prop-2-enoyl]oxy}cyclohex-1-ene-1-carboxylic acid | 0.2098 | 0.0441 | 1.98E-06 | 4.08E-05 |
| 376 | Bis(2-furanylmethyl) disulfide | 0.2255 | 0.0476 | 2.21E-06 | 4.49E-05 |
| 1297 | ATP | 0.1365 | 0.0291 | 2.64E-06 | 5.31E-05 |
| 316 | 4-(1,1,3,3-Tetramethylbutyl)-phenol | 0.1022 | 0.0221 | 3.86E-06 | 7.47E-05 |
| 1607 | CDP-DG(a-13:0/i-22:0) | 0.1148 | 0.0249 | 4.21E-06 | 7.96E-05 |
| 369 | Rishitin | 0.0585 | 0.0127 | 4.38E-06 | 8.18E-05 |
| 206 | Propofol | 0.0462 | 0.0102 | 5.29E-06 | 9.45E-05 |
| 1587 | PS(22:4(7Z,10Z,13Z,16Z)/24:0) | 0.0858 | 0.0192 | 8.15E-06 | 0.0001 |
| 1093 | 5-b-Cholestane-3a ,7a ,12a-triol | 0.2264 | 0.0508 | 8.45E-06 | 0.0001 |
| 904 | C24:1 | 0.2582 | 0.0583 | 9.61E-06 | 0.0002 |
| 1500 | SM C16:1 | 0.1480 | 0.0334 | 9.58E-06 | 0.0002 |
| 1588 | PS(22:1(13Z)/24:1(15Z)) | 0.0960 | 0.0217 | 9.52E-06 | 0.0002 |
| 932 | Calcitroic acid | 0.1220 | 0.0277 | 1.05E-05 | 0.0002 |
| 1443 | TG(10:0/i-12:0/13:0) | 0.2485 | 0.0565 | 1.11E-05 | 0.0002 |
| 1591 | Soyasaponin I | 0.0725 | 0.0167 | 1.51E-05 | 0.0002 |
| 283 | 3,6-Dihydro-4-(4-methyl-3-pentenyl)-1,2-dithiin | 0.1296 | 0.0300 | 1.56E-05 | 0.0002 |
| 794 | Erucic acid | 0.2420 | 0.0564 | 1.80E-05 | 0.0003 |
| 1088 | 7-a,25-Dihydroxycholesterol | 0.2177 | 0.0507 | 1.79E-05 | 0.0003 |
| 36 | 2-Hydroxy-2,4-pentadienoic acid | 0.0758 | 0.0178 | 2.06E-05 | 0.0003 |
| 1079 | 24,25-Dihydroxyvitamin D | 0.1766 | 0.0415 | 2.04E-05 | 0.0003 |
| 1011 | C26:1 | 0.2362 | 0.0559 | 2.37E-05 | 0.0003 |
| 11 | Succinic aldehyde | 0.0895 | 0.0213 | 2.61E-05 | 0.0004 |
| 232 | (E)-2-Tridecene-4,6,8-triyn-1-ol | 0.1306 | 0.0311 | 2.68E-05 | 0.0004 |
| 374 | 5,8-Tetradecadienoic acid | 0.2469 | 0.0598 | 3.66E-05 | 0.0005 |
| 1066 | MG(0:0/22:1(13Z)/0:0) | 0.2327 | 0.0569 | 4.32E-05 | 0.0005 |
| 958 | C25:1 | 0.2757 | 0.0675 | 4.37E-05 | 0.0005 |
| 2 | Acetatic acid | 0.1287 | 0.0318 | 5.22E-05 | 0.0006 |
| 1604 | Galabiosylceramide (d18:1/24:1(15Z)) | 0.1155 | 0.0287 | 5.91E-05 | 0.0007 |
| 100 | 2,3-Dimethylmaleate | 0.0776 | 0.0195 | 7.00E-05 | 0.0008 |
| 757 | N-(1-Deoxy-1-fructosyl)phenylalanine | 0.1858 | 0.0468 | 7.14E-05 | 0.0008 |
| 966 | C25:0 | 0.1611 | 0.0410 | 8.39E-05 | 0.0009 |
| 1334 | LysoPE(0:0/22:6(4Z,7Z,10Z,13Z,16Z,19Z)) | 0.1715 | 0.0436 | 8.36E-05 | 0.0009 |
| 983 | NTP | 0.1178 | 0.0300 | 8.72E-05 | 0.0010 |
| 272 | Dihydro-5-(2-octenyl)-2(3H)-furanone | 0.0551 | 0.0141 | 9.46E-05 | 0.0010 |
| 1147 | 5a-Cholestane-3a,7a,12a,25-tetrol | 0.2485 | 0.0636 | 9.47E-05 | 0.0010 |
| 293 | Sebacic acid | 0.0639 | 0.0164 | 9.70E-05 | 0.0010 |
| 347 | 3-Hydroxydodecanoic acid | 0.0982 | 0.0254 | 0.0001 | 0.0012 |
| 1127 | MG(24:6(6Z,9Z,12Z,15Z,18Z,21Z)/0:0/0:0) | 0.1416 | 0.0372 | 0.0001 | 0.0015 |
| 880 | Dityrosine | 0.1254 | 0.0331 | 0.0002 | 0.0016 |
| 131 | Histidine | 0.1701 | 0.0450 | 0.0002 | 0.0016 |
| 1054 | C27:0 | 0.0952 | 0.0252 | 0.0002 | 0.0016 |
| 297 | Cystathionine ketimine | 0.4058 | 0.1078 | 0.0002 | 0.0017 |
| 1568 | PC(o-22:0/22:6(4Z,7Z,10Z,13Z,16Z,19Z)) | 0.1078 | 0.0288 | 0.0002 | 0.0018 |
| 342 | 3-Oxododecanoic acid | 0.0732 | 0.0197 | 0.0002 | 0.0020 |
| 1140 | Ximenoylacetone | 0.1369 | 0.0369 | 0.0002 | 0.0020 |
| 174 | 10-Undecenal | 0.0648 | 0.0175 | 0.0002 | 0.0020 |
| 716 | Valdecoxib | 0.1932 | 0.0522 | 0.0002 | 0.0020 |
| 1380 | DTDP-alpha-D-glucose(2-) | 0.1848 | 0.0499 | 0.0002 | 0.0020 |
| 64 | 5-Hydroxy-2-furoic acid | 0.0502 | 0.0136 | 0.0002 | 0.0021 |
| 852 | MG(0:0/18:3(6Z,9Z,12Z)/0:0) | 0.1020 | 0.0278 | 0.0002 | 0.0022 |
| 282 | Potassium 2-(1''-ethoxy) ethoxypropanoate | 0.1351 | 0.0371 | 0.0003 | 0.0024 |
| 1354 | 6-{[3,7-dihydroxy-2-(1-hydroxy-3-methoxy-4-oxocyclohexa-2,5-dien-1-yl)-6-methoxy-4-oxo-4H-chromen-5-yl]oxy}-3,4,5-trihydroxyoxane-2-carboxylic acid | 0.1430 | 0.0398 | 0.0003 | 0.0030 |
| 748 | C21:1 | 0.1326 | 0.0370 | 0.0003 | 0.0030 |
| 1504 | PA(16:0/22:5(4Z,7Z,10Z,13Z,16Z)) | 0.1763 | 0.0497 | 0.0004 | 0.0034 |
| 948 | Erucoylacetone | 0.1691 | 0.0481 | 0.0004 | 0.0038 |
| 703 | Sulfadoxine | 0.0100 | 0.0029 | 0.0005 | 0.0040 |
| 158 | Deoxyhexose | 0.1386 | 0.0400 | 0.0005 | 0.0045 |
| 474 | Glutamylthreonine | 0.2018 | 0.0589 | 0.0006 | 0.0051 |
| 763 | MG(0:0/16:1(9Z)/0:0) | 0.1406 | 0.0410 | 0.0006 | 0.0051 |
| 990 | Cyclothiazide | 0.1504 | 0.0439 | 0.0006 | 0.0051 |
| 224 | Citronellyl formate | 0.1880 | 0.0550 | 0.0006 | 0.0052 |
| 251 | 5-Hydroxyindoleacetic acid | 0.1217 | 0.0357 | 0.0006 | 0.0052 |
| 1555 | SM(d19:1/24:1(15Z)) | 0.0851 | 0.0252 | 0.0007 | 0.0059 |
| 18 | Homoserine lactone | 0.1148 | 0.0341 | 0.0008 | 0.0060 |
| 1033 | 3a,7a-Dihydroxy-5b-cholestane | 0.2634 | 0.0783 | 0.0008 | 0.0061 |
| 1548 | Goyaglycoside h | 0.1265 | 0.0378 | 0.0008 | 0.0065 |
| 628 | Edetic Acid | 0.1962 | 0.0587 | 0.0008 | 0.0065 |
| 1074 | MG(0:0/22:0/0:0) | 0.1925 | 0.0576 | 0.0008 | 0.0065 |
| 382 | Methyl dihydrojasmonate | 0.0471 | 0.0141 | 0.0009 | 0.0066 |
| 1489 | PA(14:1(9Z)/22:2(13Z,16Z)) | 0.2130 | 0.0639 | 0.0009 | 0.0066 |
| 168 | Homocysteinesulfinic acid | 0.1027 | 0.0309 | 0.0009 | 0.0066 |
| 785 | Licoagrochalcone B | 0.1398 | 0.0424 | 0.0010 | 0.0073 |
| 5 | Propenoic acid C3:1 | 0.1116 | 0.0340 | 0.0010 | 0.0078 |
| 479 | S-Acetyldihydrolipoamide | 0.0432 | 0.0132 | 0.0011 | 0.0081 |
| 319 | (R)-2-Benzylsuccinate | 0.0526 | 0.0161 | 0.0011 | 0.0082 |
| 896 | 6-O-Acetylaustroinulin | 0.1513 | 0.0464 | 0.0011 | 0.0082 |
| 1579 | PS(DiMe(11,3)/MonoMe(13,5)) | 0.1721 | 0.0528 | 0.0011 | 0.0082 |
| 1300 | Fasciculol C | 0.1946 | 0.0598 | 0.0011 | 0.0082 |
| 988 | Cavipetin C | 0.1314 | 0.0404 | 0.0012 | 0.0083 |
| 171 | 2,6-Dimethoxy-4-methylphenol | 0.0490 | 0.0151 | 0.0012 | 0.0084 |
| 1386 | FAHFA(18:0/9-O-18:0) | 0.2206 | 0.0681 | 0.0012 | 0.0084 |
| 113 | Mevalonate | 0.1095 | 0.0339 | 0.0012 | 0.0086 |
| 39 | Heptanone | 0.1330 | 0.0413 | 0.0013 | 0.0087 |
| 132 | 2,5-Furandicarboxylic acid | 0.0536 | 0.0167 | 0.0013 | 0.0087 |
| 1355 | FAHFA(16:0/9-O-18:0) | 0.2041 | 0.0635 | 0.0013 | 0.0089 |
| 1272 | 10,12-Tritriacontanedione | 0.1196 | 0.0372 | 0.0013 | 0.0089 |
| 25 | 3-Ethylpyridine | 0.0795 | 0.0248 | 0.0013 | 0.0090 |
| 974 | MG(0:0/20:1(11Z)/0:0) | 0.0651 | 0.0203 | 0.0014 | 0.0090 |
| 302 | Daucic acid | 0.1847 | 0.0579 | 0.0014 | 0.0094 |
| 1133 | 13''-Hydroxy-gamma-tocopherol | 0.1871 | 0.0586 | 0.0014 | 0.0094 |
| 1000 | beta-D-3-[5-Deoxy-5-(dimethylarsinyl)ribofuranosyloxy]-2-hydroxy-1-propanesulfonic acid | 0.1995 | 0.0627 | 0.0015 | 0.0097 |
| 969 | {3-[3-(3,4-dihydroxyphenyl)-3-oxopropyl]-6-hydroxy-2-methoxyphenyl}oxidanesulfonic acid | 0.1086 | 0.0345 | 0.0016 | 0.0104 |
| 1308 | Propylene glycol mono- and diesters of fats and fatty acids | 0.1354 | 0.0431 | 0.0017 | 0.0107 |
| 713 | 3D,7D,11D-Phytanic acid | 0.0757 | 0.0241 | 0.0017 | 0.0108 |
| 756 | Acetaminophen glucuronide | 0.1070 | 0.0344 | 0.0019 | 0.0116 |
| 1247 | Dolicholide | 0.1514 | 0.0488 | 0.0019 | 0.0117 |
| 42 | Ketovaline | 0.0356 | 0.0115 | 0.0019 | 0.0118 |
| 136 | Tranexamic Acid | 0.0476 | 0.0153 | 0.0019 | 0.0118 |
| 169 | Urate | 0.1310 | 0.0424 | 0.0020 | 0.0121 |
| 1037 | 7-Ketodeoxycholic acid | 0.1225 | 0.0397 | 0.0020 | 0.0122 |
| 1296 | Theasapogenol A | 0.1612 | 0.0523 | 0.0020 | 0.0122 |
| 652 | 3-Oxooctadecanoic acid | 0.2046 | 0.0664 | 0.0021 | 0.0122 |
| 1335 | Desmosine | 0.0870 | 0.0283 | 0.0021 | 0.0124 |
| 179 | C10:1 | 0.1603 | 0.0522 | 0.0022 | 0.0127 |
| 31 | Furoic acid | 0.0633 | 0.0208 | 0.0023 | 0.0134 |
| 1574 | TG(16:0/18:1(9Z)/20:1(11Z)) | 0.0718 | 0.0235 | 0.0023 | 0.0134 |
| 883 | C24:4 | 0.1232 | 0.0406 | 0.0024 | 0.0140 |
| 3 | Propanol | 0.1304 | 0.0431 | 0.0025 | 0.0142 |
| 486 | C16:3 | 0.1801 | 0.0595 | 0.0025 | 0.0142 |
| 980 | 1-Phenyl-1,3-eicosanedione | 0.0889 | 0.0294 | 0.0025 | 0.0142 |
| 449 | 3-Oxotetradecanoic acid | 0.1209 | 0.0402 | 0.0027 | 0.0151 |
| 1014 | C26:0 | 0.1486 | 0.0495 | 0.0027 | 0.0151 |
| 1211 | 6-Deoxohomodolichosterone | 0.2129 | 0.0711 | 0.0028 | 0.0155 |
| 860 | C23:0 | 0.1489 | 0.0498 | 0.0028 | 0.0156 |
| 1454 | Phosphatidylinositol-3,4,5-trisphosphate | 0.1327 | 0.0445 | 0.0029 | 0.0158 |
| 1032 | MG(0:0/22:5(4Z,7Z,10Z,13Z,16Z)/0:0) | 0.1555 | 0.0523 | 0.0029 | 0.0161 |
| 1203 | 13''-Carboxy-alpha-tocopherol | 0.2049 | 0.0689 | 0.0029 | 0.0161 |
| 663 | Acetylglucosamine sulfate | 0.1528 | 0.0515 | 0.0030 | 0.0164 |
| 866 | MG(0:0/18:1(11Z)/0:0) | 0.1764 | 0.0595 | 0.0030 | 0.0165 |
| 101 | 4-Amino-1-piperidinecarboxylic acid | 0.1632 | 0.0554 | 0.0032 | 0.0173 |
| 805 | Polyoxyethylene (600) monoricinoleate | 0.0518 | 0.0176 | 0.0032 | 0.0173 |
| 441 | L-Menthyl acetoacetate | 0.1265 | 0.0431 | 0.0033 | 0.0176 |
| 783 | Docosatrienoic acid | 0.1007 | 0.0343 | 0.0034 | 0.0179 |
| 563 | Norophthalmic acid | 0.1439 | 0.0492 | 0.0035 | 0.0183 |
| 1210 | Dolichosterone | 0.1528 | 0.0523 | 0.0035 | 0.0183 |
| 1146 | Varanic acid | 0.1405 | 0.0482 | 0.0036 | 0.0187 |
| 17 | Cyclohexanol | 0.0831 | 0.0286 | 0.0036 | 0.0189 |
| 1018 | 2-Hydroxy-22-methyltetracosanoic acid | 0.2331 | 0.0802 | 0.0037 | 0.0191 |
| 38 | Delta-Hexanolactone | 0.0487 | 0.0168 | 0.0038 | 0.0197 |
| 95 | 3,4-Diethylthiophene | 0.0413 | 0.0144 | 0.0040 | 0.0206 |
| 4 | Butynol | 0.0396 | 0.0138 | 0.0042 | 0.0215 |
| 1185 | Cefazolin | 0.1456 | 0.0509 | 0.0042 | 0.0215 |
| 1206 | 6-O-Methylarmillaridin | 0.1549 | 0.0542 | 0.0042 | 0.0215 |
| 211 | 5-Hydroxykynurenamine | 0.0369 | 0.0129 | 0.0044 | 0.0219 |
| 807 | Disaccharide | 0.0595 | 0.0209 | 0.0044 | 0.0221 |
| 1056 | {2,6-dimethoxy-4-[3-oxo-3-(2,4,6-trihydroxyphenyl)prop-1-en-1-yl]phenyl}oxidanesulfonic acid | 0.1766 | 0.0622 | 0.0046 | 0.0226 |
| 889 | C24:3 | 0.0967 | 0.0342 | 0.0047 | 0.0233 |
| 876 | C24:5 | 0.1575 | 0.0559 | 0.0049 | 0.0238 |
| 957 | MG(0:0/20:3(11Z,14Z,17Z)/0:0) | 0.1159 | 0.0412 | 0.0049 | 0.0238 |
| 483 | Isopropyl citrate | 0.0650 | 0.0232 | 0.0051 | 0.0246 |
| 386 | 5-Ethynyl-5''-(1-propynyl)-2,2''-bithiophene | 0.1659 | 0.0594 | 0.0052 | 0.0250 |
| 96 | 4-Oxocyclohexanecarboxylate | 0.0376 | 0.0135 | 0.0053 | 0.0256 |
| 312 | Eugenitol | 0.0987 | 0.0355 | 0.0054 | 0.0258 |
| 1160 | MG(0:0/24:1(15Z)/0:0) | 0.1956 | 0.0703 | 0.0054 | 0.0258 |
| 33 | (Z)-4-Heptenal | 0.0368 | 0.0133 | 0.0055 | 0.0259 |
| 527 | 2-Methoxyacetaminophen sulfate | 0.1006 | 0.0363 | 0.0056 | 0.0263 |
| 126 | (-)-trans-Carveol | 0.1253 | 0.0453 | 0.0057 | 0.0265 |
| 1038 | MG(0:0/22:4(7Z,10Z,13Z,16Z)/0:0) | 0.1550 | 0.0560 | 0.0057 | 0.0265 |
| 1046 | 10,12-Heptacosanedione | 0.1485 | 0.0537 | 0.0057 | 0.0265 |
| 1494 | SM(d18:0/16:1(9Z)) | 0.1448 | 0.0524 | 0.0057 | 0.0267 |
| 1316 | 22-Acetylpriverogenin B | 0.1703 | 0.0617 | 0.0058 | 0.0269 |
| 476 | Gamma-CEHC | 0.0505 | 0.0184 | 0.0059 | 0.0274 |
| 1536 | PE(22:4(7Z,10Z,13Z,16Z)/P-18:1(11Z)) | 0.2169 | 0.0788 | 0.0059 | 0.0274 |
| 1352 | FAHFA(16:1(9Z)/9-O-18:0) | 0.1540 | 0.0565 | 0.0064 | 0.0292 |
| 1475 | C26 Cer | 0.0881 | 0.0323 | 0.0064 | 0.0292 |
| 1139 | 3a,7a-Dihydroxycoprostanic acid | 0.1664 | 0.0611 | 0.0065 | 0.0294 |
| 1252 | Brassinolide | 0.1803 | 0.0664 | 0.0066 | 0.0299 |
| 218 | 5-Nitrosalicylate | 0.0346 | 0.0128 | 0.0068 | 0.0304 |
| 1535 | TG(16:0/14:0/16:1(9Z)) | 0.1037 | 0.0384 | 0.0069 | 0.0308 |
| 1399 | DG(15:0/18:3(6Z,9Z,12Z)/0:0) | 0.2436 | 0.0902 | 0.0069 | 0.0309 |
| 404 | Paracetamol sulfate | 0.2710 | 0.1008 | 0.0072 | 0.0318 |
| 1040 | Nervonoylacetone | 0.1968 | 0.0734 | 0.0073 | 0.0323 |
| 52 | Tetrose | 0.1099 | 0.0410 | 0.0074 | 0.0324 |
| 853 | C23:1 | 0.1046 | 0.0392 | 0.0077 | 0.0337 |
| 1132 | 24-Hydroxycalcitriol | 0.1738 | 0.0652 | 0.0077 | 0.0337 |
| 29 | Histamine | 0.1358 | 0.0511 | 0.0079 | 0.0342 |
| 572 | 2-hydroxy-3-[4-hydroxy-3-(sulfooxy)phenyl]propanoic acid | 0.1128 | 0.0425 | 0.0079 | 0.0342 |
| 1080 | Gamma-Tocopherol | 0.1640 | 0.0619 | 0.0081 | 0.0343 |
| 839 | Apigenin 7-sulfate | 0.0602 | 0.0228 | 0.0082 | 0.0348 |
| 1259 | Bassic acid | 0.1176 | 0.0446 | 0.0084 | 0.0355 |
| 714 | (S)-5''-Deoxy-5''-(methylsulfinyl)adenosine | 0.1006 | 0.0382 | 0.0085 | 0.0357 |
| 875 | 13,14-Dihydro PGF-1a | 0.0961 | 0.0366 | 0.0086 | 0.0360 |
| 495 | 13-Heptadecyn-1-ol | 0.1225 | 0.0466 | 0.0086 | 0.0360 |
| 19 | Oxobutanoic acid | 0.0387 | 0.0147 | 0.0087 | 0.0362 |
| 353 | Triacetin | 0.0735 | 0.0282 | 0.0090 | 0.0374 |
| 1514 | PC(15:0/18:3(6Z,9Z,12Z)) | 0.1665 | 0.0638 | 0.0090 | 0.0374 |
| 604 | Glycineamideribotide | 0.1357 | 0.0520 | 0.0091 | 0.0374 |
| 173 | C10:2 | 0.0622 | 0.0239 | 0.0092 | 0.0377 |
| 743 | {4-[(E)-2-(2,3,5-trihydroxyphenyl)ethenyl]phenyl}oxidanesulfonic acid | 0.0869 | 0.0335 | 0.0095 | 0.0383 |
| 1576 | 3-O-Sulfogalactosylceramide (d18:1/24:0) | 0.0929 | 0.0358 | 0.0095 | 0.0383 |
| 111 | (2R,3R,4R)-2-Amino-4-hydroxy-3-methylpentanoic acid | 0.1169 | 0.0454 | 0.0100 | 0.0398 |
| 459 | Hydroxyprolyl-Isoleucine | 0.0883 | 0.0344 | 0.0104 | 0.0408 |
| 253 | (Iso)Citrate | 0.0686 | 0.0268 | 0.0105 | 0.0409 |
| 660 | MG(0:0/14:1(9Z)/0:0) | 0.0691 | 0.0270 | 0.0105 | 0.0409 |
| 1584 | 3-O-Sulfogalactosylceramide (d18:1/26:1(17Z)) | 0.0734 | 0.0287 | 0.0105 | 0.0409 |
| 1195 | DG(8:0/16:0/0:0) | 0.1582 | 0.0619 | 0.0105 | 0.0410 |
| 387 | 5-phospho-beta-D-ribosylaminium(1-) | 0.1042 | 0.0409 | 0.0108 | 0.0416 |
| 706 | C20:1 | 0.2765 | 0.1084 | 0.0108 | 0.0416 |
| 1453 | Glucosylceramide (d18:1/12:0) | 0.1211 | 0.0475 | 0.0107 | 0.0416 |
| 662 | (R)-3-Hydroxy-Octadecanoic acid | 0.0780 | 0.0307 | 0.0109 | 0.0419 |
| 150 | Hydroxyadipate | 0.0319 | 0.0125 | 0.0111 | 0.0423 |
| 1603 | TG(20:0/20:1(11Z)/20:1(11Z)) | 0.0680 | 0.0269 | 0.0114 | 0.0433 |
| 238 | N-Acetylglutamine | 0.1366 | 0.0541 | 0.0116 | 0.0441 |
| 470 | Threoninyl-Glutamate | 0.0993 | 0.0395 | 0.0119 | 0.0448 |
| 552 | Heptadecanoic acid | 0.0886 | 0.0352 | 0.0119 | 0.0448 |
| 1263 | Pitheduloside I | 0.1298 | 0.0516 | 0.0118 | 0.0448 |
| 1426 | LysoPC(24:1(15Z)) | 0.0962 | 0.0383 | 0.0120 | 0.0449 |
| 462 | 2-Hydroxymyristic acid | 0.0547 | 0.0218 | 0.0122 | 0.0455 |
| 509 | 3-hydroxy-3-(3,4,5-trimethoxyphenyl)propanoic acid | 0.1860 | 0.0742 | 0.0122 | 0.0455 |
| 465 | Marmesin | 0.0975 | 0.0390 | 0.0124 | 0.0461 |
| 62 | 2,3,6-Trihydroxypyridine | 0.0373 | 0.0149 | 0.0125 | 0.0464 |
| 1523 | PC(14:0/20:2(11Z,14Z)) | 0.1745 | 0.0701 | 0.0128 | 0.0473 |
| 787 | Docosadienoate (22:2n6) | 0.0794 | 0.0320 | 0.0130 | 0.0477 |
| 1402 | DG(15:0/18:2(9Z,12Z)/0:0) | 0.2438 | 0.0982 | 0.0130 | 0.0477 |
| 767 | Furosemide | -0.3640 | 0.0334 | 1.17E-27 | 3.76E-25 |
| 1006 | Pteroside Z | -0.2664 | 0.0251 | 2.17E-26 | 5.80E-24 |
| 91 | Threonate | -0.5048 | 0.0572 | 1.04E-18 | 2.10E-16 |
| 110 | Glutamate | -0.5244 | 0.0597 | 1.69E-18 | 3.02E-16 |
| 1051 | Heliocide H3 | -0.2357 | 0.0284 | 1.06E-16 | 1.54E-14 |
| 721 | 10-Hydroxy-8-nor-2-fenchanone glucoside | -0.1159 | 0.0145 | 1.56E-15 | 2.09E-13 |
| 146 | Formyl-Asp | -0.1943 | 0.0248 | 4.39E-15 | 5.42E-13 |
| 1326 | 2-(2,4-dihydroxy-5-methoxyphenyl)-3-(3,7-dimethylocta-2,6-dien-1-yl)-5,7-dihydroxy-6-(3-methylbut-2-en-1-yl)-3,4-dihydro-2H-1-benzopyran-4-one | -0.2626 | 0.0345 | 2.91E-14 | 3.34E-12 |
| 744 | Acetohexamide | -0.2784 | 0.0367 | 3.43E-14 | 3.67E-12 |
| 949 | Sphingosine 1-phosphate | -0.2129 | 0.0287 | 1.11E-13 | 1.11E-11 |
| 685 | 4-Hydroxy-5-(dihydroxyphenyl)-valeric acid-O-sulphate | -0.2665 | 0.0367 | 4.09E-13 | 3.87E-11 |
| 1350 | Cyclotricuspidogenin C | -0.2230 | 0.0316 | 1.71E-12 | 1.44E-10 |
| 978 | Mesoridazine | -0.2373 | 0.0338 | 2.32E-12 | 1.86E-10 |
| 73 | 2-[(Methylthio)methyl]-2-butenal | -0.2253 | 0.0350 | 1.16E-10 | 7.75E-09 |
| 1346 | Pyrophaeophorbide a | -0.1905 | 0.0303 | 3.19E-10 | 2.05E-08 |
| 894 | 12-Oxo-20-carboxy-leukotriene B4 | -0.2583 | 0.0411 | 3.41E-10 | 2.10E-08 |
| 377 | 3-Nitrotyrosine | -0.2088 | 0.0338 | 6.60E-10 | 3.57E-08 |
| 1292 | Amprenavir | -0.2479 | 0.0407 | 1.14E-09 | 5.75E-08 |
| 307 | Lipoamide | -0.1510 | 0.0251 | 1.84E-09 | 8.94E-08 |
| 160 | Methylguanine | -0.2253 | 0.0380 | 2.93E-09 | 1.38E-07 |
| 176 | Glyphosate | -0.2316 | 0.0391 | 3.03E-09 | 1.39E-07 |
| 303 | L-beta-aspartyl-L-alanine | -0.4064 | 0.0691 | 4.11E-09 | 1.77E-07 |
| 802 | 15-Deacetylneosolaniol | -0.1767 | 0.0301 | 4.19E-09 | 1.77E-07 |
| 1307 | Deuteroporphyrin IX | -0.2531 | 0.0439 | 7.93E-09 | 3.19E-07 |
| 1498 | p-Coumaroyl vitisin A | -0.2360 | 0.0411 | 9.65E-09 | 3.78E-07 |
| 626 | 2-Hydroxychlorpropamide | -0.2870 | 0.0502 | 1.11E-08 | 4.26E-07 |
| 226 | Phosphoserine | -0.3684 | 0.0649 | 1.40E-08 | 5.24E-07 |
| 672 | Emedastine | -0.3101 | 0.0562 | 3.38E-08 | 1.18E-06 |
| 68 | Cyanuric acid | -0.3056 | 0.0556 | 3.90E-08 | 1.33E-06 |
| 668 | 9-(4-Hydroxyphenyl)-2-methoxy-1H-phenalen-1-one | -0.1679 | 0.0307 | 4.47E-08 | 1.50E-06 |
| 730 | (S,E)-Zearalenone | -0.1564 | 0.0286 | 4.65E-08 | 1.53E-06 |
| 1005 | Gibberellin A39 | -0.2456 | 0.0465 | 1.27E-07 | 3.77E-06 |
| 451 | N-Undecanoylglycine | -0.0963 | 0.0185 | 1.95E-07 | 5.60E-06 |
| 689 | 5-Androstene-3b,16b,17a-triol | -0.1637 | 0.0319 | 2.95E-07 | 7.90E-06 |
| 1001 | SN-38 | -0.1697 | 0.0332 | 3.26E-07 | 8.60E-06 |
| 1150 | PE(P-16:0e/0:0) | -0.2468 | 0.0485 | 3.54E-07 | 9.18E-06 |
| 1050 | Pteroside A | -0.1947 | 0.0384 | 3.97E-07 | 1.01E-05 |
| 1255 | 11-beta-Hydroxyandrosterone-3-glucuronide | -0.2554 | 0.0505 | 4.27E-07 | 1.07E-05 |
| 556 | Decarbamoylneosaxitoxin | -0.1601 | 0.0318 | 4.84E-07 | 1.20E-05 |
| 803 | Perindoprilat | -0.3598 | 0.0719 | 5.71E-07 | 1.39E-05 |
| 887 | Secoisolariciresinol | -0.2144 | 0.0429 | 5.79E-07 | 1.39E-05 |
| 1049 | 1-Isomangostin | -0.1398 | 0.0281 | 6.86E-07 | 1.62E-05 |
| 351 | 5-Sulfosalicylic acid | -0.0912 | 0.0184 | 7.51E-07 | 1.75E-05 |
| 245 | Bromobenzene-2,3-dihydrodiol | -0.2695 | 0.0545 | 7.63E-07 | 1.75E-05 |
| 650 | Toxin T2 tetrol | -0.0397 | 0.0082 | 1.27E-06 | 2.79E-05 |
| 1013 | Isopetasoside | -0.2723 | 0.0563 | 1.33E-06 | 2.84E-05 |
| 1315 | Probucol | -0.1919 | 0.0397 | 1.33E-06 | 2.84E-05 |
| 1345 | Austalide D | -0.1834 | 0.0384 | 1.82E-06 | 3.79E-05 |
| 24 | D-Glyceric acid | -0.2553 | 0.0549 | 3.25E-06 | 6.44E-05 |
| 968 | N-Acetyllactosamine | -0.1562 | 0.0336 | 3.29E-06 | 6.44E-05 |
| 143 | Ala-Ala | -0.1263 | 0.0274 | 4.11E-06 | 7.87E-05 |
| 651 | Artemether | -0.1666 | 0.0364 | 4.64E-06 | 8.49E-05 |
| 1267 | Macrocarpal I | -0.4361 | 0.0952 | 4.65E-06 | 8.49E-05 |
| 827 | Sorbitan laurate | -0.2561 | 0.0561 | 4.96E-06 | 8.96E-05 |
| 902 | Diacetoxyscirpenol | -0.1378 | 0.0305 | 6.19E-06 | 0.0001 |
| 1313 | Taurocholic acid | -0.4111 | 0.0909 | 6.13E-06 | 0.0001 |
| 534 | Perlolyrine | -0.2936 | 0.0653 | 6.86E-06 | 0.0001 |
| 862 | Pioglitazone | -0.1722 | 0.0383 | 6.91E-06 | 0.0001 |
| 1137 | (12S,15S)-15-O-Demethyl-10,29-dideoxy-11,12-dihydro-striatin C | -0.0770 | 0.0173 | 8.44E-06 | 0.0001 |
| 971 | 3,4,5-trihydroxy-6-{[5-(4-methoxyphenyl)-3-oxopentan-2-yl]oxy}oxane-2-carboxylic acid | -0.1814 | 0.0408 | 8.91E-06 | 0.0001 |
| 409 | Nalidixic Acid | -0.1116 | 0.0252 | 9.34E-06 | 0.0002 |
| 1317 | LysoPC(18:3(6Z,9Z,12Z)) | -0.2090 | 0.0476 | 1.12E-05 | 0.0002 |
| 1215 | 1-(11Z-eicosenoyl)-glycero-3-phosphate | -0.2163 | 0.0496 | 1.31E-05 | 0.0002 |
| 421 | Valerenic acid | -0.0973 | 0.0226 | 1.71E-05 | 0.0003 |
| 977 | Edultin | -0.1584 | 0.0369 | 1.80E-05 | 0.0003 |
| 533 | N-Acetylcystathionine | -0.1085 | 0.0255 | 2.10E-05 | 0.0003 |
| 1007 | (R)-2'',4'',7-Trihydroxy-3'',8-diprenylisoflavan | -0.1770 | 0.0418 | 2.28E-05 | 0.0003 |
| 538 | 7-Methoxy-2-methylisoflavone | -0.1651 | 0.0391 | 2.42E-05 | 0.0003 |
| 457 | Carbidopa | -0.0722 | 0.0172 | 2.58E-05 | 0.0003 |
| 1290 | (3S,7E,9R)-4,7-Megastigmadiene-3,9-diol 9-[apiosyl-(1->6)-glucoside] | -0.1247 | 0.0298 | 2.84E-05 | 0.0004 |
| 661 | Allylestrenol | -0.1762 | 0.0423 | 3.10E-05 | 0.0004 |
| 882 | Quassinol | -0.1733 | 0.0417 | 3.31E-05 | 0.0004 |
| 21 | Aminobutanoic acid (ABA) | -0.1068 | 0.0260 | 3.90E-05 | 0.0005 |
| 428 | 6-(2-amino-2-carboxyethyl)-4-hydroxybenzothiazole | -0.2771 | 0.0680 | 4.58E-05 | 0.0006 |
| 184 | Dehydroascorbide(1-) | -0.1807 | 0.0446 | 5.11E-05 | 0.0006 |
| 513 | Ipomeatetrahydrofuran | -0.2890 | 0.0719 | 5.83E-05 | 0.0007 |
| 540 | Neuraminic acid | -0.1275 | 0.0319 | 6.35E-05 | 0.0008 |
| 602 | Avocadyne | -0.1860 | 0.0465 | 6.41E-05 | 0.0008 |
| 731 | Ubiquinone-2 | -0.1491 | 0.0374 | 6.64E-05 | 0.0008 |
| 1289 | Crocin 4 | -0.1148 | 0.0288 | 6.73E-05 | 0.0008 |
| 69 | Oxoproline | -0.1634 | 0.0413 | 7.45E-05 | 0.0009 |
| 1303 | LysoPE(0:0/20:0) | -0.2462 | 0.0625 | 8.10E-05 | 0.0009 |
| 194 | Calystegin B2 | -0.0810 | 0.0206 | 8.69E-05 | 0.0010 |
| 1344 | 2-(6-{2,4-dihydroxy-3-[(1E)-3-methylbut-1-en-1-yl]benzoyl}-5-(2,4-dihydroxyphenyl)-3-(hydroxymethyl)cyclohex-2-en-1-yl)benzene-1,3-diol | -0.2247 | 0.0573 | 8.80E-05 | 0.0010 |
| 835 | Gibberellin A110 | -0.1238 | 0.0319 | 0.0001 | 0.0011 |
| 230 | 1-Naphthaleneacetic acid | -0.0702 | 0.0181 | 0.0001 | 0.0012 |
| 893 | Gibberellin A55 | -0.2100 | 0.0545 | 0.0001 | 0.0012 |
| 1273 | LysoPC(16:1(9Z)) | -0.1911 | 0.0496 | 0.0001 | 0.0012 |
| 753 | Humilixanthin | -0.1592 | 0.0415 | 0.0001 | 0.0013 |
| 1217 | Cholylglycine | -0.3350 | 0.0885 | 0.0002 | 0.0016 |
| 1515 | PS(15:0/18:3(6Z,9Z,12Z)) | -0.2035 | 0.0543 | 0.0002 | 0.0018 |
| 917 | Amylose | -0.2841 | 0.0768 | 0.0002 | 0.0021 |
| 81 | D-2-Hydroxyisocaproate | -0.1174 | 0.0318 | 0.0002 | 0.0021 |
| 575 | Pentoxifylline | -0.1109 | 0.0301 | 0.0002 | 0.0021 |
| 1035 | Afzelechin 7-apioside | -0.1289 | 0.0354 | 0.0003 | 0.0024 |
| 1253 | LysoPC(15:0) | -0.2458 | 0.0677 | 0.0003 | 0.0026 |
| 788 | Tenoxicam | -0.0881 | 0.0244 | 0.0003 | 0.0027 |
| 700 | N-Acetylneuraminic acid | -0.1366 | 0.0380 | 0.0003 | 0.0029 |
| 1177 | Celastrol | -0.1309 | 0.0366 | 0.0003 | 0.0031 |
| 826 | Cortexolone | -0.1230 | 0.0346 | 0.0004 | 0.0033 |
| 998 | 6,7-Dimethoxy-7-epirosmanol | -0.1867 | 0.0530 | 0.0004 | 0.0037 |
| 371 | Dihydrodioscorine | -0.2191 | 0.0623 | 0.0004 | 0.0038 |
| 1276 | LysoPC(16:0) | -0.1673 | 0.0477 | 0.0005 | 0.0039 |
| 555 | (2S,4R)-4-(9H-Pyrido[3,4-b]indol-1-yl)-1,2,4-butanetriol | -0.1037 | 0.0297 | 0.0005 | 0.0040 |
| 427 | D-Erythro-imidazole-glycerol-phosphate | -0.1695 | 0.0493 | 0.0006 | 0.0049 |
| 1383 | PA(8:0/18:0) | -0.1906 | 0.0555 | 0.0006 | 0.0049 |
| 598 | 2-Phenylethyl beta-D-glucopyranoside | -0.1309 | 0.0386 | 0.0007 | 0.0056 |
| 1002 | Calabaxanthone | -0.1421 | 0.0420 | 0.0007 | 0.0057 |
| 916 | 8''-Episesaminone | -0.1118 | 0.0331 | 0.0007 | 0.0059 |
| 516 | gamma-L-Glutamyl-L-pipecolic acid | -0.1323 | 0.0392 | 0.0007 | 0.0059 |
| 181 | p-Toluenesulfonic acid | -0.1560 | 0.0468 | 0.0009 | 0.0066 |
| 943 | Carinol | -0.2646 | 0.0793 | 0.0009 | 0.0066 |
| 898 | Propericiazine | -0.1259 | 0.0378 | 0.0009 | 0.0066 |
| 683 | Threoninyl-Tryptophan | -0.1191 | 0.0363 | 0.0010 | 0.0076 |
| 1144 | LPA(0:0/18:1(9Z)) | -0.1785 | 0.0545 | 0.0011 | 0.0079 |
| 647 | Hydrochlorothiazide | -0.0994 | 0.0306 | 0.0011 | 0.0082 |
| 1237 | Rubraflavone B | -0.0695 | 0.0214 | 0.0011 | 0.0082 |
| 487 | N-Phenylacetylaspartic acid | -0.0839 | 0.0259 | 0.0012 | 0.0084 |
| 1145 | simvastatin hydroxy acid | -0.0978 | 0.0302 | 0.0012 | 0.0084 |
| 1277 | Glaucarubin | -0.1941 | 0.0600 | 0.0012 | 0.0086 |
| 937 | 18-Oxocortisol | -0.1514 | 0.0469 | 0.0012 | 0.0086 |
| 241 | Kynurenic acid | -0.1306 | 0.0405 | 0.0013 | 0.0087 |
| 816 | 2-(4-Allyl-2-methoxyphenoxy)-1-(4-hydroxy-3-methoxyphenyl)-1-propanol | -0.2077 | 0.0646 | 0.0013 | 0.0089 |
| 1154 | LPA(0:0/18:0) | -0.2018 | 0.0629 | 0.0013 | 0.0089 |
| 1113 | ADP | -0.1833 | 0.0577 | 0.0015 | 0.0098 |
| 781 | 3-(2-Methylpropanoyloxy)-8-(2-methylbutanoyloxy)-9,10-epoxy-p-mentha-1,3,5-triene | -0.1792 | 0.0566 | 0.0015 | 0.0100 |
| 1393 | 1-Palmitoylglycerophosphoinositol | -0.1296 | 0.0410 | 0.0016 | 0.0103 |
| 1531 | Cyanidin 3-(6-caffeoylglucoside) 5-glucoside | -0.1171 | 0.0371 | 0.0016 | 0.0103 |
| 754 | (+)-Galeon | -0.1305 | 0.0414 | 0.0016 | 0.0103 |
| 995 | trans-Piceid | -0.0463 | 0.0147 | 0.0016 | 0.0103 |
| 761 | 7-hydroxygranisetron | -0.1681 | 0.0533 | 0.0016 | 0.0103 |
| 1363 | Physagulin F | -0.1024 | 0.0325 | 0.0016 | 0.0103 |
| 1251 | 11-Oxo-androsterone glucuronide | -0.0962 | 0.0306 | 0.0017 | 0.0105 |
| 1456 | Chalcomoracin | -0.1343 | 0.0428 | 0.0017 | 0.0108 |
| 123 | Xanthine | -0.1468 | 0.0470 | 0.0018 | 0.0111 |
| 863 | Xanthoxylol | -0.1112 | 0.0356 | 0.0018 | 0.0111 |
| 936 | Carissanol | -0.1999 | 0.0646 | 0.0020 | 0.0120 |
| 1063 | Trandolapril-d5 Diketopiperazine | -0.0965 | 0.0312 | 0.0020 | 0.0120 |
| 1201 | 2,3-Secoporrigenin | -0.1952 | 0.0633 | 0.0021 | 0.0122 |
| 755 | 2-Dodecylbenzenesulfonic acid | -0.2087 | 0.0682 | 0.0022 | 0.0129 |
| 1314 | Eriojaposide B | -0.1614 | 0.0530 | 0.0023 | 0.0135 |
| 774 | Glutaminyltryptophan | -0.1336 | 0.0441 | 0.0025 | 0.0142 |
| 1236 | Austalide B | -0.1029 | 0.0342 | 0.0026 | 0.0151 |
| 742 | Zeranol | -0.1682 | 0.0560 | 0.0027 | 0.0151 |
| 1226 | 20-COOH-leukotriene E4 | -0.1036 | 0.0346 | 0.0028 | 0.0155 |
| 1042 | 3-O-Methylniveusin A | -0.2100 | 0.0703 | 0.0028 | 0.0156 |
| 601 | Retinal | -0.1548 | 0.0519 | 0.0028 | 0.0157 |
| 1136 | LPA(0:0/18:2(9Z,12Z)) | -0.1865 | 0.0629 | 0.0030 | 0.0165 |
| 477 | C16:4 | -0.1155 | 0.0391 | 0.0031 | 0.0170 |
| 854 | 5-[(6-carboxy-3,4,5-trihydroxyoxan-2-yl)oxy]-1H-indole-3-carboxylic acid | -0.1153 | 0.0390 | 0.0031 | 0.0170 |
| 1106 | 5,14-bis(acetyloxy)-4-hydroxy-9-oxo-8,17-dioxatetracyclo[8.7.0.0_,_.0__,__]heptadeca-1(10),2,4,6,11,13,15-heptaen-13-yl acetate | -0.0930 | 0.0315 | 0.0032 | 0.0172 |
| 542 | 3-Deoxy-D-glycero-D-galacto-2-nonulosonic acid | -0.1089 | 0.0376 | 0.0038 | 0.0196 |
| 910 | 19-Hydroxy-PGE2 | -0.1017 | 0.0351 | 0.0038 | 0.0196 |
| 1153 | 1-Hydroxy-3,6,7-trimethoxy-2,8-diprenylxanthone | -0.1314 | 0.0454 | 0.0038 | 0.0197 |
| 842 | Calonectrin | -0.1316 | 0.0456 | 0.0039 | 0.0201 |
| 1298 | LysoPE(0:0/20:1(11Z)) | -0.1557 | 0.0545 | 0.0043 | 0.0216 |
| 1190 | Ubiquinone-4 | -0.0958 | 0.0336 | 0.0044 | 0.0221 |
| 1295 | Isolimonic acid | -0.1655 | 0.0583 | 0.0046 | 0.0226 |
| 1009 | 7,8-Dehydro-beta-micropteroxanthin | -0.0849 | 0.0298 | 0.0044 | 0.0221 |
| 1019 | Niaziminin A | -0.1301 | 0.0461 | 0.0047 | 0.0233 |
| 1064 | LysoPC(10:0) | -0.1399 | 0.0496 | 0.0048 | 0.0236 |
| 704 | 6-[2-carboxy-2-(hydroxymethyl)-2-methylethoxy]-3,4,5-trihydroxyoxane-2-carboxylic acid | -0.0574 | 0.0205 | 0.0051 | 0.0246 |
| 890 | Isopentenyladenine-9-N-glucoside | -0.1550 | 0.0553 | 0.0051 | 0.0246 |
| 997 | (2S,4S,6S)-2-[2-(4-Hydroxy-3-meyhoxyphenyl)ethyl]tetrahydro-6-(4,5-dihydroxy-3-methoxyphenyl)-2H-pyran-4-ol | -0.1498 | 0.0535 | 0.0051 | 0.0247 |
| 830 | 5''-Hydroxypiroxicam | -0.1683 | 0.0605 | 0.0054 | 0.0258 |
| 1020 | Hemiariensin | -0.1232 | 0.0444 | 0.0055 | 0.0260 |
| 92 | 2-Methylerythritol | -0.1350 | 0.0492 | 0.0060 | 0.0278 |
| 1285 | Isoaustin | -0.1014 | 0.0370 | 0.0061 | 0.0282 |
| 500 | Methyl 3-(2,3-dihydroxy-3-methylbutyl)-4-hydroxybenzoate | -0.1700 | 0.0621 | 0.0062 | 0.0285 |
| 13 | Pyruvate | -0.0885 | 0.0324 | 0.0063 | 0.0289 |
| 352 | Tyrosol 4-sulfate | -0.1140 | 0.0420 | 0.0067 | 0.0300 |
| 912 | Niazicinin A | -0.1395 | 0.0517 | 0.0070 | 0.0310 |
| 820 | Omeprazole | -0.1020 | 0.0379 | 0.0072 | 0.0318 |
| 122 | 1,2-Benzisothiazol-3(2H)-one | -0.0630 | 0.0237 | 0.0077 | 0.0337 |
| 888 | Cortisol | -0.1703 | 0.0639 | 0.0077 | 0.0337 |
| 343 | Glycerylphosphorylethanolamine | -0.1037 | 0.0390 | 0.0078 | 0.0337 |
| 1529 | PS(15:0/20:3(5Z,8Z,11Z)) | -0.1282 | 0.0483 | 0.0079 | 0.0342 |
| 834 | Gibberellin A60 | -0.1711 | 0.0646 | 0.0080 | 0.0343 |
| 1092 | CPA(18:0/0:0) | -0.1037 | 0.0391 | 0.0081 | 0.0343 |
| 1299 | Acetyl-T2 Toxin | -0.3548 | 0.1338 | 0.0080 | 0.0343 |
| 796 | Rutagravine | -0.0985 | 0.0372 | 0.0081 | 0.0345 |
| 1149 | Lagerstroemine | -0.0636 | 0.0241 | 0.0083 | 0.0349 |
| 828 | Methyl-[10]-shogaol | -0.1424 | 0.0539 | 0.0083 | 0.0350 |
| 1224 | 3,17-Androstanediol glucuronide | -0.1062 | 0.0407 | 0.0090 | 0.0374 |
| 536 | 2-(2-Phenylacetoxy)propionylglycine | -0.1639 | 0.0628 | 0.0091 | 0.0374 |
| 193 | Indole-3-acetate | -0.0380 | 0.0146 | 0.0092 | 0.0377 |
| 884 | 5-Hydroxyomeprazole | -0.1061 | 0.0408 | 0.0092 | 0.0377 |
| 1525 | Trabectedin | -0.1189 | 0.0457 | 0.0093 | 0.0377 |
| 1408 | Montelukast | -0.0960 | 0.0369 | 0.0093 | 0.0379 |
| 411 | N2-Succinyl-L-ornithine | -0.0491 | 0.0189 | 0.0095 | 0.0383 |
| 933 | 3b-Hydroxy-5-cholenoic acid | -0.1441 | 0.0557 | 0.0097 | 0.0390 |
| 586 | Feruloylcholine | -0.1621 | 0.0629 | 0.0100 | 0.0397 |
| 671 | 2-Hydroxyestradiol-3-methyl ether | -0.0998 | 0.0387 | 0.0100 | 0.0397 |
| 1052 | LPA(0:0/16:0) | -0.1883 | 0.0730 | 0.0100 | 0.0397 |
| 1329 | LysoPC(18:0) | -0.0960 | 0.0373 | 0.0100 | 0.0398 |
| 1109 | Mangostanol | -0.0774 | 0.0301 | 0.0101 | 0.0400 |
| 208 | Hippurate | -0.3320 | 0.1295 | 0.0103 | 0.0408 |
| 537 | E-10-Hydroxydesmethylnortriptyline | -0.1807 | 0.0706 | 0.0104 | 0.0409 |
| 633 | Sulfacytine | -0.0963 | 0.0378 | 0.0107 | 0.0416 |
| 299 | Alanyl-Asparagine | -0.0475 | 0.0186 | 0.0108 | 0.0416 |
| 1323 | Myricanol 5-glucoside | -0.1217 | 0.0479 | 0.0110 | 0.0420 |
| 627 | 3-Hydroxy-9-(4-hydroxyphenyl)-1H,3H-naphtho[1,8-cd]pyran-1-one | -0.1423 | 0.0566 | 0.0119 | 0.0448 |
| 1371 | PA(8:0/17:0) | -0.0946 | 0.0379 | 0.0125 | 0.0466 |
| 699 | 3-oxobrimonidine | -0.0501 | 0.0201 | 0.0129 | 0.0474 |
| 1017 | Methyl 3,4-dihydroxy-5-prenylbenzoate 3-glucoside | -0.1316 | 0.0529 | 0.0129 | 0.0474 |
| 908 | Curcumin | -0.1410 | 0.0568 | 0.0130 | 0.0477 |
| 298 | Glycyl-Glutamate | -0.0640 | 0.0258 | 0.0131 | 0.0477 |
| 185 | Acetyl-Glu-semialdehyde | -0.0464 | 0.0188 | 0.0135 | 0.0492 |

**C.**

| QFS versus CFS | | | | | |
| --- | --- | --- | --- | --- | --- |
| Ion Index | Compound | Coefficient | Std. Error | *P* value | FDR |
| 146 | Formyl-Asp | 0.2614 | 0.0262 | 1.81E-23 | 2.91E-20 |
| 648 | L-Cysteinylglycine disulfide | 0.1870 | 0.0321 | 5.84E-09 | 4.69E-06 |
| 685 | 4-Hydroxy-5-(dihydroxyphenyl)-valeric acid-O-sulphate | 0.2744 | 0.0540 | 3.77E-07 | 0.0002 |
| 303 | L-beta-aspartyl-L-alanine | 0.1771 | 0.0378 | 2.82E-06 | 0.0009 |
| 92 | 2-Methylerythritol | 0.1219 | 0.0302 | 5.45E-05 | 0.0110 |
| 143 | Ala-Ala | 0.0785 | 0.0203 | 0.0001 | 0.0189 |
| 438 | L-Cystine | 0.1350 | 0.0355 | 0.0001 | 0.0225 |
| 362 | S-(3-oxo-3-carboxy-n-propyl)cysteine | -0.7235 | 0.1336 | 6.17E-08 | 3.30E-05 |
| 182 | Glycylproline | -0.1491 | 0.0344 | 1.50E-05 | 0.0040 |
| 110 | Glutamate | -0.1426 | 0.0338 | 2.45E-05 | 0.0056 |
| 437 | Marcanine A | -0.0773 | 0.0208 | 0.0002 | 0.0300 |
| 251 | 5-Hydroxyindoleacetic acid | -0.1060 | 0.0296 | 0.0003 | 0.0460 |
