# Supplementary material for: Multi-omics examination of Q fever fatigue syndrome identifies similarities with chronic fatigue syndrome
Source: J Transl Med. 2020 Nov 26;18:448. doi: 10.1186/s12967-020-02585-5 (PMC7690002; doi:10.1186/s12967-020-02585-5)
Supplement: Supplementary file 5 — Additional file 5: Figure S4. Taxonomic differences in gut microbiome composition when comparing QFS to HC, CFS to HC, and QFS to CFS. Volcanoplots showing differences in gut microbiome taxonomy when comparing (A) QFS (n = 31) to HC (n = 50), (B) CFS (n = 50) to HC (n = 50) and (C) QFS (n = 31) tot CFS (n = 50). The gut microbiome composition was assessed by Metagenomic sequencing using the Illumina HiSeq platform Significantly in- and decreased microbes are shown in red and statistical significance was attained if FDR adjusted P ≤ 0.05.Log2FoldChange of Significantly in- and decreased microbes are shown in Additional file 6: Table S2. QFS Q fever fatigue syndrome, HC healthy controls, CFS chronic fatigue syndrome. [file 12967_2020_2585_MOESM5_ESM.pdf]

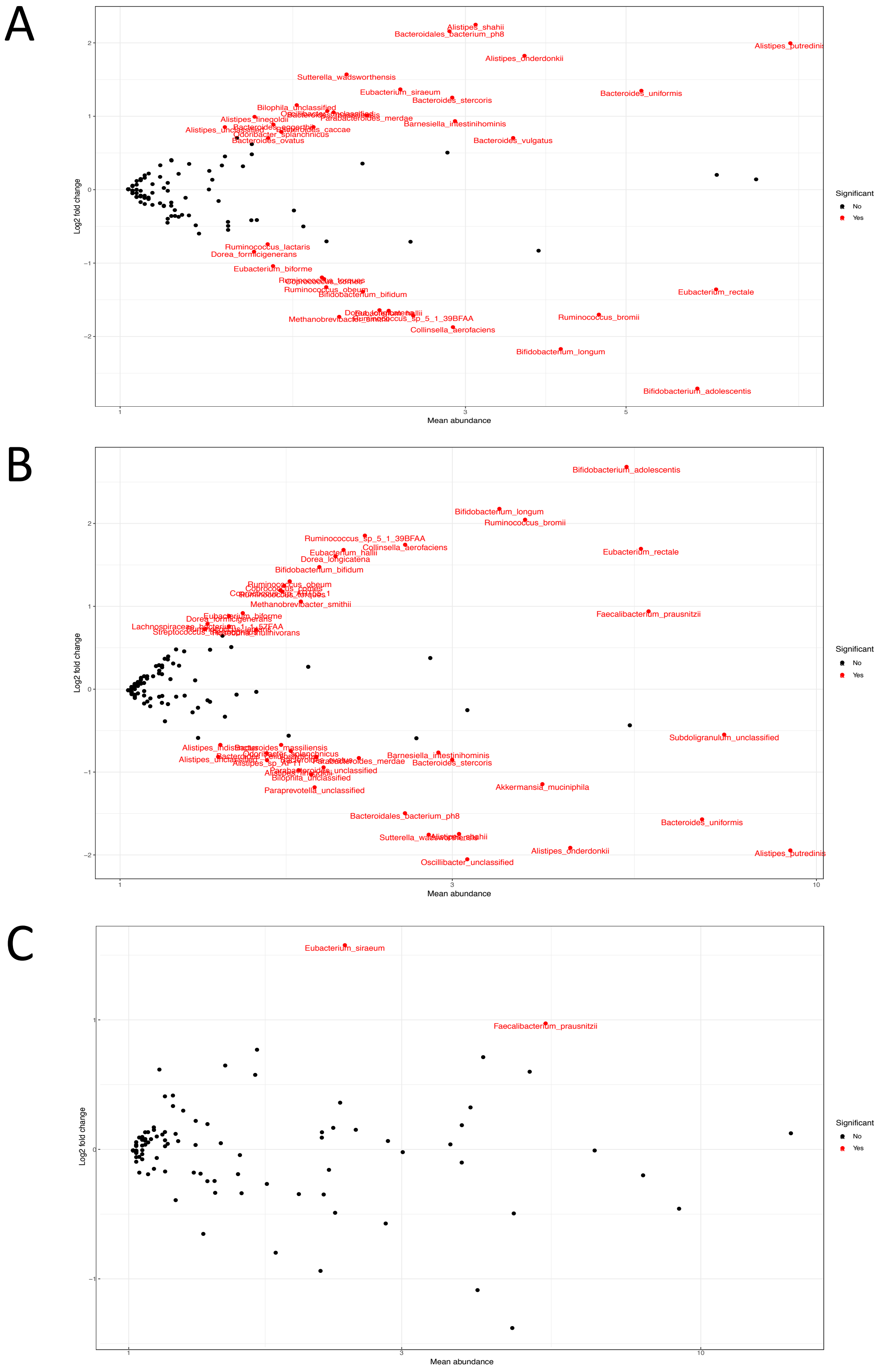
A
Alistipes_shahii
Bacteroidales_bacterium_ph8
Alistipes_putredinis
Alistipes_onderdonkii
Sutterella_wadsworthensis
Eubacterium_siraeum
Bacteroides_stercoris
Bacteroides_uniformis
Bilophila_unclassified
Parabacteroides_merdae
Barnesiella_intestinihominis
Alistipes_finegoldii
Bacteroides_caccae
Odoribacter_splanchnicus
Bacteroides_ovatus
Bacteroides_vulgatus
Ruminococcus_lactaris
Dorea_formicigenerans
Eubacterium_biforme
Ruminococcus_obeum
Bifidobacterium_bifidum
Eubacterium_rectale
Methanobrevibacter_smithii
Ruminococcus_sp_5_1_39BFAA
Ruminococcus_bromii
Collinsella_aerofaciens
Bifidobacterium_longum
Bifidobacterium_adolescentis
Log2 fold change
Mean abundance
Significant
No
Yes
B
Bifidobacterium_adolescentis
Bifidobacterium_longum
Ruminococcus_bromii
Ruminococcus_sp_5_1_39BFAA
Collinsella_aerofaciens
Eubacterium_hallii
Eubacterium_rectale
Dorea_longicatena
Bifidobacterium_bifidum
Ruminococcus_obeum
Methanobrevibacter_smithii
Faecalibacterium_prausnitzii
Eubacterium_biforme
Dorea_formicigenerans
Subdoligranulum_unclassified
Alistipes_indistinctus
Bacteroides_massiliensis
Barnesiella_intestinihominis
Parabacteroides_merdae
Bacteroides_stercoris
Parabacteroides_unclassified
Bilophila_unclassified
Paraprevotella_unclassified
Akkermansia_muciniphila
Bacteroidales_bacterium_ph8
Bacteroides_uniformis
Alistipes_shahii
Alistipes_onderdonkii
Alistipes_putredinis
Oscillibacter_unclassified
Log2 fold change
Mean abundance
Significant
No
Yes
C
Eubacterium_siraeum
Faecalibacterium_prausnitzii
Log2 fold change
Mean abundance
Significant
No
Yes
