# Supplementary material for: Multi-omics examination of Q fever fatigue syndrome identifies similarities with chronic fatigue syndrome
Source: J Transl Med. 2020 Nov 26;18:448. doi: 10.1186/s12967-020-02585-5 (PMC7690002; doi:10.1186/s12967-020-02585-5)
Supplement: Supplementary file 6 — Additional file 6: Table S2. Gut microbiome taxonomic differences when comparing QFS to HC, CFS to HC, and QFS to CFS. Gut microbiome taxonomic differences when comparing (A) QFS (n = 31) to HC (n = 50), (B) CFS (n = 50) to HC (n = 50), and (C) QFS (n = 31) to CFS (n = 50). Results are depicted as Log2FoldChange and significance was attained if adjusted P ≤ 0.05. QFS Q fever fatigue syndrome, HC healthy controls, CFS chronic fatigue syndrome. Statistical significance was attained if adjusted P ≤ 0.05. [file 12967_2020_2585_MOESM6_ESM.docx]

**Table S2. Gut microbiome taxonomic differences when comparing QFS to HC, CFS to HC, and QFS to CFS.**

**A.**

| QFS versus HC | | | |
| --- | --- | --- | --- |
| Taxonomy | Log2FoldChange | Adjusted P value | |
| k__Bacteria\|p__Bacteroidetes\|c__Bacteroidia\|o__Bacteroidales\|f__Rikenellaceae\|g__Alistipes\|s__Alistipes_shahii | 2.2481 | | 8.96E-13 |
| k__Bacteria\|p__Bacteroidetes\|c__Bacteroidia\|o__Bacteroidales\|f__Bacteroidales_noname\|g__Bacteroidales_noname\|s__Bacteroidales_bacterium_ph8 | 2.1585 | | 1.37E-12 |
| k__Bacteria\|p__Actinobacteria\|c__Actinobacteria\|o__Bifidobacteriales\|f__Bifidobacteriaceae\|g__Bifidobacterium\|s__Bifidobacterium_adolescentis | -2.7077 | | 2.21E-12 |
| k__Bacteria\|p__Bacteroidetes\|c__Bacteroidia\|o__Bacteroidales\|f__Rikenellaceae\|g__Alistipes\|s__Alistipes_putredinis | 1.9955 | | 4.11E-12 |
| k__Bacteria\|p__Actinobacteria\|c__Actinobacteria\|o__Bifidobacteriales\|f__Bifidobacteriaceae\|g__Bifidobacterium\|s__Bifidobacterium_longum | -2.1700 | | 2.93E-10 |
| k__Bacteria\|p__Actinobacteria\|c__Actinobacteria\|o__Coriobacteriales\|f__Coriobacteriaceae\|g__Collinsella\|s__Collinsella_aerofaciens | -1.8721 | | 1.06E-08 |
| k__Bacteria\|p__Bacteroidetes\|c__Bacteroidia\|o__Bacteroidales\|f__Rikenellaceae\|g__Alistipes\|s__Alistipes_onderdonkii | 1.8246 | | 8.45E-08 |
| k__Bacteria\|p__Firmicutes\|c__Clostridia\|o__Clostridiales\|f__Ruminococcaceae\|g__Ruminococcus\|s__Ruminococcus_sp_5_1_39BFAA | -1.7157 | | 7.56E-07 |
| k__Bacteria\|p__Firmicutes\|c__Clostridia\|o__Clostridiales\|f__Eubacteriaceae\|g__Eubacterium\|s__Eubacterium_hallii | -1.6483 | | 2.59E-06 |
| k__Bacteria\|p__Firmicutes\|c__Clostridia\|o__Clostridiales\|f__Lachnospiraceae\|g__Dorea\|s__Dorea_longicatena | -1.6404 | | 3.34E-06 |
| k__Archaea\|p__Euryarchaeota\|c__Methanobacteria\|o__Methanobacteriales\|f__Methanobacteriaceae\|g__Methanobrevibacter\|s__Methanobrevibacter_smithii | -1.7309 | | 6.94E-06 |
| k__Bacteria\|p__Firmicutes\|c__Clostridia\|o__Clostridiales\|f__Ruminococcaceae\|g__Ruminococcus\|s__Ruminococcus_bromii | -1.7011 | | 2.05E-05 |
| k__Bacteria\|p__Firmicutes\|c__Clostridia\|o__Clostridiales\|f__Eubacteriaceae\|g__Eubacterium\|s__Eubacterium_rectale | -1.3578 | | 3.44E-05 |
| k__Bacteria\|p__Bacteroidetes\|c__Bacteroidia\|o__Bacteroidales\|f__Bacteroidaceae\|g__Bacteroides\|s__Bacteroides_uniformis | 1.3484 | | 4.95E-05 |
| k__Bacteria\|p__Proteobacteria\|c__Betaproteobacteria\|o__Burkholderiales\|f__Sutterellaceae\|g__Sutterella\|s__Sutterella_wadsworthensis | 1.5710 | | 5.81E-05 |
| k__Bacteria\|p__Firmicutes\|c__Clostridia\|o__Clostridiales\|f__Lachnospiraceae\|g__Blautia\|s__Ruminococcus_obeum | -1.3269 | | 0.0003 |
| k__Bacteria\|p__Bacteroidetes\|c__Bacteroidia\|o__Bacteroidales\|f__Bacteroidaceae\|g__Bacteroides\|s__Bacteroides_stercoris | 1.2552 | | 0.0004 |
| k__Bacteria\|p__Firmicutes\|c__Clostridia\|o__Clostridiales\|f__Eubacteriaceae\|g__Eubacterium\|s__Eubacterium_siraeum | 1.3675 | | 0.0006 |
| k__Bacteria\|p__Firmicutes\|c__Clostridia\|o__Clostridiales\|f__Lachnospiraceae\|g__Coprococcus\|s__Coprococcus_comes | -1.2165 | | 0.0006 |
| k__Bacteria\|p__Actinobacteria\|c__Actinobacteria\|o__Bifidobacteriales\|f__Bifidobacteriaceae\|g__Bifidobacterium\|s__Bifidobacterium_bifidum | -1.3896 | | 0.0009 |
| k__Bacteria\|p__Proteobacteria\|c__Deltaproteobacteria\|o__Desulfovibrionales\|f__Desulfovibrionaceae\|g__Bilophila\|s__Bilophila_unclassified | 1.1520 | | 0.0009 |
| k__Bacteria\|p__Bacteroidetes\|c__Bacteroidia\|o__Bacteroidales\|f__Porphyromonadaceae\|g__Barnesiella\|s__Barnesiella_intestinihominis | 0.9352 | | 0.0010 |
| k__Bacteria\|p__Firmicutes\|c__Clostridia\|o__Clostridiales\|f__Lachnospiraceae\|g__Blautia\|s__Ruminococcus_torques | -1.1949 | | 0.0010 |
| k__Bacteria\|p__Bacteroidetes\|c__Bacteroidia\|o__Bacteroidales\|f__Porphyromonadaceae\|g__Parabacteroides\|s__Parabacteroides_merdae | 1.0108 | | 0.0016 |
| k__Bacteria\|p__Firmicutes\|c__Clostridia\|o__Clostridiales\|f__Oscillospiraceae\|g__Oscillibacter\|s__Oscillibacter_unclassified | 1.0705 | | 0.0020 |
| k__Bacteria\|p__Bacteroidetes\|c__Bacteroidia\|o__Bacteroidales\|f__Bacteroidaceae\|g__Bacteroides\|s__Bacteroides_massiliensis | 1.0531 | | 0.0025 |
| k__Bacteria\|p__Bacteroidetes\|c__Bacteroidia\|o__Bacteroidales\|f__Rikenellaceae\|g__Alistipes\|s__Alistipes_finegoldii | 0.9930 | | 0.0052 |
| k__Bacteria\|p__Firmicutes\|c__Erysipelotrichia\|o__Erysipelotrichales\|f__Erysipelotrichaceae\|g__Erysipelotrichaceae_noname\|s__Eubacterium_biforme | -1.0399 | | 0.0073 |
| k__Bacteria\|p__Bacteroidetes\|c__Bacteroidia\|o__Bacteroidales\|f__Bacteroidaceae\|g__Bacteroides\|s__Bacteroides_caccae | 0.8540 | | 0.0146 |
| k__Bacteria\|p__Bacteroidetes\|c__Bacteroidia\|o__Bacteroidales\|f__Bacteroidaceae\|g__Bacteroides\|s__Bacteroides_eggerthii | 0.8872 | | 0.0198 |
| k__Bacteria\|p__Bacteroidetes\|c__Bacteroidia\|o__Bacteroidales\|f__Porphyromonadaceae\|g__Odoribacter\|s__Odoribacter_splanchnicus | 0.7877 | | 0.0198 |
| k__Bacteria\|p__Bacteroidetes\|c__Bacteroidia\|o__Bacteroidales\|f__Bacteroidaceae\|g__Bacteroides\|s__Bacteroides_vulgatus | 0.7053 | | 0.0228 |
| k__Bacteria\|p__Firmicutes\|c__Clostridia\|o__Clostridiales\|f__Lachnospiraceae\|g__Dorea\|s__Dorea_formicigenerans | -0.8441 | | 0.0256 |
| k__Bacteria\|p__Bacteroidetes\|c__Bacteroidia\|o__Bacteroidales\|f__Rikenellaceae\|g__Alistipes\|s__Alistipes_unclassified | 0.8527 | | 0.0336 |
| k__Bacteria\|p__Bacteroidetes\|c__Bacteroidia\|o__Bacteroidales\|f__Bacteroidaceae\|g__Bacteroides\|s__Bacteroides_ovatus | 0.7041 | | 0.0442 |
| k__Bacteria\|p__Firmicutes\|c__Clostridia\|o__Clostridiales\|f__Ruminococcaceae\|g__Ruminococcus\|s__Ruminococcus_lactaris | -0.7419 | | 0.0460 |

**B.**

| CFS versus HC | | |
| --- | --- | --- |
| Taxonomy | Log2FoldChange | Adjusted P value |
| k__Bacteria\|p__Actinobacteria\|c__Actinobacteria\|o__Bifidobacteriales\|f__Bifidobacteriaceae\|g__Bifidobacterium\|s__Bifidobacterium_adolescentis | 2.6835 | 4.98E-18 |
| k__Bacteria\|p__Actinobacteria\|c__Actinobacteria\|o__Bifidobacteriales\|f__Bifidobacteriaceae\|g__Bifidobacterium\|s__Bifidobacterium_longum | 2.1766 | 1.64E-14 |
| k__Bacteria\|p__Bacteroidetes\|c__Bacteroidia\|o__Bacteroidales\|f__Rikenellaceae\|g__Alistipes\|s__Alistipes_putredinis | -1.9454 | 2.05E-12 |
| k__Bacteria\|p__Actinobacteria\|c__Actinobacteria\|o__Coriobacteriales\|f__Coriobacteriaceae\|g__Collinsella\|s__Collinsella_aerofaciens | 1.7438 | 2.96E-11 |
| k__Bacteria\|p__Firmicutes\|c__Clostridia\|o__Clostridiales\|f__Oscillospiraceae\|g__Oscillibacter\|s__Oscillibacter_unclassified | -2.0520 | 2.96E-11 |
| k__Bacteria\|p__Firmicutes\|c__Clostridia\|o__Clostridiales\|f__Ruminococcaceae\|g__Ruminococcus\|s__Ruminococcus_sp_5_1_39BFAA | 1.8538 | 2.96E-11 |
| k__Bacteria\|p__Firmicutes\|c__Clostridia\|o__Clostridiales\|f__Eubacteriaceae\|g__Eubacterium\|s__Eubacterium_rectale | 1.6942 | 1.30E-10 |
| k__Bacteria\|p__Firmicutes\|c__Clostridia\|o__Clostridiales\|f__Ruminococcaceae\|g__Ruminococcus\|s__Ruminococcus_bromii | 2.0458 | 1.61E-10 |
| k__Bacteria\|p__Firmicutes\|c__Clostridia\|o__Clostridiales\|f__Eubacteriaceae\|g__Eubacterium\|s__Eubacterium_hallii | 1.6809 | 1.68E-09 |
| k__Bacteria\|p__Bacteroidetes\|c__Bacteroidia\|o__Bacteroidales\|f__Rikenellaceae\|g__Alistipes\|s__Alistipes_onderdonkii | -1.9140 | 3.12E-09 |
| k__Bacteria\|p__Bacteroidetes\|c__Bacteroidia\|o__Bacteroidales\|f__Rikenellaceae\|g__Alistipes\|s__Alistipes_shahii | -1.7465 | 7.65E-09 |
| k__Bacteria\|p__Firmicutes\|c__Clostridia\|o__Clostridiales\|f__Lachnospiraceae\|g__Dorea\|s__Dorea_longicatena | 1.6039 | 1.04E-08 |
| k__Bacteria\|p__Bacteroidetes\|c__Bacteroidia\|o__Bacteroidales\|f__Bacteroidales_noname\|g__Bacteroidales_noname\|s__Bacteroidales_bacterium_ph8 | -1.4966 | 5.55E-08 |
| k__Bacteria\|p__Proteobacteria\|c__Betaproteobacteria\|o__Burkholderiales\|f__Sutterellaceae\|g__Sutterella\|s__Sutterella_wadsworthensis | -1.7556 | 3.33E-07 |
| k__Bacteria\|p__Bacteroidetes\|c__Bacteroidia\|o__Bacteroidales\|f__Bacteroidaceae\|g__Bacteroides\|s__Bacteroides_uniformis | -1.5707 | 5.57E-07 |
| k__Bacteria\|p__Firmicutes\|c__Clostridia\|o__Clostridiales\|f__Ruminococcaceae\|g__Faecalibacterium\|s__Faecalibacterium_prausnitzii | 0.9398 | 2.28E-06 |
| k__Bacteria\|p__Firmicutes\|c__Clostridia\|o__Clostridiales\|f__Lachnospiraceae\|g__Blautia\|s__Ruminococcus_obeum | 1.3022 | 7.93E-06 |
| k__Bacteria\|p__Actinobacteria\|c__Actinobacteria\|o__Bifidobacteriales\|f__Bifidobacteriaceae\|g__Bifidobacterium\|s__Bifidobacterium_bifidum | 1.4745 | 8.54E-06 |
| k__Bacteria\|p__Firmicutes\|c__Clostridia\|o__Clostridiales\|f__Lachnospiraceae\|g__Coprococcus\|s__Coprococcus_comes | 1.2489 | 1.85E-05 |
| k__Bacteria\|p__Firmicutes\|c__Clostridia\|o__Clostridiales\|f__Lachnospiraceae\|g__Blautia\|s__Ruminococcus_torques | 1.1788 | 8.60E-05 |
| k__Bacteria\|p__Firmicutes\|c__Clostridia\|o__Clostridiales\|f__Lachnospiraceae\|g__Coprococcus\|s__Coprococcus_sp_ART55_1 | 1.1932 | 0.0003 |
| k__Bacteria\|p__Bacteroidetes\|c__Bacteroidia\|o__Bacteroidales\|f__Prevotellaceae\|g__Paraprevotella\|s__Paraprevotella_unclassified | -1.1840 | 0.0004 |
| k__Bacteria\|p__Proteobacteria\|c__Deltaproteobacteria\|o__Desulfovibrionales\|f__Desulfovibrionaceae\|g__Bilophila\|s__Bilophila_unclassified | -1.0317 | 0.0004 |
| k__Archaea\|p__Euryarchaeota\|c__Methanobacteria\|o__Methanobacteriales\|f__Methanobacteriaceae\|g__Methanobrevibacter\|s__Methanobrevibacter_smithii | 1.0581 | 0.0008 |
| k__Bacteria\|p__Verrucomicrobia\|c__Verrucomicrobiae\|o__Verrucomicrobiales\|f__Verrucomicrobiaceae\|g__Akkermansia\|s__Akkermansia_muciniphila | -1.1454 | 0.0009 |
| k__Bacteria\|p__Bacteroidetes\|c__Bacteroidia\|o__Bacteroidales\|f__Rikenellaceae\|g__Alistipes\|s__Alistipes_finegoldii | -0.9769 | 0.0020 |
| k__Bacteria\|p__Bacteroidetes\|c__Bacteroidia\|o__Bacteroidales\|f__Porphyromonadaceae\|g__Barnesiella\|s__Barnesiella_intestinihominis | -0.7641 | 0.0049 |
| k__Bacteria\|p__Bacteroidetes\|c__Bacteroidia\|o__Bacteroidales\|f__Porphyromonadaceae\|g__Parabacteroides\|s__Parabacteroides_merdae | -0.8306 | 0.0049 |
| k__Bacteria\|p__Firmicutes\|c__Erysipelotrichia\|o__Erysipelotrichales\|f__Erysipelotrichaceae\|g__Erysipelotrichaceae_noname\|s__Eubacterium_biforme | 0.9183 | 0.0050 |
| k__Bacteria\|p__Bacteroidetes\|c__Bacteroidia\|o__Bacteroidales\|f__Porphyromonadaceae\|g__Parabacteroides\|s__Parabacteroides_unclassified | -0.9435 | 0.0051 |
| k__Bacteria\|p__Firmicutes\|c__Clostridia\|o__Clostridiales\|f__Lachnospiraceae\|g__Dorea\|s__Dorea_formicigenerans | 0.8834 | 0.0052 |
| k__Bacteria\|p__Bacteroidetes\|c__Bacteroidia\|o__Bacteroidales\|f__Rikenellaceae\|g__Alistipes\|s__Alistipes_sp_AP11 | -0.8569 | 0.0064 |
| k__Bacteria\|p__Bacteroidetes\|c__Bacteroidia\|o__Bacteroidales\|f__Bacteroidaceae\|g__Bacteroides\|s__Bacteroides_ovatus | -0.8158 | 0.0068 |
| k__Bacteria\|p__Bacteroidetes\|c__Bacteroidia\|o__Bacteroidales\|f__Porphyromonadaceae\|g__Odoribacter\|s__Odoribacter_splanchnicus | -0.7451 | 0.0138 |
| k__Bacteria\|p__Bacteroidetes\|c__Bacteroidia\|o__Bacteroidales\|f__Bacteroidaceae\|g__Bacteroides\|s__Bacteroides_stercoris | -0.8521 | 0.0182 |
| k__Bacteria\|p__Firmicutes\|c__Clostridia\|o__Clostridiales\|f__Ruminococcaceae\|g__Ruminococcus\|s__Ruminococcus_lactaris | 0.7584 | 0.0212 |
| k__Bacteria\|p__Firmicutes\|c__Clostridia\|o__Clostridiales\|f__Lachnospiraceae\|g__Roseburia\|s__Roseburia_inulinivorans | 0.7172 | 0.0218 |
| k__Bacteria\|p__Bacteroidetes\|c__Bacteroidia\|o__Bacteroidales\|f__Bacteroidaceae\|g__Bacteroides\|s__Bacteroides_cellulosilyticus | -0.7716 | 0.0242 |
| k__Bacteria\|p__Bacteroidetes\|c__Bacteroidia\|o__Bacteroidales\|f__Rikenellaceae\|g__Alistipes\|s__Alistipes_unclassified | -0.8152 | 0.0248 |
| k__Bacteria\|p__Firmicutes\|c__Clostridia\|o__Clostridiales\|f__Ruminococcaceae\|g__Subdoligranulum\|s__Subdoligranulum_unclassified | -0.5480 | 0.0259 |
| k__Bacteria\|p__Firmicutes\|c__Clostridia\|o__Clostridiales\|f__Lachnospiraceae\|g__Lachnospiraceae_noname\|s__Lachnospiraceae_bacterium_1_1_57FAA | 0.7887 | 0.0289 |
| k__Bacteria\|p__Firmicutes\|c__Bacilli\|o__Lactobacillales\|f__Streptococcaceae\|g__Streptococcus\|s__Streptococcus_thermophilus | 0.7260 | 0.0336 |
| k__Bacteria\|p__Bacteroidetes\|c__Bacteroidia\|o__Bacteroidales\|f__Bacteroidaceae\|g__Bacteroides\|s__Bacteroides_massiliensis | -0.6720 | 0.0418 |
| k__Bacteria\|p__Bacteroidetes\|c__Bacteroidia\|o__Bacteroidales\|f__Rikenellaceae\|g__Alistipes\|s__Alistipes_indistinctus | -0.6721 | 0.0460 |

**C.**

| QFS versus CFS | | |
| --- | --- | --- |
| Taxonomy | log2FoldChange | Adjusted P value |
| k__Bacteria\|p__Firmicutes\|c__Clostridia\|o__Clostridiales\|f__Eubacteriaceae\|g__Eubacterium\|s__Eubacterium_siraeum | 1.5773 | 0.0078 |
| k__Bacteria\|p__Firmicutes\|c__Clostridia\|o__Clostridiales\|f__Ruminococcaceae\|g__Faecalibacterium\|s__Faecalibacterium_prausnitzii | 0.9727 | 0.0178 |
