# Supplementary material for: Multi-omics examination of Q fever fatigue syndrome identifies similarities with chronic fatigue syndrome
Source: J Transl Med. 2020 Nov 26;18:448. doi: 10.1186/s12967-020-02585-5 (PMC7690002; doi:10.1186/s12967-020-02585-5)
Supplement: Supplementary file 7 — Additional file 7: Table S3. Gut microbiome functional differences when comparing QFS to HC, CFS to HC, and QFS to CFS. Gut microbiome functional differences when comparing (A) QFS (n = 31) to HC (n = 50), (B) CFS (n = 50) to HC (n = 50), and (C) QFS (n = 31) to CFS (n = 50). Results are depicted as Log2FoldChange and significance was attained if adjusted P ≤ 0.05. QFS Q fever fatigue syndrome, HC healthy controls, CFS chronic fatigue syndrome. [file 12967_2020_2585_MOESM7_ESM.docx]

**Table S3. Gut microbiome functional differences when comparing QFS to HC, CFS to HC, and QFS to CFS.**

**A.**

| QFS versus HC | | |
| --- | --- | --- |
| Function | Log2FoldChange | Adjusted P value |
| OANTIGEN-PWY: O-antigen building blocks biosynthesis (E. coli) | -2.1921 | 9.66E-29 |
| PWY-5695: urate biosynthesis/inosine 5'-phosphate degradation | 0.9723 | 9.66E-29 |
| TRPSYN-PWY: L-tryptophan biosynthesis | -1.3677 | 5.55E-26 |
| UDPNAGSYN-PWY: UDP-N-acetyl-D-glucosamine biosynthesis I | -2.7467 | 5.55E-26 |
| PWY-1269: CMP-3-deoxy-D-manno-octulosonate biosynthesis I | 1.9528 | 9.83E-22 |
| PWY-6936: seleno-amino acid biosynthesis | -1.8397 | 1.37E-20 |
| HISTSYN-PWY: L-histidine biosynthesis | -1.0883 | 6.19E-20 |
| PWY-621: sucrose degradation III (sucrose invertase) | -1.2568 | 1.27E-19 |
| PWY-7229: superpathway of adenosine nucleotides de novo biosynthesis I | 0.8173 | 5.95E-18 |
| MET-SAM-PWY: superpathway of S-adenosyl-L-methionine biosynthesis | -1.3423 | 3.20E-17 |
| PYRIDOXSYN-PWY: pyridoxal 5'-phosphate biosynthesis I | 1.9560 | 4.86E-17 |
| PWY0-166: superpathway of pyrimidine deoxyribonucleotides de novo biosynthesis (E. coli) | 0.8682 | 1.29E-16 |
| HOMOSER-METSYN-PWY: L-methionine biosynthesis I | -1.3979 | 2.19E-16 |
| PWY-5104: L-isoleucine biosynthesis IV | -1.8289 | 2.19E-16 |
| PWY-5347: superpathway of L-methionine biosynthesis (transsulfuration) | -1.1118 | 4.45E-16 |
| PWY-7184: pyrimidine deoxyribonucleotides de novo biosynthesis I | 1.0480 | 2.57E-15 |
| PWY-7220: adenosine deoxyribonucleotides de novo biosynthesis II | 1.0618 | 2.97E-15 |
| PWY-7222: guanosine deoxyribonucleotides de novo biosynthesis II | 1.0618 | 2.97E-15 |
| METSYN-PWY: L-homoserine and L-methionine biosynthesis | -1.1497 | 3.03E-15 |
| PWY-6125: superpathway of guanosine nucleotides de novo biosynthesis II | 1.0072 | 3.24E-15 |
| PWY-5188: tetrapyrrole biosynthesis I (from glutamate) | -0.9182 | 3.40E-15 |
| PWY66-409: superpathway of purine nucleotide salvage | -1.7805 | 3.40E-15 |
| PWY-6126: superpathway of adenosine nucleotides de novo biosynthesis II | 0.8791 | 4.36E-15 |
| PWY66-422: D-galactose degradation V (Leloir pathway) | -0.8702 | 8.09E-15 |
| PANTO-PWY: phosphopantothenate biosynthesis I | 0.6186 | 1.51E-14 |
| PWY-6317: galactose degradation I (Leloir pathway) | -0.8311 | 3.52E-14 |
| ARGININE-SYN4-PWY: L-ornithine de novo biosynthesis | 2.1611 | 6.28E-14 |
| PWY-7197: pyrimidine deoxyribonucleotide phosphorylation | 1.0268 | 1.25E-13 |
| PWY0-845: superpathway of pyridoxal 5'-phosphate biosynthesis and salvage | 1.5919 | 1.44E-13 |
| PWY-7228: superpathway of guanosine nucleotides de novo biosynthesis I | 0.9824 | 3.15E-13 |
| PWY0-781: aspartate superpathway | -1.3872 | 6.97E-13 |
| GLYCOGENSYNTH-PWY: glycogen biosynthesis I (from ADP-D-Glucose) | -1.4010 | 8.53E-13 |
| COMPLETE-ARO-PWY: superpathway of aromatic amino acid biosynthesis | -0.3131 | 1.06E-11 |
| P4-PWY: superpathway of L-lysine, L-threonine and L-methionine biosynthesis I | -1.3219 | 1.31E-11 |
| PWY-6703: preQ0 biosynthesis | 0.8287 | 1.97E-11 |
| PWY-7357: thiamin formation from pyrithiamine and oxythiamine (yeast) | -0.6307 | 2.81E-11 |
| ARO-PWY: chorismate biosynthesis I | -0.3317 | 6.70E-11 |
| 1CMET2-PWY: N10-formyl-tetrahydrofolate biosynthesis | 0.7538 | 1.92E-10 |
| PWY-5973: cis-vaccenate biosynthesis | 0.6826 | 2.41E-10 |
| PWY-7663: gondoate biosynthesis (anaerobic) | 0.7585 | 2.75E-10 |
| PWY-7400: L-arginine biosynthesis IV (archaebacteria) | -1.2788 | 2.78E-10 |
| PWY-6163: chorismate biosynthesis from 3-dehydroquinate | -0.3596 | 3.53E-10 |
| PWY-3841: folate transformations II | 0.7483 | 6.78E-10 |
| ARGSYN-PWY: L-arginine biosynthesis I (via L-ornithine) | -1.3146 | 8.80E-10 |
| ARGSYNBSUB-PWY: L-arginine biosynthesis II (acetyl cycle) | -1.5021 | 1.34E-09 |
| PWY-2942: L-lysine biosynthesis III | 0.3975 | 1.76E-09 |
| HISDEG-PWY: L-histidine degradation I | 1.6809 | 2.11E-09 |
| PWY-5100: pyruvate fermentation to acetate and lactate II | -0.7569 | 2.57E-09 |
| GLUTORN-PWY: L-ornithine biosynthesis | -1.5776 | 3.03E-09 |
| PWY4FS-7: phosphatidylglycerol biosynthesis I (plastidic) | 1.4365 | 3.57E-09 |
| PWY4FS-8: phosphatidylglycerol biosynthesis II (non-plastidic) | 1.4367 | 3.57E-09 |
| PWY-7196: superpathway of pyrimidine ribonucleosides salvage | -1.4291 | 8.06E-09 |
| PWY-6608: guanosine nucleotides degradation III | -0.8519 | 2.02E-08 |
| PWY-6385: peptidoglycan biosynthesis III (mycobacteria) | 0.5369 | 2.82E-08 |
| SALVADEHYPOX-PWY: adenosine nucleotides degradation II | -0.9409 | 2.82E-08 |
| PWY-6527: stachyose degradation | -0.6554 | 8.90E-08 |
| PWY-5367: petroselinate biosynthesis | -2.0256 | 2.00E-07 |
| PWY-7208: superpathway of pyrimidine nucleobases salvage | 0.6277 | 2.00E-07 |
| PEPTIDOGLYCANSYN-PWY: peptidoglycan biosynthesis I (meso-diaminopimelate containing) | 0.3290 | 2.07E-07 |
| PWY-5103: L-isoleucine biosynthesis III | -0.5619 | 2.53E-07 |
| PWY-6387: UDP-N-acetylmuramoyl-pentapeptide biosynthesis I (meso-diaminopimelate containing) | 0.3113 | 5.44E-07 |
| PWY-6168: flavin biosynthesis III (fungi) | 0.7348 | 6.20E-07 |
| BRANCHED-CHAIN-AA-SYN-PWY: superpathway of branched amino acid biosynthesis | -0.5026 | 6.99E-07 |
| PWY-6545: pyrimidine deoxyribonucleotides de novo biosynthesis III | 0.5743 | 6.99E-07 |
| PWY-5686: UMP biosynthesis | 0.2381 | 1.01E-06 |
| PWY-6386: UDP-N-acetylmuramoyl-pentapeptide biosynthesis II (lysine-containing) | 0.3062 | 1.19E-06 |
| PHOSLIPSYN-PWY: superpathway of phospholipid biosynthesis I (bacteria) | 1.0517 | 1.72E-06 |
| ANAGLYCOLYSIS-PWY: glycolysis III (from glucose) | 0.3039 | 6.34E-06 |
| DENOVOPURINE2-PWY: superpathway of purine nucleotides de novo biosynthesis II | 0.3387 | 7.08E-06 |
| PWY-5097: L-lysine biosynthesis VI | 0.2797 | 8.82E-06 |
| PENTOSE-P-PWY: pentose phosphate pathway | -0.9221 | 2.64E-05 |
| PWY-5659: GDP-mannose biosynthesis | 0.5413 | 4.02E-05 |
| PWY-6609: adenine and adenosine salvage III | -0.3605 | 5.21E-05 |
| PWY-6147: 6-hydroxymethyl-dihydropterin diphosphate biosynthesis I | -1.0674 | 6.55E-05 |
| ASPASN-PWY: superpathway of L-aspartate and L-asparagine biosynthesis | 0.3594 | 7.61E-05 |
| PWY-5989: stearate biosynthesis II (bacteria and plants) | -0.9545 | 0.0002 |
| PWY-7221: guanosine ribonucleotides de novo biosynthesis | 0.2219 | 0.0002 |
| PWY-5101: L-isoleucine biosynthesis II | 0.7802 | 0.0003 |
| PWY-7282: 4-amino-2-methyl-5-phosphomethylpyrimidine biosynthesis (yeast) | 0.5384 | 0.0004 |
| PWY-6123: inosine-5'-phosphate biosynthesis I | 0.3228 | 0.0005 |
| GALACTUROCAT-PWY: D-galacturonate degradation I | 0.5133 | 0.0005 |
| RIBOSYN2-PWY: flavin biosynthesis I (bacteria and plants) | 0.9195 | 0.0006 |
| PYRIDNUCSYN-PWY: NAD biosynthesis I (from aspartate) | 0.3994 | 0.0006 |
| PWY-6700: queuosine biosynthesis | 0.4008 | 0.0007 |
| THISYNARA-PWY: superpathway of thiamin diphosphate biosynthesis III (eukaryotes) | -0.4299 | 0.0007 |
| HSERMETANA-PWY: L-methionine biosynthesis III | 0.4469 | 0.0011 |
| NONMEVIPP-PWY: methylerythritol phosphate pathway I | 0.2329 | 0.0012 |
| UNINTEGRATED | 0.1705 | 0.0012 |
| PWY-7211: superpathway of pyrimidine deoxyribonucleotides de novo biosynthesis | -0.6039 | 0.0017 |
| PWY-7187: pyrimidine deoxyribonucleotides de novo biosynthesis II | 0.2654 | 0.0021 |
| PWY0-162: superpathway of pyrimidine ribonucleotides de novo biosynthesis | 0.3735 | 0.0021 |
| PRPP-PWY: superpathway of histidine, purine, and pyrimidine biosynthesis | -0.4303 | 0.0022 |
| GALACT-GLUCUROCAT-PWY: superpathway of hexuronide and hexuronate degradation | 0.4192 | 0.0023 |
| PWY-5484: glycolysis II (from fructose 6-phosphate) | 0.6609 | 0.0024 |
| PWY-7560: methylerythritol phosphate pathway II | -1.1308 | 0.0027 |
| TRNA-CHARGING-PWY: tRNA charging | 0.2969 | 0.0033 |
| PWY-6270: isoprene biosynthesis I | -1.1179 | 0.0042 |
| UNMAPPED | 0.2523 | 0.0046 |
| COBALSYN-PWY: adenosylcobalamin salvage from cobinamide I | -0.3605 | 0.0050 |
| COA-PWY-1: coenzyme A biosynthesis II (mammalian) | 0.1814 | 0.0052 |
| GLYCOLYSIS: glycolysis I (from glucose 6-phosphate) | 0.6058 | 0.0059 |
| PWY-724: superpathway of L-lysine, L-threonine and L-methionine biosynthesis II | 0.1425 | 0.0100 |
| PWY-841: superpathway of purine nucleotides de novo biosynthesis I | 0.2791 | 0.0111 |
| PWY-3001: superpathway of L-isoleucine biosynthesis I | -0.1921 | 0.0113 |
| PWY-6121: 5-aminoimidazole ribonucleotide biosynthesis I | 0.1227 | 0.0136 |
| ANAEROFRUCAT-PWY: homolactic fermentation | 0.3955 | 0.0139 |
| COA-PWY: coenzyme A biosynthesis I | -0.2774 | 0.0203 |
| GLCMANNANAUT-PWY: superpathway of N-acetylglucosamine, N-acetylmannosamine and N-acetylneuraminate degradation | -0.2908 | 0.0320 |
| PWY-7199: pyrimidine deoxyribonucleosides salvage | 0.2970 | 0.0392 |
| PWY-7234: inosine-5'-phosphate biosynthesis III | 0.3254 | 0.0473 |

**B.**

| CFS versus HC | | |
| --- | --- | --- |
| Function | Log2FoldChange | Adjusted P value |
| PWY-5695: urate biosynthesis/inosine 5'-phosphate degradation | -1.0605 | 1.31E-54 |
| PWY-621: sucrose degradation III (sucrose invertase) | 2.1613 | 3.76E-52 |
| HISTSYN-PWY: L-histidine biosynthesis | 1.3661 | 3.15E-43 |
| PWY-6317: galactose degradation I (Leloir pathway) | 1.3148 | 5.00E-41 |
| PWY66-422: D-galactose degradation V (Leloir pathway) | 1.3539 | 3.54E-40 |
| PANTO-PWY: phosphopantothenate biosynthesis I | -0.8486 | 5.11E-40 |
| PYRIDOXSYN-PWY: pyridoxal 5'-phosphate biosynthesis I | -2.3482 | 6.27E-37 |
| GLYCOGENSYNTH-PWY: glycogen biosynthesis I (from ADP-D-Glucose) | 1.9833 | 6.97E-35 |
| PWY-1269: CMP-3-deoxy-D-manno-octulosonate biosynthesis I | -2.0405 | 8.61E-33 |
| PWY-6703: preQ0 biosynthesis | -1.1011 | 3.45E-31 |
| PWY-6527: stachyose degradation | 1.1923 | 2.23E-29 |
| PWY-2942: L-lysine biosynthesis III | -0.7050 | 9.12E-29 |
| PWY-7357: thiamin formation from pyrithiamine and oxythiamine (yeast) | 1.0222 | 7.29E-28 |
| PWY0-845: superpathway of pyridoxal 5'-phosphate biosynthesis and salvage | -1.9039 | 1.13E-27 |
| PWY-7196: superpathway of pyrimidine ribonucleosides salvage | 2.4408 | 5.13E-26 |
| PWY-7229: superpathway of adenosine nucleotides de novo biosynthesis I | -0.7906 | 5.13E-26 |
| PWY-5030: L-histidine degradation III | -2.1146 | 1.09E-25 |
| UDPNAGSYN-PWY: UDP-N-acetyl-D-glucosamine biosynthesis I | 2.6758 | 1.57E-25 |
| OANTIGEN-PWY: O-antigen building blocks biosynthesis (E. coli) | 2.2534 | 1.75E-24 |
| 1CMET2-PWY: N10-formyl-tetrahydrofolate biosynthesis | -0.9835 | 2.45E-24 |
| HISDEG-PWY: L-histidine degradation I | -2.0520 | 4.11E-24 |
| ARGININE-SYN4-PWY: L-ornithine de novo biosynthesis | -2.2799 | 1.70E-23 |
| PWY-3841: folate transformations II | -0.9642 | 7.63E-22 |
| PWY-6126: superpathway of adenosine nucleotides de novo biosynthesis II | -0.8091 | 1.87E-21 |
| PWY0-781: aspartate superpathway | 1.5418 | 1.87E-21 |
| PWY-5347: superpathway of L-methionine biosynthesis (transsulfuration) | 1.1894 | 5.84E-21 |
| PWY-7228: superpathway of guanosine nucleotides de novo biosynthesis I | -1.0577 | 5.84E-21 |
| PWY-6125: superpathway of guanosine nucleotides de novo biosynthesis II | -0.9269 | 1.53E-20 |
| PEPTIDOGLYCANSYN-PWY: peptidoglycan biosynthesis I (meso-diaminopimelate containing) | -0.5000 | 7.32E-20 |
| P4-PWY: superpathway of L-lysine, L-threonine and L-methionine biosynthesis I | 1.4739 | 7.71E-20 |
| PWY-7220: adenosine deoxyribonucleotides de novo biosynthesis II | -0.9166 | 2.46E-19 |
| PWY-7222: guanosine deoxyribonucleotides de novo biosynthesis II | -0.9166 | 2.46E-19 |
| PWY0-166: superpathway of pyrimidine deoxyribonucleotides de novo biosynthesis (E. coli) | -0.8410 | 3.43E-19 |
| PWY-5097: L-lysine biosynthesis VI | -0.5330 | 4.27E-19 |
| MET-SAM-PWY: superpathway of S-adenosyl-L-methionine biosynthesis | 1.3296 | 1.38E-17 |
| PWY-6386: UDP-N-acetylmuramoyl-pentapeptide biosynthesis II (lysine-containing) | -0.4626 | 1.38E-17 |
| PWY-7184: pyrimidine deoxyribonucleotides de novo biosynthesis I | -0.9387 | 1.69E-17 |
| PWY-6700: queuosine biosynthesis | -0.7734 | 2.03E-17 |
| PWY-6387: UDP-N-acetylmuramoyl-pentapeptide biosynthesis I (meso-diaminopimelate containing) | -0.4627 | 2.13E-17 |
| HOMOSER-METSYN-PWY: L-methionine biosynthesis I | 1.3669 | 7.66E-17 |
| PWY-5100: pyruvate fermentation to acetate and lactate II | 0.9142 | 7.66E-17 |
| PWY-5686: UMP biosynthesis | -0.4308 | 9.48E-17 |
| PWY-5104: L-isoleucine biosynthesis IV | 1.5835 | 4.13E-16 |
| GLCMANNANAUT-PWY: superpathway of N-acetylglucosamine, N-acetylmannosamine and N-acetylneuraminate degradation | 0.8735 | 2.49E-15 |
| CITRULBIO-PWY: L-citrulline biosynthesis | -1.6769 | 1.66E-14 |
| METSYN-PWY: L-homoserine and L-methionine biosynthesis | 1.1272 | 2.37E-14 |
| PWY-7197: pyrimidine deoxyribonucleotide phosphorylation | -0.9934 | 2.71E-14 |
| PWY-6936: seleno-amino acid biosynthesis | 1.5551 | 4.04E-14 |
| PWY-5177: glutaryl-CoA degradation | 1.1043 | 7.71E-14 |
| COBALSYN-PWY: adenosylcobalamin salvage from cobinamide I | 0.9571 | 2.29E-12 |
| PWY-6385: peptidoglycan biosynthesis III (mycobacteria) | -0.6529 | 3.78E-12 |
| PWY4FS-7: phosphatidylglycerol biosynthesis I (plastidic) | -1.2660 | 5.18E-12 |
| PWY4FS-8: phosphatidylglycerol biosynthesis II (non-plastidic) | -1.2662 | 5.18E-12 |
| TRPSYN-PWY: L-tryptophan biosynthesis | 1.0696 | 1.64E-11 |
| NONMEVIPP-PWY: methylerythritol phosphate pathway I | -0.4218 | 2.16E-11 |
| PWY-7663: gondoate biosynthesis (anaerobic) | -0.7107 | 2.32E-11 |
| ARO-PWY: chorismate biosynthesis I | 0.3958 | 2.45E-11 |
| PWY-5973: cis-vaccenate biosynthesis | -0.6024 | 9.58E-11 |
| SER-GLYSYN-PWY: superpathway of L-serine and glycine biosynthesis I | 0.8726 | 1.01E-10 |
| PWY66-409: superpathway of purine nucleotide salvage | 1.7486 | 2.04E-10 |
| PWY-7221: guanosine ribonucleotides de novo biosynthesis | -0.3679 | 3.70E-10 |
| PWY-5101: L-isoleucine biosynthesis II | -1.1905 | 7.58E-10 |
| PWY-5103: L-isoleucine biosynthesis III | 0.6512 | 9.87E-10 |
| PWY-7208: superpathway of pyrimidine nucleobases salvage | -0.6385 | 9.94E-10 |
| UNINTEGRATED | -0.2674 | 2.78E-09 |
| PWY-5188: tetrapyrrole biosynthesis I (from glutamate) | 0.7282 | 3.01E-09 |
| PWY-6168: flavin biosynthesis III (fungi) | -0.7451 | 3.01E-09 |
| BRANCHED-CHAIN-AA-SYN-PWY: superpathway of branched amino acid biosynthesis | 0.5747 | 4.56E-09 |
| PWY-6608: guanosine nucleotides degradation III | 0.6668 | 6.49E-09 |
| PWY-6507: 4-deoxy-L-threo-hex-4-enopyranuronate degradation | 0.8738 | 7.96E-09 |
| PWY-7211: superpathway of pyrimidine deoxyribonucleotides de novo biosynthesis | 1.0596 | 8.36E-09 |
| PWY-5484: glycolysis II (from fructose 6-phosphate) | -1.1659 | 1.66E-08 |
| PWY-724: superpathway of L-lysine, L-threonine and L-methionine biosynthesis II | -0.2899 | 2.05E-08 |
| SALVADEHYPOX-PWY: adenosine nucleotides degradation II | 0.7247 | 4.16E-08 |
| PWY-6163: chorismate biosynthesis from 3-dehydroquinate | 0.3559 | 6.26E-08 |
| GLYCOLYSIS: glycolysis I (from glucose 6-phosphate) | -1.1191 | 7.38E-08 |
| THISYNARA-PWY: superpathway of thiamin diphosphate biosynthesis III (eukaryotes) | 0.7693 | 7.75E-08 |
| COMPLETE-ARO-PWY: superpathway of aromatic amino acid biosynthesis | 0.2921 | 7.86E-08 |
| COA-PWY-1: coenzyme A biosynthesis II (mammalian) | -0.3038 | 2.14E-07 |
| PWY-6609: adenine and adenosine salvage III | 0.4931 | 2.87E-07 |
| PHOSLIPSYN-PWY: superpathway of phospholipid biosynthesis I (bacteria) | -0.9940 | 5.00E-07 |
| PWY-7242: D-fructuronate degradation | 0.7069 | 5.31E-07 |
| PWY-6737: starch degradation V | 0.3966 | 2.26E-06 |
| DENOVOPURINE2-PWY: superpathway of purine nucleotides de novo biosynthesis II | -0.3180 | 3.77E-06 |
| RIBOSYN2-PWY: flavin biosynthesis I (bacteria and plants) | -1.0604 | 4.12E-06 |
| PWY-7219: adenosine ribonucleotides de novo biosynthesis | -0.2471 | 5.33E-06 |
| PWY-6123: inosine-5'-phosphate biosynthesis I | -0.4060 | 2.83E-05 |
| NONOXIPENT-PWY: pentose phosphate pathway (non-oxidative branch) | 0.3436 | 3.72E-05 |
| PRPP-PWY: superpathway of histidine, purine, and pyrimidine biosynthesis | 0.5922 | 3.72E-05 |
| PWY-7400: L-arginine biosynthesis IV (archaebacteria) | 1.0251 | 3.72E-05 |
| PWY66-399: gluconeogenesis III | -0.8323 | 5.51E-05 |
| ARGSYN-PWY: L-arginine biosynthesis I (via L-ornithine) | 1.0212 | 6.69E-05 |
| DTDPRHAMSYN-PWY: dTDP-L-rhamnose biosynthesis I | 0.4120 | 7.72E-05 |
| PWY0-162: superpathway of pyrimidine ribonucleotides de novo biosynthesis | -0.4794 | 8.92E-05 |
| PWY0-1296: purine ribonucleosides degradation | 0.4771 | 0.0002 |
| TRNA-CHARGING-PWY: tRNA charging | -0.3140 | 0.0002 |
| PYRIDNUCSYN-PWY: NAD biosynthesis I (from aspartate) | -0.4258 | 0.0003 |
| CALVIN-PWY: Calvin-Benson-Bassham cycle | 0.2380 | 0.0007 |
| PWY-3001: superpathway of L-isoleucine biosynthesis I | 0.2331 | 0.0007 |
| DAPLYSINESYN-PWY: L-lysine biosynthesis I | 0.5731 | 0.0008 |
| ASPASN-PWY: superpathway of L-aspartate and L-asparagine biosynthesis | -0.3165 | 0.0009 |
| PENTOSE-P-PWY: pentose phosphate pathway | 0.7168 | 0.0011 |
| ANAEROFRUCAT-PWY: homolactic fermentation | -0.4479 | 0.0015 |
| GLUCUROCAT-PWY: superpathway of β-D-glucuronide and D-glucuronate degradation | 0.4099 | 0.0021 |
| PWY-6305: putrescine biosynthesis IV | 0.3636 | 0.0021 |
| PWY-7187: pyrimidine deoxyribonucleotides de novo biosynthesis II | -0.2434 | 0.0025 |
| UNMAPPED | -0.2065 | 0.0026 |
| PWY-6545: pyrimidine deoxyribonucleotides de novo biosynthesis III | -0.3523 | 0.0027 |
| PWY-6121: 5-aminoimidazole ribonucleotide biosynthesis I | -0.1781 | 0.0028 |
| ARGSYNBSUB-PWY: L-arginine biosynthesis II (acetyl cycle) | 0.8520 | 0.0072 |
| PWY-4242: pantothenate and coenzyme A biosynthesis III | -0.3855 | 0.0106 |
| PWY-6124: inosine-5'-phosphate biosynthesis II | 0.2940 | 0.0122 |
| PWY-1042: glycolysis IV (plant cytosol) | 0.2010 | 0.0174 |
| PWY-7383: anaerobic energy metabolism (invertebrates, cytosol) | -0.4610 | 0.0281 |
| PWY-7282: 4-amino-2-methyl-5-phosphomethylpyrimidine biosynthesis (yeast) | -0.2954 | 0.0352 |
| COA-PWY: coenzyme A biosynthesis I | 0.2571 | 0.0415 |

**C.**

| QFS versus CFS | | |
| --- | --- | --- |
| Function | Log2FoldChange | Adjusted P value |
| HISTSYN-PWY: L-histidine biosynthesis | 0.3596 | 0.0078 |
| PWY-2942: L-lysine biosynthesis III | -0.3412 | 0.0078 |
| PWY-5097: L-lysine biosynthesis VI | -0.3102 | 0.0078 |
| PWY-621: sucrose degradation III (sucrose invertase) | 0.7878 | 0.0078 |
| PWY0-845: superpathway of pyridoxal 5'-phosphate biosynthesis and salvage | -0.5096 | 0.0078 |
| PWY-6507: 4-deoxy-L-threo-hex-4-enopyranuronate degradation | 0.8208 | 0.0084 |
| GALACTUROCAT-PWY: D-galacturonate degradation I | 0.6312 | 0.0096 |
| PWY-5686: UMP biosynthesis | -0.2290 | 0.0096 |
| PWY-6700: queuosine biosynthesis | -0.4674 | 0.0096 |
| PWY-7242: D-fructuronate degradation | 0.6860 | 0.0096 |
| PYRIDOXSYN-PWY: pyridoxal 5'-phosphate biosynthesis I | -0.5891 | 0.0096 |
| PANTO-PWY: phosphopantothenate biosynthesis I | -0.3047 | 0.0098 |
| SER-GLYSYN-PWY: superpathway of L-serine and glycine biosynthesis I | 0.6520 | 0.0120 |
| GALACT-GLUCUROCAT-PWY: superpathway of hexuronide and hexuronate degradation | 0.5508 | 0.0121 |
| PWY-6317: galactose degradation I (Leloir pathway) | 0.4829 | 0.0222 |
| GLCMANNANAUT-PWY: superpathway of N-acetylglucosamine, N-acetylmannosamine and N-acetylneuraminate degradation | 0.5107 | 0.0293 |
| PWY-6527: stachyose degradation | 0.4988 | 0.0293 |
| DAPLYSINESYN-PWY: L-lysine biosynthesis I | 0.5640 | 0.0297 |
| PWY-7357: thiamin formation from pyrithiamine and oxythiamine (yeast) | 0.3720 | 0.0297 |
| PWY66-422: D-galactose degradation V (Leloir pathway) | 0.4609 | 0.0368 |
| PEPTIDOGLYCANSYN-PWY: peptidoglycan biosynthesis I (meso-diaminopimelate containing) | -0.1880 | 0.0398 |
| NONOXIPENT-PWY: pentose phosphate pathway (non-oxidative branch) | 0.3322 | 0.0420 |
| GLUCUROCAT-PWY: superpathway of β-D-glucuronide and D-glucuronate degradation | 0.4882 | 0.0421 |
