# Supplementary material for: Multi-omics examination of Q fever fatigue syndrome identifies similarities with chronic fatigue syndrome
Source: J Transl Med. 2020 Nov 26;18:448. doi: 10.1186/s12967-020-02585-5 (PMC7690002; doi:10.1186/s12967-020-02585-5)
Supplement: Supplementary file 8 — Additional file 8: Figure S5. Global correlation pattern of chronically fatigued patients. Global correlation pattern by means of average clustering showing significantly different correlations between gut microbiome and metabolites (FDR adjusted P ≤ 0.05) in chronically fatigued patients, i.e., QFS and CFS. The global correlation pattern exposes a correlation between Bifidobacterium_adolescentis and N-docosahexaenoyl GABA, and Subdoligranulum_unclassified and Arbekacin. QFS Q fever fatigue syndrome, CFS chronic fatigue syndrome. [file 12967_2020_2585_MOESM8_ESM.pdf]

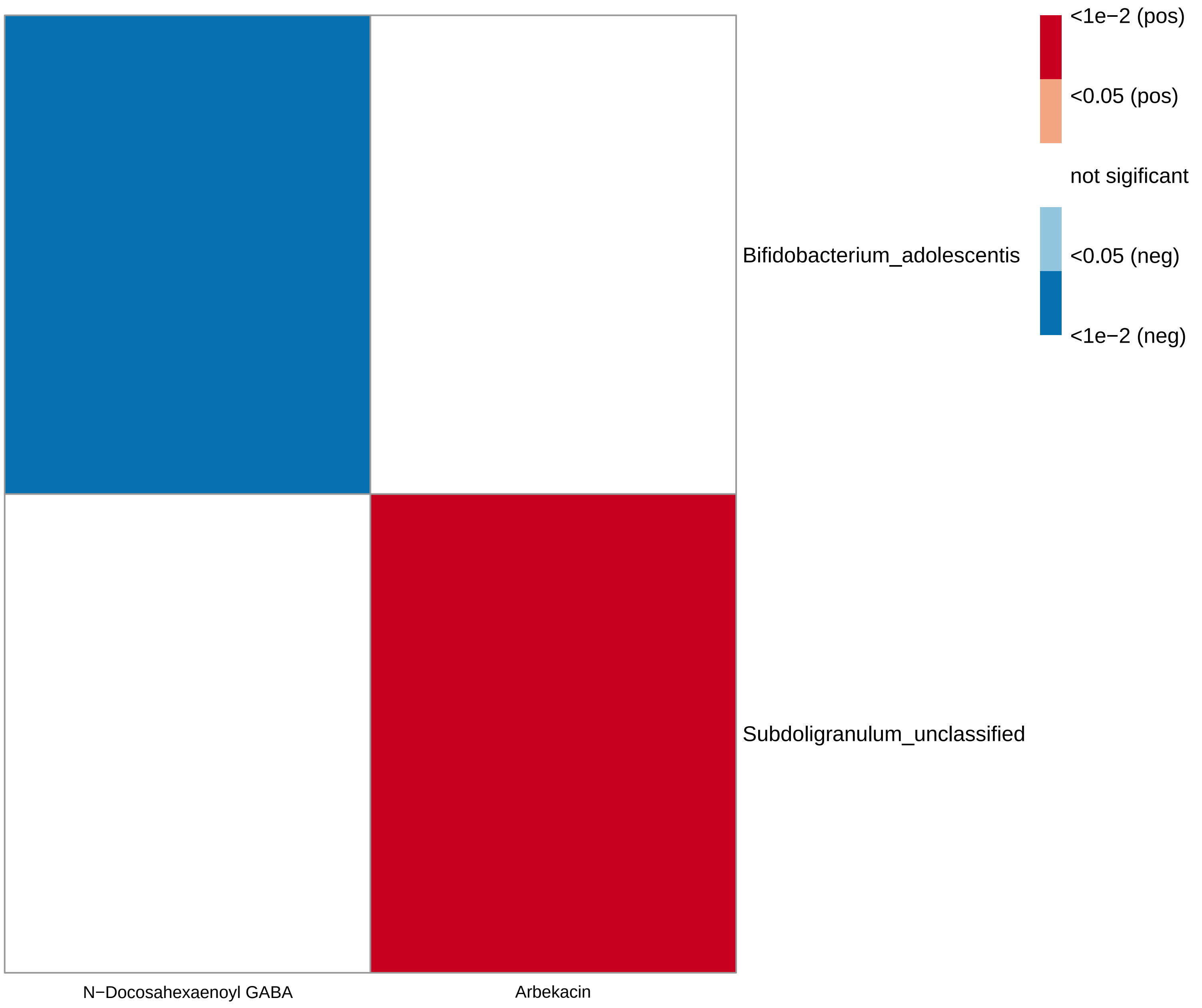

<1e−2 (pos)
<0.05 (pos)
not sigificant
<0.05 (neg)
<1e−2 (neg)
Bifidobacterium_adolescentis
Subdoligranulum_unclassified
N−Docosahexaenoyl GABA
Arbekacin
